# Leveraging Stereochemistry to Optimize the Properties of Polyhydroxyalkanoates

**DOI:** 10.1021/jacs.6c08149

**Published:** 2026-06-17

**Authors:** Morgan S. Young, Yolanda Rusconi, Anne M. LaPointe, Ivan Keresztes, Giovanni Talarico, Geoffrey W. Coates

**Affiliations:** † Department of Chemistry and Chemical Biology, Baker Laboratory, 5922 Cornell University, Ithaca, New York 14853-1301, United States; ‡ 657489 Scuola Superiore Meridionale, Largo San Marcellino, 80138 Naples, Italy; § Department of Chemical Sciences, University of Naples Federico II, Via Cintia, 80126 Naples, Italy

## Abstract

The recycling of low-density polyethylene (LDPE) is challenging due to difficulties with sorting and contamination, leading to environmental harm. Polyhydroxyalkanoates (PHAs) are at the forefront of high-performance biodegradable alternatives to olefinic plastics, but few offer LDPE-like properties such as low strength and crystallinity while maintaining high ductility and thermal stability. Herein, we report a series of isoenriched *trans*-poly­(3-hydroxy-2-methylbutyrates) (*trans*-PHMBs) with tunable mechanical and thermal properties. These polymers were synthesized through ring-opening polymerization of racemic *trans*-3,4-dimethylpropiolactone (*rac-trans*-DMPL), sourced from C1 and C4 feedstocks, using a new class of “sandwich” *C*_2_ symmetric *rac*-(^Ar^BDI*)­ZnO^*i*^Pr catalysts (where BDI = β-diketiminate). Variation of aromatic groups (Ar) and polymerization temperature yielded *mm*%s between 45–79% and melting temperatures (*T*_m_) between 141–174 °C. *trans*-PHMB with intermediate isotacticities of 73 and 75 *mm*% exhibit similar stress–strain profiles to LDPE, indicating that these polymers have the potential to serve as higher melting, degradable substitutes for LDPE.

## Graphical abstract

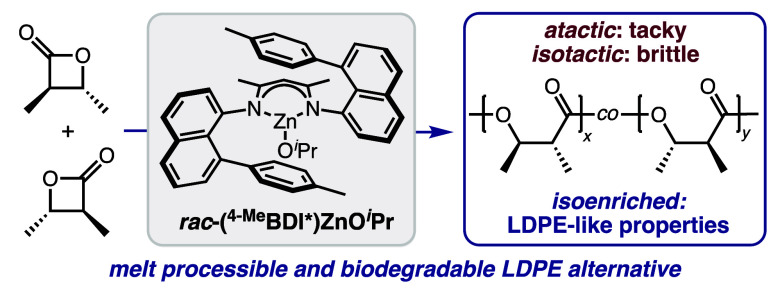

## Introduction

Low-density polyethylene (LDPE) accounts for approximately 12% of all plastic produced globally.^1^ Due to its low strength, low crystallinity, chemically inert hydrocarbon backbone, and excellent moisture barrier properties, LDPE is used as a thin coating on biodegradable materials, as landscape films, and in many other single use applications.^1–3^ Since the majority of LDPE is not recycled due to challenges associated with collection, sorting, and contamination, the development of biodegradable alternatives to LDPE is imperative to mitigate environmental damage.^1,4,5^

While poly­(butylene adipate terephthalate) (PBAT) is considered a biodegradable substitute for LDPE, it exhibits minimal ocean degradability and produces degradation products with significant plant and soil toxicity.^6,7^ Alternatively, polyhydroxyalkanoates (PHAs) are naturally produced and degraded by bacteria into H_2_O and CO_2_.^8–10^ The most studied PHA, (*R*)-poly­(3-hydroxybutyrate) ((*R*)-PHB, Scheme 1A) is semicrystalline, with a high melting temperature (*T*_m_ = ∼175 °C) and excellent ultimate tensile strength (σ_B_ = 30–40 MPa).^11–15^ However, (*R*)-PHB is brittle and exhibits low thermal stability resulting in a narrow melt processing window.^11,12,16^

**Scheme 1:**
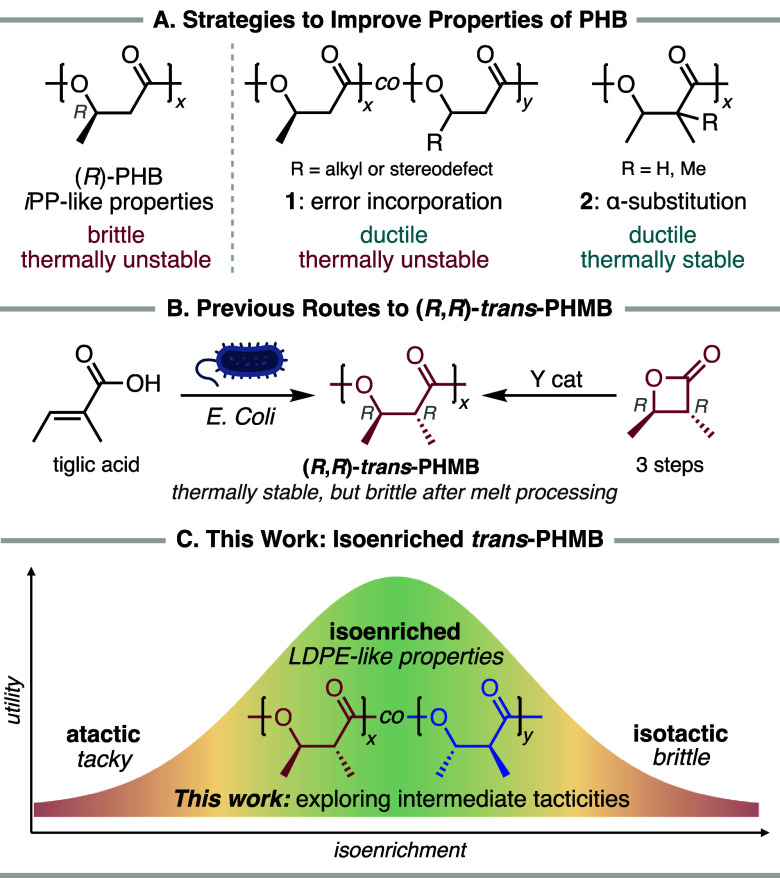
(A) Strategies to Improve PHA Properties; (B) Previous Routes to (*R,R*)-*trans*-PHMB; (C) This Work: Isoenriched *trans-*PHMB with LDPE-like Mechanical Properties

One strategy to enhance PHB properties is by incorporating stereodefects or longer alkyl substituents to decrease crystallinity thereby improving ductility and lowering *T*_m_ (Scheme 1A).^17–25^ For example, Rieger and co-workers synthesized isoenriched PHB with an *m*% (percent of ester linkages with a *meso* configuration) of 84% and enhanced ductility (elongation at break (ε_B_) = 392%).^26^ Simultaneously, the introduction of stereoerrors reduced the *T*_m_ from 175 to 140 °C, enabling a broader melt processing window. The second strategy enhances thermal stability through the substitution of the α carbon of PHAs (Scheme 1A).^27–34^ In PHB, the arrangement of acidic α-carbonyl protons and β-acetoxy leaving groups facilitates elimination reactions which reduce molecular weight and deteriorate mechanical properties.^16^ The addition of a *cis*-methyl or geminal dimethyls to the α carbon of PHB suppresses chain scission, increasing the degradation temperature (*T*_d_) from 241 °C to ∼275 °C and ∼325 °C, respectively.^12,29,32^ Despite these advances, no thermally stable PHA exhibits LDPE-like mechanical properties.

A particularly promising PHA for achieving a biodegradable LDPE alternative is (*R,R*)-*trans*-poly­(3-hydroxy-2-methylbutyrate) (*trans*-PHMB), a thermally stable PHA (*T*_d_ = 283 °C) which can be synthesized through bacterial fermentation of tiglic acid (Scheme 1B).^30,35^ While chloroform-cast (*R,R*)-*trans*-PHMB films exhibit mechanical properties similar to isotactic polypropylene (σ_B_ = 37 MPa, ε_B_ = 520%), a chemically synthesized sample (Scheme 1B) was too brittle for tensile testing after melt pressing.^30,35^ We hypothesized that introducing stereoerrors into *trans*-PHMB would reduce crystallinity to (1) improve melt processability and (2) soften the material to mimic the mechanical properties of LDPE (Scheme 1C).

The ring-opening polymerization (ROP) of *rac-trans*-3,4-dimethylpropiolactone (*rac-trans*-DMPL) is an atom-economical route to *trans-*PHMB polymers with tunable isotacticities. *Rac-trans*-DMPL is synthesized through sequential epoxidation and catalytic carbonylation of *cis*-2-butene, two processes amenable to scaleup.^32,36^ The *cis*-2-butene can be selectively synthesized through *Z*-selective dimerization of ethylene or hydrogenation of 2-butyne.^37–39^ For the polymerization, we aimed to design a racemic catalyst that can quantitatively convert *rac-trans*-DMPL into semicrystalline isoenriched *trans*-PHMB, as this would be the most practical and cost-efficient strategy in an industrial setting. To achieve these requirements, we investigated earth abundant metal and highly active zinc β-diketiminate alkoxide (BDIZnOR) catalyst frameworks. This strategy enabled access to high molecular weights (>100 kDa) necessary for the investigation of mechanical properties.^40–42^ Furthermore, the tunability of BDIZnOR frameworks was leveraged to produce *trans*-PHMB polymers with varying degrees of isoenrichment and their resulting thermal and mechanical properties were systemically studied.

## Results and Discussion

First, an efficient but nonstereoselective catalyst for β-butyrolactone ROP,^40^ [(^*i*Pr^BDI)­ZnO^*i*^Pr]_2_ (Scheme 2), was tested for the polymerization of *trans*-DMPL. Atactic *trans-*PHMB (*mm*% = 20%, Table S6; where *mm*% is the percent of triads with a *meso* configuration across both ester linkages) was obtained, indicating that *C*_2*v*_ symmetry does not invoke stereoselectivity. To improve stereocontrol, we modified the BDI framework by (1) introducing steric bulk in the axial positions and (2) imparting chirality via *C*_2_ symmetry about the metal center, a strategy motivated by group IV metallocene catalysts for isotactic polypropylene synthesis.^43^ Inspired by the “sandwich” diimine ligands reported by Brookhart and Daugulis, we synthesized a series of *C*_2_ symmetric BDI*H ligands using 8-aryl-1-aminonaphthalenes.^44,45^ Six *rac*-(^Ar^BDI*)­ZnO^*i*^Pr complexes (Scheme 2) with varying 8-aryl groups (Ar) were synthesized in a one-pot procedure by complexation of the ligand with Zn­(N­(SiMe_3_)_2_)_2_ at 150 °C, followed by addition of ^*i*^PrOH. After recrystallization at −25 °C, the desired *rac*-(^Ar^BDI*)­ZnO^*i*^Pr complexes were isolated in good yield (59–84% yield).

**Scheme 2:**
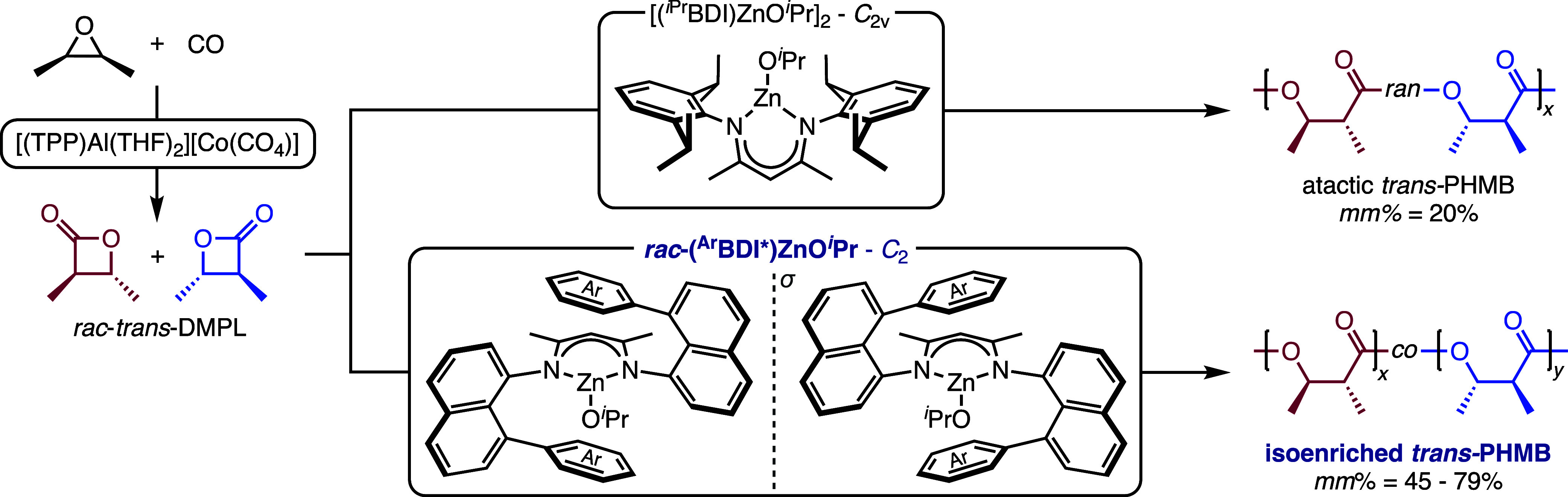
Isoselective Polymerization Design Strategy^a^

a TPP = *meso*-tetraphenylporphyrinato.

The catalyst with Ar = 4-Me-Ph (*rac*-(^4‑Me^BDI*)­ZnO^*i*^Pr) was examined, and it yielded isoenriched *trans*-PHMB with a *mm*% of 73 at 25 °C (Table 1, entry 2). Evaluation of the stereoerrors in the ^13^C­{^1^H} NMR spectra of this polymer suggest a primarily chain-end stereocontrol mechanism that would result in stereoblock *trans*-PHMB (See SI, section 3.2). Additionally, *rac*-(^4‑Me^BDI*)­ZnO^*i*^Pr is ∼20 times faster than [(^*i*Pr^BDI)­ZnO^*i*^Pr]_2_, reaching 96% and 13% conversion of 2000 equiv *trans*-DMPL in 18 h, respectively (Table S5). The catalyst *rac*-(^4‑Me^BDI*)­ZnO^*i*^Pr can also access *trans*-PHMB with varying isotacticities through temperature modulation, as *mm*%s vary between 77 and 66% when *T*_rxn_ = 0–50 °C (Table 1, entries 1–4). At these higher temperatures, the dispersities remain narrow, suggesting that minimal transesterification and elimination side reactions occur. Moreover, introduction of chain-transfer agent (CTA) enables *rac*-(^4‑Me^BDI*)­ZnO^*i*^Pr to perform immortal and stereoselective ROP at catalyst loadings as low as 0.02 mol %.^41^ Indeed, using a [*trans-*DMPL]_0_:[catalyst]_0_:[^*i*^PrOH]_0_ ratio of 5000:1:4 yielded isoenriched *trans*-PHMB (*mm*% = 73%; *M*_n_ = 93.9 kDa, *Đ* = 1.10; Table S10). Overall, *rac*-(^4‑Me^BDI*)­ZnO^*i*^Pr can access high molecular weights needed for good mechanical properties at low loadings while maintaining stereoselectivity.

**Table 1:**
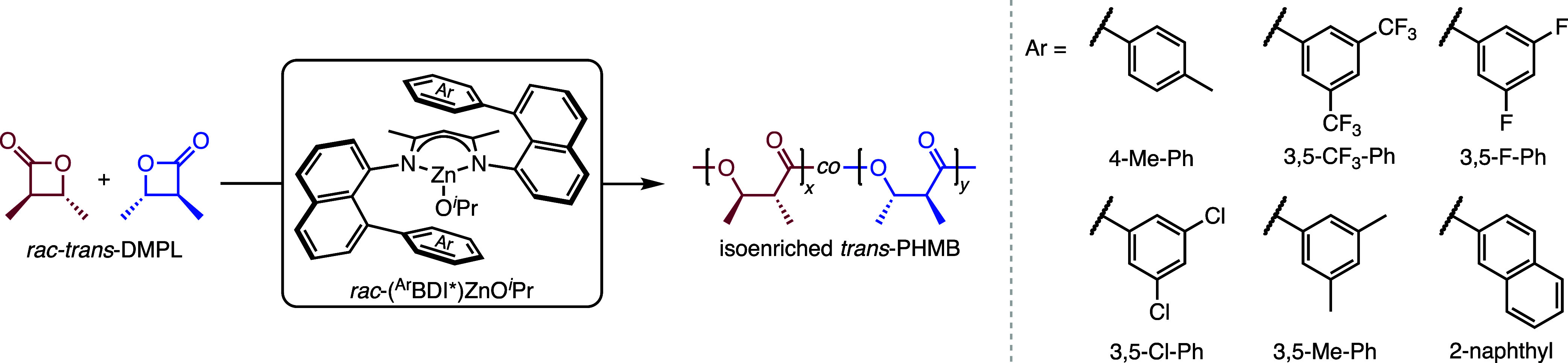
Polymerization of *rac*-*trans*-DMPL by *rac*-(^Ar^BDI*)­ZnO^*i*^Pr Complexes

entry^a^	Ar	*T*_rxn_ (°C)	conv. (%)^b^	*M*_n,theo_ (kDa)	*M*_n,SEC_ (kDa)^c^	*Đ* ^c^	*mm*%^d^	*T*_m_ (°C)^e^
1	4-Me-Ph	0	97	19.4	27.3	1.14	77	170/150
2	4-Me-Ph	25	>99	20.0	27.4	1.06	73	155/135
3	4-Me-Ph	40	>99	20.0	24.3	1.05	69	147/132
4	4-Me-Ph	50	>99	20.0	21.7	1.09	66	143
5	3,5-CF_3_-Ph	0	12	2.4	5.0	1.04	n.d.	n.d.
6	3,5-CF_3_-Ph	25	90	18.0	33.0	1.08	45	–
7	3,5-F-Ph	0	21	4.2	6.6	1.04	61	150/131^f^
8	3,5-F-Ph	25	>99	20.0	26.6	1.05	55	–
9	3,5-Cl-Ph	0	33	6.6	9.1	1.04	n.d	141/123
10	3,5-Cl-Ph	25	>99	20.0	35.4	1.05	54	–
11	3,5-Me-Ph	0	83	16.6	19.9	1.08	75	161/138
12	3,5-Me-Ph	25	>99	20.0	23.2	1.04	65	142
13	2-naphthyl	0	88	17.6	21.6	1.19	79	174/156
14	2-naphthyl	25	98	19.6	24.3	1.04	73	156/132

a [*rac*-trans-DMPL]_0_:[(^Ar^BDI*)­ZnO^*i*^Pr)]_0_ = 200:1, [*trans*-DMPL]_0_ = 4 M in PhMe, *t*_rxn_ = 18 h.

b Determined by ^1^H NMR spectroscopic analysis comparing the relative integration of polymer and residual monomer.

c Determined by SEC in THF at 30 °C, calibrated relative to monodisperse polystyrene standards.

d Determined by ^13^C­{^1^H} NMR spectroscopic analysis.

e Determined by DSC, polymorph 1/polymorph 2.

f Due to the low molecular weight of this sample, the DSC data was omitted from subsequent thermal properties analysis. n.d. = not determined

We hypothesized that introducing different steric and electronic environments around zinc, by changing Ar, would enable access to a broader scope of isoenrichments. Intriguingly, when Ar = 3,5-CF_3_-Ph, a drop in isoselectivity (45 *mm*% at 25 °C) and a reduction in activity (Table 1, entries 5 and 6) was observed. We theorize that the electron-withdrawing substituents increase the Lewis acidity of the Zn center, strengthening Zn-alkoxide coordination. The reactivity of the propagating alkoxide is therefore reduced and results in a slower monomer insertion step. Such an effect may also contribute to the reduced stereoselectivity observed for the more electron-deficient systems. Supporting this hypothesis, as the strength of the electron withdrawing groups in the *meta* positions was decreased from F to Cl to Me, the *mm*% content increased to 55, 54, and 65 *mm*%, respectively (Table 1, entries 8, 10, and 12). A corresponding enhancement in activity was also noted at 0 °C across the series (Table 1, entries 7, 9, 11, and 13). Notably, the catalyst with Ar = 3,5-Me-Ph exhibited a 10 *mm*% increase in isoselectivity upon lowering the temperature to 0 °C compared to a 4 *mm*% increase when Ar = 4-Me-Ph (Table 1, entries 1, 2, 11, and 12). We suspect this observation is due to a higher barrier for 3,5-Me-Ph rotation which is enhanced at lower temperatures, thus rigidifying the catalyst increasing stereoselectivity.

Based on these results, we targeted *trans*-PHMB with *mm*%s greater than 77 by designing a catalyst with a steric environment intermediate between 4-Me-Ph and 3,5-Me-Ph. At 0 °C, these two catalysts exhibit the highest *mm*% and the greatest enhancement of isoselectivity (compared to 25 °C), respectively. We envisioned that an intermediate steric environment could be furnished by installing an asymmetric 2-naphthyl group, which could improve isoselectivity. At 25 °C, the 2-naphthyl substituted catalyst exhibited identical isoselectivity to the 4-Me-Ph catalyst (*mm*% = 73%; Table 1, entries 2 and 14). Gratifyingly, at 0 °C, *rac*-(^2‑naphthyl^BDI*)­ZnO^*i*^Pr yielded the highest isotacticity (79 *mm*%) (Table 1, entry 13). This polymer has a moderately broadened dispersity, which we attribute to slightly different rates of polymerization of the three rotational isomers (Figures S80 and S81).

By modifying reaction temperature and ligand stereoelectronics, we successfully synthesized *trans*-PHMB polymer samples spanning a broad range of isotacticities (45–79 *mm*%), enabling a systematic evaluation of the influence of tacticity on thermal and mechanical properties. Analysis by DSC revealed a transition from amorphous to semicrystalline between 55 and 65 *mm*% as demonstrated by the appearance of an endothermic melting transition (Table 1, entries 8 and 12). For all tested samples (between 65 and 79 *mm*%), a linear increase in *T*_m_ and *mm*% was observed (Figure 1). Additionally, comparison of *rac-trans*-PHMB prepared from racemic monomer and racemic catalyst to enantioenriched *trans*-PHMB, revealed minimal differences in *T*_m_s (Figure S34). This observation suggests that the racemic samples do not undergo significant stereocomplexation arising from their stereoblock microstructure, despite the previous reports of stereocomplexation in perfectly isotactic *trans*-PHMB.^30^

**Figure 1:**
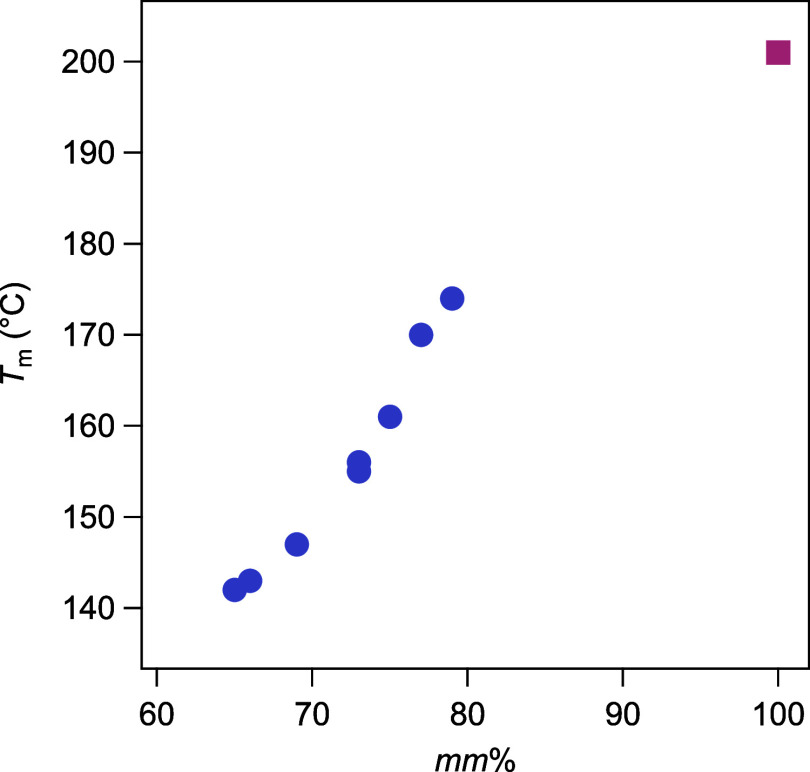
Relationship between *T*_m_ and *mm*% for isoenriched *trans*-PHMB samples in Table 1. *T*_m_ of 100 *mm*% sample is obtained from literature.^30,35^

We then assessed the thermal stability of isoenriched *trans*-PHMB by isothermally heating a sample at 170 °C under a N_2_ atmosphere. While the *M*_n_ of PHB drops by ca. 50% in 30 min of heating, isoenriched *trans*-PHMB exhibited minimal change in *M*_n_ (105.4 kDa to 99.1 kDa; Figure S35) with negligible change in *Đ*.^16,35^ This high thermal stability allows melt-pressing of *trans*-PHMB without significant molecular weight decrease, allowing systematic evaluation of the relationship between isotacticity and mechanical properties.

Next, we systematically studied the influence of isotacticity on the mechanical properties of *trans*-PHMB. High molecular weight samples (*M*_n_ ≈ 100 kDa) with varying isoenrichments (68, 73, 75, and 78 *mm*%) were melt pressed into dog bone shaped specimens and subjected to uniaxial tensile testing (Figure 2 and Tables S8 and S9). The least isotactic sample (68 *mm*%) exhibited the lowest stiffness (Young’s modulus, *E* = 58 ± 4 MPa) and no distinct yield point, consistent with its reduced crystallinity. The material showed remarkable ductility (ε_B_ = 1030 ± 60%) and pronounced strain hardening. Increasing isotacticity to 73 and 75 *mm*% resulted in the appearance of a clear yield point (yield stress, σ_Y_ = 8.1 ± 0.4 MPa, 8.9 ± 0.4 MPa, respectively) and approximately doubled the modulus (*E* = 120 ± 10 MPa in both cases). Although elongation at break decreased (ε_B_ = 680 ± 130% and 750 ± 40%), both materials retained appreciable ductility and exhibited strain hardening (σ_B_ = 9.9 ± 1.2 and 13.00 ± 0.03 MPa). These intermediate compositions display tensile profiles closely resembling LDPE, balancing deformability with moderate mechanical resistance and yield behavior. Further increasing isotacticity to 78 *mm*% produced the stiffest material (*E* = 130 ± 10 MPa, σ_Y_ = 10.0 ± 0.5 MPa), and reduced elongation at break (540 ± 70%), indicating increased crystallinity.

**Figure 2:**
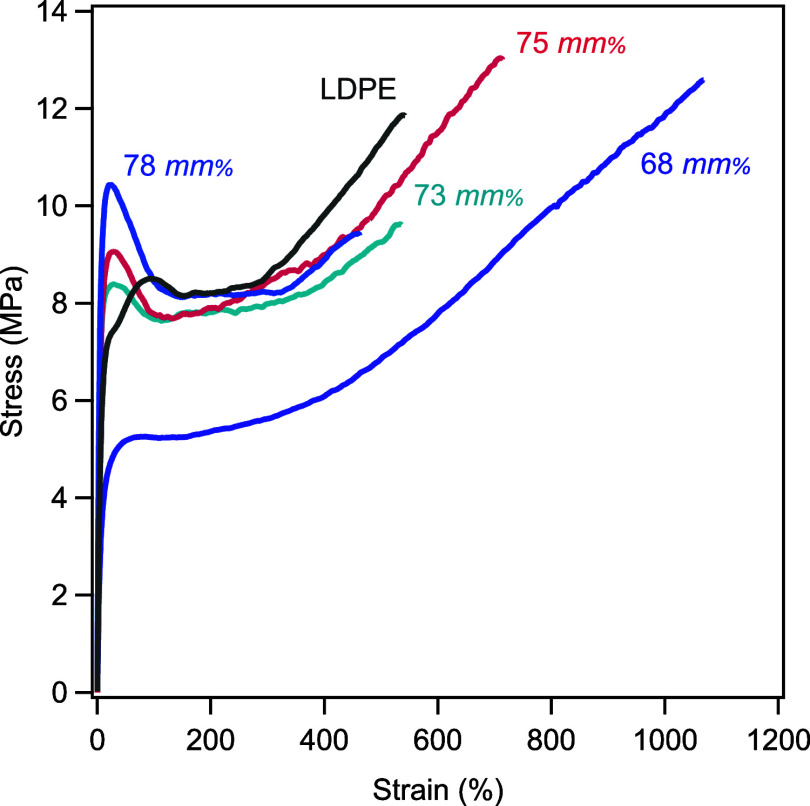
Stress–strain curves from uniaxial tensile testing of isoenriched *trans*-PHMB samples.

Collectively, these results demonstrate that modest variations in tacticity produce pronounced changes in mechanical behavior. Lower isotacticity yields softer, highly extendible materials, whereas higher isotactic enrichment increases stiffness and yield strength at the expense of ductility. Importantly, intermediate isotactic compositions (73–75 *mm*%) achieve an optimal balance of properties, closely mimicking the tensile response of LDPE. These findings underscore stereochemical control as a powerful design parameter for modulation of mechanical properties.

## Conclusions

In summary, we demonstrated that the thermal and mechanical properties of *trans*-PHMB can be tuned with slight variations to the stereoregularity. High molecular weight polymers with a broad range of isoenrichments (*mm*% = 45–79%) were synthesized by ROP of *trans*-DMPL catalyzed by a new class of *C*_2_ symmetric “sandwich” Zn BDI complexes (*rac*-(^Ar^BDI*)­ZnO^*i*^Pr). Isoenriched *trans*-PHMB exhibits excellent thermal stability under melt conditions, and mechanical testing revealed the samples with 73 and 75 *mm*% display tensile properties similar to LDPE while exhibiting substantially higher melting temperatures than LDPE. This work will inform the design of future stereoselective lactone polymerization catalysts and provides a platform to tune mechanical properties through tacticity manipulation. Future work will focus on investigating the mechanism of stereocontrol for these *rac-*(^Ar^BDI*)­ZnO^*i*^Pr catalysts.

## Supplementary Material



## Notes

Experimental procedures; material characterization; spectral data (PDF)
