# Supplementary material for: Leveraging Stereochemistry to Optimize the Properties of Polyhydroxyalkanoates
Source: J Am Chem Soc. 2026 Jun 17;148(25):26768–73. doi: 10.1021/jacs.6c08149 (PMC13339154; doi:10.1021/jacs.6c08149)
Supplement: Supplementary file 1 [file ja6c08149_si_001.pdf]

Supporting Information for:

Leveraging Stereochemistry to Optimize the Properties of Polyhydroxyalkanoates

Morgan S. Young[‡,a], Yolanda Rusconi[‡,b,c], Anne M. LaPointe[a], Ivan Keresztes[a], Giovanni Talarico[b,c] and Geoffrey W. Coates[a*]

[a] Department of Chemistry and Chemical Biology, Baker Laboratory, Cornell University, Ithaca, New York 14853-1301, United States

[b] Scuola Superiore Meridionale, Largo San Marcellino, 80138 Naples, Italy

[c] Department of Chemical Sciences, University of Naples Federico II, Via Cintia, 80126 Naples, Italy

Table of Contents

1 Materials and methods 1
 1.1 General considerations 1
 1.2 Materials 2
 1.3 Characterization methods 3
2 Synthetic procedures 4
 2.1 8-Arylnaphthylamine syntheses 4
 2.2 C_2 Symmetric β-diketimine ligand synthesis (^{Ar}BDI*H) 8
 2.3 (^{Ar}BDI*)ZnO^{i}Pr Complex synthesis 11
 2.4 Synthesis of [(^{iPr}BDI)ZnO^{i}Pr]$_2$ 14
 2.5 Synthesis of enantioenriched (*R*,*R*)-*trans*-DMPL 15
 2.6 General polymerization procedure 16
 2.7 Synthesis of syndioenriched *trans*-PHMB 17
3 Experimental details and polymer characterization 18
 3.1 Determination of stereoerrors in ^{13}C{^{1}H} NMR 18
 3.2 Example tacticity calculation 23
 3.3 Optimization of polymerization conditions 24
 3.4 Comparison of [(^{iPr}BDI)ZnO^{i}Pr]$_2$ and ($^{4\text{-}Me}$BDI*)ZnO^{i}Pr rates 25
 3.5 Synthesis of atactic and syndioenriched *trans*-PHMB 26
 3.6 Polymer characterization of high molecular weight samples 27
 3.7 Other polymerizations catalyzed by *rac*-($^{4\text{-}Me}$BDI*)ZnO^{i}Pr 28
 3.8 SEC chromatograms 29
 3.9 DSC thermograms 35
 3.10 Thermal stability 46
 3.11 Preparation of tensile samples 47

3.12 Tensile properties 48
3.13 Chiral GC chromatograms 52
4 NMR Spectra 53
4.1 8-Arylnaphthylamines 53
4.2 *rac*-BDI*H ligands 61
4.3 *rac*-(^{Ar}BDI*)Zn(O^{i}Pr) complexes 69
4.4 [(^{iPr}BDI)ZnO^{i}Pr]$_2$ spectra 77
4.5 Selected *trans*-PHMB ^{13}C{^{1}H} NMR spectra 78
5 References 87

1 Materials and methods

1.1 General considerations

Unless otherwise indicated, all reactions and manipulations of air and/or water sensitive compounds were carried out under a dry nitrogen atmosphere using an MBraun glovebox or standard Schlenk techniques. Toluene, hexanes and THF for air/moisture sensitive reactions were purchased from Fisher, sparged with ultrahigh purity (UHP) grade nitrogen, passed through two columns containing reduced copper (Q-5) and alumina and dispensed into an oven-dried Straus flask. The solvent was brought into the glovebox and stored over activated 3 Å sieves for 24 h before use. CH_2Cl_2 for air/moisture sensitive reactions were purchased from Fisher, sparged with UHP grade nitrogen, passed through two columns of alumina and dispensed into an oven-dried Straus flask. The CH_2Cl_2 was then cannula transferred onto freshly ground CaH_2 under an N_2 atmosphere and stirred for 3 d. The CH_2Cl_2 was then vacuum transferred, degassed with three freeze-pump-thaw cycles and brought into the glovebox where it was stored over activated neutral alumina beads (BASF F200) for 24 h before use. Et_2O for air/moisture sensitive reactions was purchased from Fisher, sparged with argon, passed through two columns of alumina and dispensed into an oven-dried Straus flask. Chloroform-*d* ($CDCl_3$) and benzene-d_6 (C_6D_6) were purchased from Cambridge Isotope Laboratories. $CDCl_3$ was used without further purification. C_6D_6 was sparged with nitrogen for 15 min before being stored in a glovebox over alumina beads for 24 h before use. Otherwise, solvents (hexanes, EtOAc, CH_2Cl_2, EtOH, MeOH, toluene) were purchased from commercial sources and used without further purification. Flash column chromatography was performed using silica gel (Sillicycle, particle size 40 - 64 μm, 230 - 400 mesh).

1.2 Materials

CaH_2 was purchased from Acros Organics (10 - 100 mm pieces) and was ground into a fine powder using a mortar and pestle immediately before use. Anhydrous isopropanol was purchased from Millipore Sigma and was dried over CaH_2 for 3 d under an N_2 atmosphere, vacuum transferred and degassed with three freeze-pump-thaw cycles before use. The *cis*-2-butene oxide (97%, Synquest) was dried over CaH_2 for 3 d, vacuum transferred, degassed via three freeze-pump-thaw cycles, and stored at 25°C under nitrogen before use. Carbon monoxide was purchased from Matheson (research purity >99.999% min purity) and used as received. 1-Aminonaphthalene and tris(dibenzylideneacetone)dipalladium(0) were purchased from Oakwood chemicals and used as received. *p*-Toluenesulfonic acid monohydrate was purchased from Acros Organics and used as received. Palladium (II) acetate ($Pd(OAc)_2$), silver (I) acetate (Ag(OAc)), 4-iodotoluene, *n*-butyllithium (1.6 M in hexanes), chlorotrimethylsilane, trimethyl borate and triphenyl phosphine were purchased from Sigma Aldrich and used as received. 1-Iodo-3,5-dimethylbenzene was purchased from VWR international and used as received. 1,3-difluoro-5-iodobenzene and 1,3-bis(trifluoromethyl)bromobenzene were purchased from Lancaster Chemicals and used as received. 1,3-dichloro-5-iodobenzene was purchased from Alfa Aeser and used as received. Acetylacetone was purchased from Fluka Chemicals and used as received. The carbonylation catalysts, bis(tetrahydrofuran)-*meso*-tetraphenylporphyrinato aluminum tetracarbonyl cobaltate, $[(TPP)Al(THF)_2][Co(CO)_4]$ and $[(ArCH)_2(BINAM)]AlCl$, were synthesized as previously reported.[1,2] *rac*-*trans*-3,4-Dimethylpropiolactone (*trans*-DMPL) and *rac*-*cis*-3,4-dimethylpropiolactone were synthesized according to a modified literature procedure.[3] $LY(N(SiMe_3)_2)$ was synthesized as previously reported.[4] $Zn(N(SiMe_3)_2)_2$ was synthesized as previously reported.[5] The low density polyethylene (LDPE) reference was purchased from Scientific Polymer Products (M_n = 5.2 kDa, M_w = 113.8 kDa, $Đ$ = 22).

1.3 Characterization methods

Nuclear magnetic resonance (NMR) spectroscopy: All NMR spectra were acquired at 25 °C. ^{1}H, $^{13}C\{^{1}H\}$ and ^{19}F NMR spectra were recorded on a Bruker AVANCE III HD spectrometer with a broadband Prodigy cryoprobe at 25 °C (operating at 500 MHz for ^{1}H). The 2D band selective HSQCAD (^{1}H, ^{13}C) spectra were collected on a Varian INOVA 600 (operating at 600 MHz for ^{1}H) spectrometer with a Varian 5 mm inverse, triple-resonance probe head. For ^{1}H and $^{13}C\{^{1}H\}$ spectra, chemical shift data is reported in units of δ (ppm) relative to tetramethylsilane (TMS). ^{1}H NMR spectra were referenced to the residual solvent (7.26 ppm for $CDCl_3$ and 7.16 ppm for C_6D_6) and $^{13}C\{^{1}H\}$ NMR spectra were referenced to the deuterated solvent itself (77.16 ppm for $CDCl_3$ and 128.06 ppm for C_6D_6). NMR spectra were processed in MestReNova 14.3.2. NMR spectroscopic data are reported as follows: chemical shift, multiplicity (s = singlet, d = doublet, t = triplet, q = quartet, hept= heptet, m = multiplet, b = broad), coupling constants (Hz) and integration.

High resolution mass spectrometry (HRMS): Mass spectra were acquired on a DART-SVP (Direct Analysis in Real Time) ion source (IonSense, Saugus, MA) coupled to an Exactive Orbitrap mass spectrometer (Thermo Scientific, Bremen, Germany).

Size exclusion chromatography (SEC): SEC analyses were carried out using an Agilent 1260 Infinity SEC System equipped with an Agilent 1260 Infinity autosampler and a refractive index detector. The Agilent SEC system was equipped with a guard column (4.6 x 50 mm) and two Agilent PolyPore MiniMix columns (5-micron, 4.6 x 250 mm ID) which were eluted with THF at 30 °C at 0.3 mL/min and calibrated using monodisperse polystyrene standards. Prior to analysis, polymer samples (1 mg/mL THF) were gently heated to dissolve any residual polymer particles before being passed through a 0.45 µm PTFE syringe filter.

Chiral gas chromatography (Chiral GC): GC analysis to determine percent enantiomeric excess was done using a Hewlett Packard 6890 gas chromatograph equipped with a Supelco β-Dex 225 column (L × I.D. 30 m × 0.25 mm, df 0.25 µm) and a flame-ionization detector. Method for *trans*-DMPL: The injection port is set to 150 °C. After injection, the oven temperature increases from 50 °C to 200 °C at 8 °C/min. (*R,R*)-*trans*-DMPL is eluted at ~14.9 min, and (*S,S*)-*trans*-DMPL is eluted at ~15.2 min.

Differential scanning calorimetry (DSC): DSC analysis was performed on a Mettler Toledo Polymer DSC instrument under a flow of N_2. Polymer samples (2 – 5 mg) in crimped aluminum pans were prepared for each run. The samples were heated to 210 °C at 10 °C/min, then cooled to -70 °C at 10 °C/min, and finally heated from -70 °C to 210 °C at 10 °C/min. The T_c value was taken from the first cooling curve and/or second heat and the T_g and T_m values were taken from the second heating curve, all using the maximum value of the derivative of heat flow with respect to temperature using the STARe software.

Uniaxial tensile testing: Tensile tests were performed on a Shimadzu Autograph AGS-X series instrument with pneumatic grips and 500 N load cell. Tensile specimens were cut using an ASTM 37-4 standard die cutter. Tensile elongation measurements were performed under ambient conditions with a crosshead velocity of 10 mm/min until break.

2 Synthetic procedures
2.1 8-Arylnaphthylamine syntheses

Picolinamide directed C–H arylation

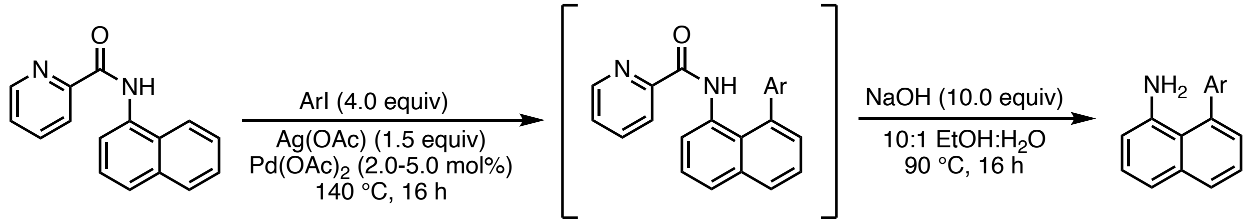

General procedure 1: Following a modified procedure by Daugulis and coworkers:[6,7] Aryl iodide (4.0 equiv), *N*-(naphthalen-1-yl)picolinamide[7] (1.0 equiv), Ag(OAc) (1.5 equiv) and $Pd(OAc)_2$ (2.0-5.0 mol%) were sequentially added to a flame-dried schlenk flask with stir bar under N_2 and heated to 140 °C for 16 h. The reaction was cooled to 25 °C, suspended in CH_2Cl_2, filtered and the collected salts washed with CH_2Cl_2 until the filtrate ran clear. The filtrate was condensed *in vacuo* in a round bottom flask. The resulting residue was mixed with NaOH (10 equiv), EtOH/H_2O (10:1, 0.25 M), fitted with a reflux condenser and stir bar and heated to 90 °C for 16 h. The reaction was cooled to 25 °C and mixed with equal parts H_2O and CH_2Cl_2. The layers were separated and the aqueous layer extracted with CH_2Cl_2 (2X). The combined organics were washed with brine, dried over $MgSO_4$, filtered and condensed *in vacuo*. The collected residue was purified with flash column chromatography.

8-(4-Methylphenyl)naphthalen-1-amine: Following the general procedure 1, *N*-(naphthalen-1-yl)picolinamide (4.39 g, 17.7 mmol, 1.00 equiv), 4-iodotoluene (15.6 g, 71.6 mmol, 4.05 equiv), Ag(OAc) (4.47 g, 26.7 mmol, 1.51 equiv) and $Pd(OAc)_2$ (0.20 g, 0.89 mmol, 0.050 equiv) were heated to 140 °C under N_2 for 16 h. After isolating the organics, the resulting residue was combined with NaOH (7.54 g, 189 mmol, 10.7 equiv), EtOH (60 mL) and H_2O (6.0 mL) and heated to 90 °C for 16 h. After workup the residue was purified by flash column chromatography (94:5:1 Hexanes:EtOAc:NEt_3) to yield the title compound as a red solid (3.2 g, 77%). **^{1}H NMR** (500 MHz, $CDCl_3$) δ 7.77 (dd, *J* = 8.2, 1.4 Hz, 1H), 7.38 (dd, *J* = 8.2, 7.0 Hz, 1H), 7.36 – 7.24 (m, 6H), 7.15 (dd, *J* = 7.0, 1.4 Hz, 1H), 6.63 (dd, *J* = 7.3, 1.3 Hz, 1H), 3.81 (bs, 2H), 2.44 (s, 3H). **^{13}C{^{1}H} NMR** (126 MHz, $CDCl_3$) δ 143.89, 140.67, 138.43, 137.35, 136.00, 129.24, 128.88, 128.66, 128.40, 126.60, 124.72, 121.01, 119.15, 111.36, 21.41. **HRMS** (DART-Orbitrap): m/z calculated for $C_{17}H_{16}N$ $[M+H]^+$ 234.1283, found 234.1278. Chemical shift data are in agreement with literature.[7]

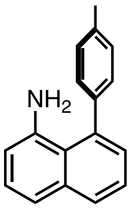

8-(3,5-Dimethylphenyl)naphthalen-1-amine: Following the general procedure 1, *N*-(naphthalen-1-yl)picolinamide (2.00 g, 8.05 mmol, 1.00 equiv), 1-iodo-3,5-dimethylbenzene (4.70 mL, 32.2 mmol, 4.00 equiv), Ag(OAc) (2.01 g, 12.1 mmol, 1.50 equiv) and $Pd(OAc)_2$ (0.038 g, 0.16 mmol, 0.020 equiv) were heated to 140 °C under N_2 for 16 h. After isolating the organics, the resulting residue was combined with NaOH (3.24 g, 80.9 mmol, 10.0 equiv), EtOH (30 mL) and H_2O (3.0 mL) and heated to 90 °C for 16 h. After workup the residue was purified by flash column chromatography (94:5:1 Hexanes:EtOAc:NEt_3) to yield the title compound as a dark red oil (1.54 g, 82%). **^{1}H NMR** (500 MHz, $CDCl_3$): δ 7.77 (dd, *J* = 8.2, 1.4 Hz, 1H), 7.38 (dd, *J* = 8.2, 7.0 Hz, 1H), 7.33 (dd, *J* = 8.1, 1.4 Hz, 1H), 7.28 (d, *J* = 7.5 Hz, 1H), 7.14 (dd, *J* = 7.0, 1.4 Hz, 1H), 7.08 (s, 2H), 7.07 (s, 1H), 6.62 (dd, *J* = 7.3, 1.4 Hz, 1H), 3.82 (bs, 2H), 2.38 (s, 6H). **^{13}C{^{1}H} NMR** (126 MHz, $CDCl_3$): δ 144.01, 143.59, 138.70, 137.73, 135.98, 129.18, 128.60, 128.08, 127.17, 126.58, 124.63, 120.83, 118.97, 111.15, 21.47. **HRMS** (DART-Orbitrap): m/z calculated for $C_{18}H_{18}N$ $[M+H]^+$ 248.1439, found 248.1422. Chemical shift data are in agreement with literature.[8]

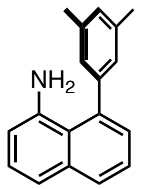

8-(3,5-Difluorophenyl)naphthalen-1-amine: Following the general procedure 1, *N*-(naphthalen-1-yl)picolinamide (2.10 g, 8.45 mmol, 1.00 equiv), 1-iodo-3,5-difluorobenzene (4.00 mL, 32.2 mmol, 3.81 equiv), Ag(OAc) (2.28 g, 13.7 mmol, 1.62 equiv) and $Pd(OAc)_2$ (0.036 g, 0.16 mmol, 0.019 equiv) were heated to 140 °C under N_2 for 16 h. After isolating the organics, the resulting residue was combined with NaOH (3.24 g, 81.0 mmol, 9.59 equiv), EtOH (30 mL) and H_2O (3 mL) and heated to 90 °C for 16 h. After workup the residue was purified by flash column chromatography (94:5:1 Hexanes:EtOAc:NEt_3) to yield the title compound as an orange solid (1.07 g, 50%). **^{1}H NMR** (500 MHz, $CDCl_3$) δ 7.82 (dd, *J* = 8.2, 1.5 Hz, 1H), 7.39 (dd, *J* = 8.2, 7.0 Hz, 1H), 7.37 – 7.28 (m, 2H), 7.15 (dd, *J* = 6.9, 1.4 Hz, 1H), 7.00 (dt, *J* = 6.2, 2.1 Hz, 2H), 6.88 (tt, *J* = 8.9, 2.3 Hz, 1H), 6.69 (dd, *J* = 7.3, 1.5 Hz, 1H), 3.73 (bs, 2H). **^{13}C{^{1}H} NMR** (126 MHz, $CDCl_3$) δ 162.41 (dd, *J* = 250.3, 13.0 Hz), 146.83 (t, *J* = 9.4 Hz), 143.19, 135.94, 129.70, 128.27, 126.97, 124.62, 120.37, 119.48, 112.94 – 112.62 (m), 112.03, 103.13 (t, *J* = 25.2 Hz). **^{19}F NMR** (470 MHz, $CDCl_3$) δ -109.53. **HRMS** (DART-Orbitrap): m/z calculated for $C_{16}H_{12}F_2N$ $[M+H]^+$ 256.0938, found 256.0919. Chemical shift data are in agreement with literature.[9]

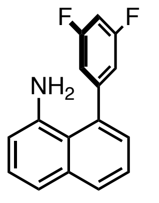

8-(3,5-Dichlorophenyl)naphthalen-1-amine: Following the general procedure 1, *N*-(naphthalen-1-yl)picolinamide (2.08 g, 8.38 mmol, 1.00 equiv), 1-iodo-3,5-dichlorobenzene (8.81 g, 32.3 mmol, 3.85 equiv), Ag(OAc) (2.22 g, 13.7 mmol, 1.59 equiv) and $Pd(OAc)_2$ (0.036 g, 0.16 mmol, 0.019 equiv) were heated to 140 °C under N_2 for 16 h. After isolating the organics, the resulting residue was combined with NaOH (3.24 g, 81.0 mmol, 9.67 equiv), EtOH (30 mL) and H_2O (3 mL) and heated to 90 °C for 16 h. After workup the residue was purified by flash column chromatography (94:5:1 Hexanes:EtOAc:NEt_3) to yield the title compound as a red solid (0.94 g, 39%). **^{1}H NMR** (500 MHz, $CDCl_3$) δ 7.81 (dd, *J* = 8.2, 1.4 Hz, 1H), 7.43 (t, *J* = 1.9 Hz, 1H), 7.41 – 7.33 (m, 5H), 7.31 (t, *J* = 7.7 Hz, 1H), 7.13 (dd, *J* = 7.0, 1.3 Hz, 1H), 6.69 (dd, *J* = 7.2, 1.4 Hz, 1H), 3.66 (bs, 2H). **^{13}C{^{1}H} NMR** (126 MHz, $CDCl_3$) δ 146.44, 143.23, 135.96, 135.51, 134.56, 129.77, 128.57, 127.97, 127.70, 126.99, 124.65, 120.31, 119.49, 112.02. **HRMS** (DART-Orbitrap): m/z calculated for $C_{16}H_{12}Cl_2N$ $[M+H]^+$ 288.0347, found 288.0326. Chemical shift data are in agreement with literature.[10]

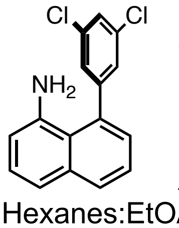

[1,2'-Binaphthalen]-8-amine: Following the general procedure 1, *N*-(naphthalen-1-yl)picolinamide (2.02 g, 8.14 mmol, 1.00 equiv), 2-iodonaphthalene (9.26 g, 32.2 mmol, 3.96 equiv, contains 0.26 equiv naphthalene), Ag(OAc) (2.04 g, 12.2 mmol, 1.50 equiv) and $Pd(OAc)_2$ (0.092 g, 0.41 mmol, 0.050 equiv) were heated to 140 °C under N_2 for 16 h. After isolating the organics, the resulting residue was combined with NaOH (3.48 g, 87.0 mmol, 10.7 equiv), EtOH (30 mL) and H_2O (3 mL) and heated to 90 °C for 16 h. After workup the residue was purified by flash column chromatography (1:1 Hexanes: CH_2Cl_2) to yield the title compound as a dark red solid (1.52 g, 69%). **^{1}H NMR** (500 MHz, $CDCl_3$) δ 8.01 – 7.86 (m, 4H), 7.83 (dd, *J* = 8.2, 1.4 Hz, 1H), 7.65 – 7.50 (m, 3H), 7.43 (dd, *J* = 8.3, 7.0 Hz, 1H), 7.37 (dd, *J* = 8.1, 1.3 Hz, 1H), 7.31 (t, *J* = 7.7 Hz, 1H), 7.24 (dd, *J* = 6.9, 1.4 Hz, 1H), 6.64 (dd, *J* = 7.3, 1.3 Hz, 1H), 3.71 (bs, 2H). **^{13}C{^{1}H} NMR** (126 MHz, $CDCl_3$) δ 143.95, 141.29, 138.34, 136.04, 132.98, 132.62, 128.94, 128.70, 128.26, 128.06, 127.94, 127.59, 127.57, 126.79, 126.75, 126.42, 124.74, 120.91, 119.15, 111.33. **HRMS** (DART-Orbitrap): m/z calculated for $C_{20}H_{16}N$ $[M+H]^+$ 270.1283, found 270.1260. Chemical shift data are in agreement with literature.[6]

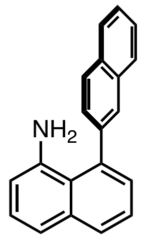

Amide directed arylation

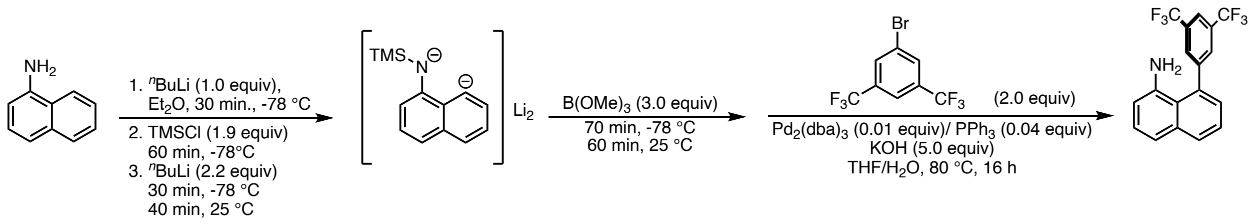

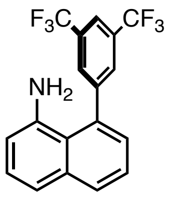

8-(3,5-Bis(trifluoromethyl)phenyl)naphthalen-1-amine: Following a modified procedure by Mecking and coworkers:[11] *n*-Butyl lithium (14 mL, 22.4 mmol, 1.10 equiv, 1.6 M in hexanes) was added dropwise to a solution of 1-aminonaphthalene (3.00 g, 21.0 mmol, 1.00 equiv) in Et_2O (50 mL) in a 3-neck round bottom flask equipped with a stir bar and reflux condenser at -78 °C. After stirring for 30 min at -78 °C, TMSCl (5.20 mL, 41.0 mmol, 1.95 equiv) was added dropwise and the reaction stirred at -78 °C for 60 min. A second portion of *n*-butyl lithium (29.0 mL, 46.4 mmol, 2.20 equiv) was added dropwise and the reaction stirred for 30 min at -78 °C followed by 40 min at 25 °C. The reaction flask was cooled back down to -78 °C and $B(OMe)_3$ (7.00 mL, 62.9 mmol, 3.00 equiv) was added dropwise. The reaction was stirred at -78 °C for 70 min, followed by an additional 60 min at 25 °C. The volatiles were removed *in vacuo* to yield a light-yellow solid which was dissolved/suspended in THF (40 mL). Then, KOH (5.90 g, 105 mmol, 5.00 equiv), 1-bromo-3,5-bis(trifluoromethyl)benzene (7.20 mL, 41.9 mmol, 2.00 equiv) and water (10 mL) were carefully added. Finally, a premade (stirred for 15 mins before addition) orange solution of $Pd_2(dba)_3$ (0.20 g, 0.21 mmol, 0.010 equiv) and PPh_3 (0.23 g, 0.86 mmol, 0.040 equiv) in THF (7 mL) was added. The reaction was refluxed at 80 °C for 16 h. After cooling to 25 °C, the reaction was diluted with water (30 mL) and added to a separatory funnel. The layers were separated and the aqueous layer was extracted with THF (2 X 50 mL). The combined organics were washed with $NaOH_{(aq)}$ (2 X 50 mL, 0.45 M), dried over $MgSO_4$, filtered and dried *in vacuo*. The isolated red oil was purified by flash column chromatography (80:20 hexanes:EtOAc) to isolate the title compound as a viscous red oil (4.21 g, 56%). **^{1}H NMR** (500 MHz, $CDCl_3$) δ 7.96 (s, 2H), 7.94 (s, 1H), 7.87 (dd, *J* = 8.2, 1.4 Hz, 1H), 7.47 – 7.38 (m, 2H), 7.35 (t, *J* = 7.7 Hz, 1H), 7.19 (dd, *J* = 7.0, 1.4 Hz, 1H), 6.73 (dd, *J* = 7.3, 1.4 Hz, 1H), 3.43 (bs, 2H). **^{13}C{^{1}H} NMR** (126 MHz, $CDCl_3$) δ 145.35, 142.95, 136.04, 135.16, 131.03 (q, *J* = 33.4 Hz), 130.20, 129.75 – 129.51 (m), 129.24, 127.15, 124.78, 123.44 (q, *J* = 272.8 Hz), 121.46 – 121.03 (m), 120.29, 119.91, 112.49. **^{19}F NMR** (470 MHz, $CDCl_3$) δ -62.70. **HRMS** (DART-Orbitrap): m/z calculated for $C_{18}H_{12}F_6N$ $[M+H]^+$ 356.0874, found 356.0847. Chemical shift data are in agreement with literature.[11]

2.2 C_2 Symmetric β-diketimine ligand synthesis (^{Ar}BDI*H)

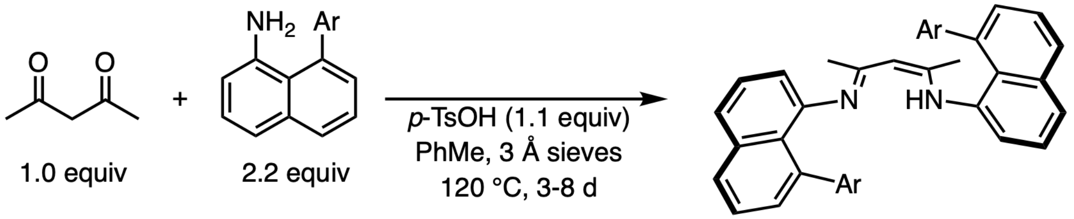

General procedure 2: 8-arylnaphthalen-1-amine (2.2 equiv), *p*-TsOH • H_2O (1.1 equiv), acetylacetone (1.0 equiv) and anhydrous PhMe were combined in a 3-neck round bottom flask equipped with 3 Å sieves in a nylon tea bag and reflux condenser under N_2. After stirring at 25 °C for 15 min, the reaction was heated to 120 °C for 5 to 7 d, monitoring by TLC for full consumption of aniline. The reaction was cooled to 25 °C, poured into $NaOH_{(aq)}$ (0.45 M) and vigorously stirred for 15 min. The biphasic mixture was transferred to a separatory funnel with EtOAc. The layers were separated and the aqueous layer was extracted with EtOAc (2 X). The combined organics were washed with brine, dried over $MgSO_4$, filtered and dried *in vacuo*. The resulting residue was purified by recrystallization and dried at 60 °C *in vacuo* before bringing into a glovebox for storage.

***rac*-$^{4\text{-}Me}$BDI*H:** Following general procedure 2, 8-(4-methylphenyl)-naphthalen-1-amine (2.00 g, 8.57 mmol, 2.20 equiv), *p*-TsOH • H_2O (0.858 g, 4.51 mmol, 1.16 equiv), acetylacetone (0.40 mL, 3.9 mmol, 1.0 equiv) and anhydrous toluene (30 mL) were combined, stirred at 25 °C for 15 mins and then refluxed at 120 °C for 3 d. The reaction was cooled to 25 °C, poured into $NaOH_{(aq)}$ (70 mL, 0.45 M) and vigorously stirred for 15 min. After workup, the residue was recrystallized from 1:2 PhMe:EtOH at 5 °C to yield the title compound as a yellow crystalline solid (1.30 g, 63%). **^{1}H NMR** (500 MHz, $CDCl_3$) δ 10.67 (bs, 1H), 7.79 (dd, *J* = 8.2, 1.4 Hz, 2H), 7.65 (dd, *J* = 8.1, 1.3 Hz, 2H), 7.48 (dd, *J* = 8.1, 7.3 Hz, 2H), 7.37 (dd, *J* = 8.1, 7.1 Hz, 2H), 7.13 (dd, *J* = 7.0, 1.4 Hz, 2H), 7.01 – 6.93 (m, 4H), 6.93 – 6.87 (m, 4H), 6.84 (dd, *J* = 7.3, 1.3 Hz, 2H), 3.98 (s, 1H), 2.35 (s, 6H), 1.54 (s, 6H). **^{13}C{^{1}H} NMR** (126 MHz, $CDCl_3$) δ 157.17, 142.86, 141.13, 139.89, 135.75, 134.60, 129.91, 128.71, 128.04, 127.97, 126.78, 125.39, 124.93, 124.50, 121.86, 98.08, 21.36, 20.92. **HRMS** (DART-Orbitrap) m/z calculated for $C_{39}H_{35}N_2$ $[M+H]^+$ 531.2800, found 531.2792

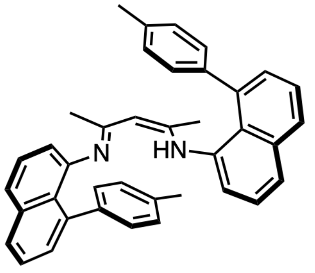

***rac*-$^{3,5\text{-}Me}$BDI*H:** Following general procedure 2, 8-(3,5-dimethylphenyl)-naphthalen-1-amine (1.50 g, 6.06 mmol, 2.20 equiv), *p*-TsOH • H_2O (0.58 g, 3.1 mmol, 1.1 equiv), acetylacetone (0.28 mL, 2.8 mmol, 1.0 equiv) and anhydrous toluene (21 mL) were combined, stirred at 25 °C for 15 mins and then refluxed at 120 °C for 3 d. The reaction was cooled to 25 °C, poured into $NaOH_{(aq)}$ (50 mL, 0.45 M) and vigorously stirred for 15 min. After workup, the residue was recrystallized from 1:2 PhMe:EtOH at 5 °C to yield the title compound as a yellow crystalline solid (0.58 g, 38%). **^{1}H NMR** (500 MHz, $CDCl_3$) δ 10.55 (s, 1H), 7.75 (dd, *J* = 8.2, 1.4 Hz, 2H), 7.61 (dd, *J* = 8.2, 1.4 Hz, 2H), 7.43 (t, *J* = 7.7 Hz, 2H), 7.34 (t, *J* = 7.6 Hz, 2H), 7.12 (dd, *J* = 7.0, 1.4 Hz, 2H), 6.84 (bs, 2H), 6.66 (dd, *J* = 7.3, 1.4 Hz, 2H), 6.62 (bs, 4H), 4.30 (s, 1H), 2.22 (s, 12H), 1.66 (s, 6H). **^{13}C{^{1}H} NMR** (126 MHz, $CDCl_3$) δ 157.19, 143.79, 142.70, 140.08, 136.32 (bs), 135.72, 130.11, 127.97, 127.39, 127.09 (bs), 126.89, 125.39, 124.86, 124.47, 122.88, 97.74, 21.56 (bs), 21.13. **HRMS** (DART-Orbitrap) m/z calculated for $C_{41}H_{39}N_2$ $[M+H]^+$ 559.3113, found 559.3108.

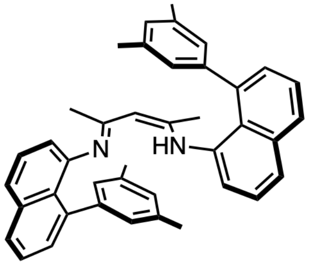

***rac*-$^{3,5\text{-F}}$BDI*H:** Following general procedure 2, 8-(3,5-difluorophenyl)-naphthalen-1-amine (1.04 g, 4.09 mmol, 2.15 equiv), *p*-TsOH • H_2O (0.39 g, 2.0 mmol, 1.1 equiv), acetylacetone (0.19 mL, 1.9 mmol, 1.0 equiv) and anhydrous toluene (21 mL) were combined, stirred at 25 °C for 15 mins and then refluxed at 120 °C for 8 d. The reaction was cooled to 25 °C, poured into $NaOH_{(aq)}$ (30 mL, 0.45 M) and vigorously stirred for 15 min. After workup, the residue was recrystallized from 1:2 PhMe:EtOH at 5 °C to yield the title compound as a yellow crystalline solid (0.43 g, 40%). **^{1}H NMR** (500 MHz, $CDCl_3$) δ 11.08 (s, 1H), 7.82 (dd, *J* = 8.2, 1.4 Hz, 2H), 7.66 (dd, *J* = 8.3, 1.3 Hz, 2H), 7.51 (t, *J* = 7.8 Hz, 2H), 7.36 (dd, *J* = 8.2, 7.0 Hz, 2H), 7.08 (dd, *J* = 7.0, 1.3 Hz, 2H), 6.93 (dd, *J* = 7.3, 1.3 Hz, 2H), 6.66 (tt, *J* = 9.2, 2.4 Hz, 2H), 6.54 (d, *J* = 9.1 Hz, 4H), 4.22 (s, 1H), 1.71 (s, 6H). **^{13}C{^{1}H} NMR** (126 MHz, $CDCl_3$) δ 162.11 (dd, *J* = 246.3, 13.2 Hz), 158.32, 147.63 (t, *J* = 9.9 Hz), 141.76, 137.34 (t, *J* = 2.3 Hz), 135.66, 129.55, 129.11, 126.56, 126.26, 124.81, 124.79, 122.97 (t, *J* = 2.3 Hz), 111.99 (d, *J* = 21.4 Hz), 100.99 (t, *J* = 25.4 Hz), 97.60, 20.79. **^{19}F NMR** (470 MHz, $CDCl_3$) δ -110.62 (bs), -112.75 (bs). **HRMS** (DART-Orbitrap) m/z calculated for $C_{37}H_{27}F_4N_2$ $[M+H]^+$ 575.2110, found 575.2101.

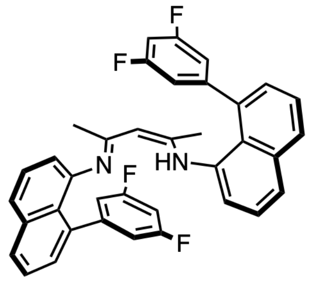

***rac*-$^{3,5\text{-Cl}}$BDI*H:** Following general procedure 2, 8-(3,5-dichlorophenyl)-naphthalen-1-amine (0.92 g, 3.2 mmol, 1.8 equiv), *p*-TsOH • H_2O (0.30 g, 1.6 mmol, 0.89 equiv), acetylacetone (0.18 mL, 1.8 mmol, 1.0 equiv) and anhydrous toluene (8 mL) were combined, stirred at 25 °C for 15 mins and then refluxed at 120 °C for 8 d. The reaction was cooled to 25 °C, poured into $NaOH_{(aq)}$ (24 mL, 0.45 M) and vigorously stirred for 15 min. After workup, the residue was recrystallized from 1:2 PhMe:EtOH at 5 °C to yield the title compound as a yellow crystalline solid (0.74 g, 72%). **^{1}H NMR (500 MHz, $CDCl_3$)** δ 10.93 (s, 1H), 7.80 (dd, *J* = 8.2, 1.4 Hz, 2H), 7.65 (dd, *J* = 8.2, 1.4 Hz, 2H), 7.53 (t, *J* = 7.8 Hz, 2H), 7.35 (dd, *J* = 8.2, 7.0 Hz, 2H), 7.23 (t, *J* = 1.9 Hz, 2H), 7.07 (dd, *J* = 7.0, 1.3 Hz, 2H), 6.95 (dd, *J* = 7.3, 1.3 Hz, 2H), 6.90 (bs, 4H), 4.44 (s, 1H), 1.79 (s, 6H). **^{13}C{^{1}H} NMR** (126 MHz, $CDCl_3$) δ 158.35, 147.10, 141.57, 136.79, 135.69, 133.68 (bs), 130.01, 129.19, 128.30 (bs), 126.60, 126.38, 125.66, 124.83, 124.80, 123.58, 98.04, 20.91. **HRMS** (DART-Orbitrap) m/z calculated for $C_{37}H_{27}Cl_4N_2$ $[M+H]^+$ 639.0928, found 639.0919.

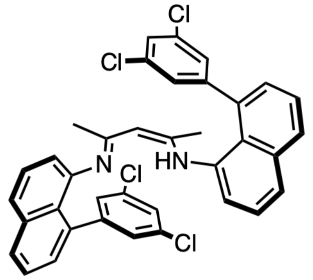

***rac*-$^{2\text{-naphthyl}}$BDI*H:** Following general procedure 2, [1,2'-binaphthalen]-8-amine (1.48 g, 5.48 mmol, 2.19 equiv), *p*-TsOH • H_2O (0.52 g, 2.7 mmol, 1.1 equiv), acetylacetone (0.26 mL, 2.5 mmol, 1.0 equiv) and anhydrous toluene (19 mL) were combined, stirred at 25 °C for 15 mins and then refluxed at 120 °C for 8 d. The reaction was cooled to 25 °C, poured into $NaOH_{(aq)}$ (45 mL, 0.45 M) and vigorously stirred for 15 min. After workup, the residue was recrystallized from 1:2 PhMe:EtOH at 5 °C to yield the title compound as a green crystalline solid (0.63 g, 42%). The title compound was isolated as a mixture of rotational isomers. See Figure S66 and Figure S67 for more details. **^{1}H NMR** (500 MHz, $CDCl_3$) δ 10.66 – 10.37 (bs,1H, N*H*), 7.97 – 5.62 (m, 26H, Ar*H*), 3.54 – 2.38 (3 x s, 0.74:0.02:0.23, 1H, CH_3CHCH_3), 1.20 (bs, 6H, CH_3CHCH_3). **^{13}C{^{1}H} NMR** (126 MHz, $CDCl_3$) δ 157.59, 142.42, 142.29, 142.06, 141.99, 139.63, 135.64, 133.37, 131.91, 130.22, 130.17, 129.19, 128.51, 128.44, 128.43, 128.40, 128.38, 128.36, 128.22, 127.36, 127.33, 127.29, 126.78, 126.16, 126.02, 125.96, 125.87, 125.75, 125.61, 125.60, 125.50, 125.27, 124.89, 124.48, 122.33, 98.02, 97.83, 20.49. **HRMS** (DART-Orbitrap) m/z calculated for $C_{45}H_{35}N_2$ $[M+H]^+$ 603.2800, found, 603.2794.

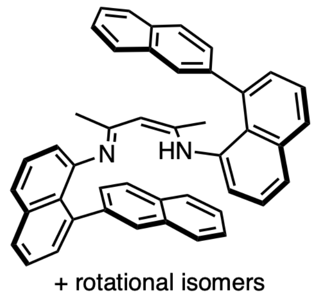

+ rotational isomers

***rac*-$^{3,5\text{-}CF3}$BDI*H:** Following general procedure 2, 8-(3,5-bis(trifluoromethyl)phenyl)-naphthalen-1-amine (4.18 g, 11.8 mmol, 2.23 equiv), *p*-TsOH • H_2O (1.1 g, 5.9 mmol, 1.1 equiv), acetylacetone (0.55 mL, 5.3 mmol, 1.0 equiv) and anhydrous toluene (40 mL) were combined, stirred at 25 °C for 15 mins and then refluxed at 120 °C for 8 d. The reaction was cooled to 25 °C, poured into $NaOH_{(aq)}$ (100 mL, 0.45 M) and vigorously stirred for 15 min. After workup, the residue was recrystallized from EtOH at 5 °C to yield the title compound as a yellow crystalline solid (0.73 g, 18%). **^{1}H NMR** (500 MHz, $CDCl_3$) δ 10.49 (s, 1H), 7.85 (dd, *J* = 8.2, 1.3 Hz, 2H), 7.74 (s, 2H), 7.68 (dd, *J* = 8.2, 1.3 Hz, 2H), 7.55 (bs, 2H), 7.48 (t, *J* = 7.8 Hz, 2H), 7.39 (dd, *J* = 8.2, 7.1 Hz, 2H), 7.33 (bs, *J* = 8.5 Hz, 2H), 7.07 (dd, *J* = 7.1, 1.3 Hz, 2H), 6.52 (dd, *J* = 7.3, 1.3 Hz, 2H), 4.40 (s, 1H), 1.70 (s, 6H). **^{13}C{^{1}H} NMR** (126 MHz, $CDCl_3$) δ 158.52, 146.15, 141.14, 136.27, 135.80, 131.18, 129.84, 128.68 (bq, *J* = 296.8 Hz), 126.36, 126.08, 125.34, 125.01, 124.81, 123.32, 122.64, 119.65 (hept, *J* = 7.4 Hz), 98.99, 20.96. **^{19}F NMR** (470 MHz, $CDCl_3$) δ -61.95, -62.36. **HRMS** (DART-Orbitrap) m/z calculated for $C_{41}H_{27}F_{12}N_2$ $[M+H]^+$ 775.1983, found 775.1971.

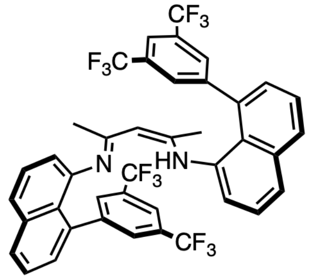

2.3 (^{Ar}BDI*)ZnO^{i}Pr Complex synthesis

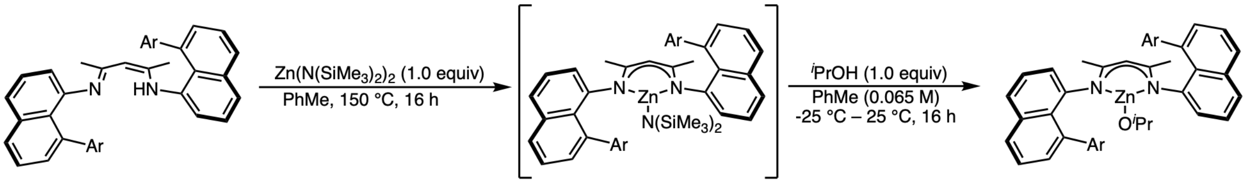

General procedure 3: In a glovebox, ^{Ar}BDI*H (1.0 equiv), $Zn(N(SiMe_3)_2)_2$ (1.0 equiv) and PhMe (1 drop per 25 mg of ^{Ar}BDI*H) were added to a scintillation vial with stir bar and heated to 150 °C for 16 h. The reaction was cooled to 25 °C, diluted with PhMe (0.065 M) and placed in a -25 °C freezer for 15 min. While stirring, ^{i}PrOH solution (10% v/v in PhMe, 1.0 equiv) was added dropwise and the reaction stirred at 25 °C for 16 h. Some of or all the volatiles (*vide infra* for compound specific conditions) were removed *in vacuo* and the complexes were recrystallized at -25 °C. The mother liquor was removed, and the crystals were dried *in vacuo* for no more than 4 h before being stored at -25 °C. *Note:* when drying complexes for extended periods (>4 h) *in vacuo* at 25 °C, complex decomposition was observed. As such, any residual mol% PhMe was calculated by ^{1}H NMR spectroscopy and factored into yield and molar mass data.

***rac*-($^{4\text{-}Me}$BDI*)ZnO^{i}Pr:** Following general procedure 3, *rac*-$^{4\text{-}Me}$BDI*H (0.200 g, 0.378 mmol, 1.00 equiv), $Zn(N(SiMe_3)_2)_2$ (153 µL, 0.378 mmol, 1.00 equiv) and PhMe (8 drops) were combined in an 8 mL vial and heated to 150 °C overnight. The reaction was cooled to 25 °C, diluted with PhMe (5.8 mL) and cooled further to -25 °C for 15 min. At 25 °C, ^{i}PrOH solution (0.26 mL, 0.38 mmol, 1.0 equiv, 10% v/v in PhMe) was added dropwise with stirring and was allowed to react at 25 °C for 16 h. The volatiles were removed *in vacuo* and the residue recrystallized from 1:1 PhMe:hexanes (5 mL total) to yield the title compound as a yellow crystalline solid containing 1 equiv PhMe (0.173 g, 61%). **^{1}H NMR** (500 MHz, C_6D_6) δ 7.65 (dd, *J* = 7.6, 2.0 Hz, 2H), 7.63 (dd, *J* = 7.6, 2.0 Hz, 2H), 7.58 (dd, *J* = 8.1, 1.3 Hz, 2H), 7.41 (t, *J* = 7.7 Hz, 2H), 7.30 (dd, *J* = 7.7, 2.0 Hz, 2H), 7.24 – 7.18 (m, 6H), 7.05 (dd, *J* = 7.7, 2.0 Hz, 2H), 6.94 – 6.87 (m, 2H), 3.94 (s, 1H), 3.80 (hept, *J* = 5.9 Hz, 1H), 2.26 (s, 6H), 1.29 (s, 6H), 0.83 (d, *J* = 5.9 Hz, 6H). **^{13}C{^{1}H} NMR** (126 MHz, C_6D_6) δ 166.75, 146.75, 142.76, 139.53, 136.27, 135.39, 130.76, 129.57, 129.31, 129.26, 129.01, 126.49, 125.77, 125.25, 123.48, 97.96, 66.56, 29.75, 23.70, 21.38.

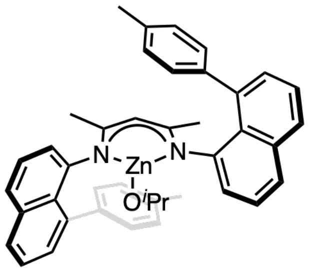

***rac*-($^{3,5\text{-}Me}$BDI*)ZnO^{i}Pr:** Following general procedure 3, *rac*-$^{3,5\text{-}Me}$BDI*H (0.0960 g, 0.172 mmol, 1.00 equiv), $Zn(N(SiMe_3)_2)_2$ (69 µL, 0.17 mmol, 1.0 equiv) and PhMe (4 drops) were combined in a 4 mL vial and heated to 150 °C overnight. The reaction was cooled to 25 °C, diluted with PhMe (2.6 mL) and cooled further to -25 °C for 15 min. At 25 °C, ^{i}PrOH solution (119 µL, 0.172 mmol, 1.00 equiv, 10% v/v in PhMe) was added dropwise with stirring and was allowed to react at 25 °C for 16 h. Half of the volatiles were removed *in vacuo* and the remaining liquid layered with hexanes (2 mL) to yield the title compound as a yellow crystalline solid (0.099 g, 84%). **^{1}H NMR** (500 MHz, C_6D_6) δ 7.62 (dd, *J* = 7.6, 1.9 Hz, 2H), 7.56 (dd, *J* = 8.2, 1.3 Hz, 2H), 7.37 (dd, *J* = 8.2, 7.3 Hz, 2H), 7.25 (d, *J* = 2.1 Hz, 2H), 7.22 – 7.17 (m, 4H), 7.03 (dd, *J* = 7.3, 1.4 Hz, 2H), 6.90 – 6.85 (m, 2H), 6.62 (s, 2H), 4.20 (s, 1H), 3.68 (hept, *J* = 5.9 Hz, 1H), 2.37 (s, 6H), 2.16 (s, 6H), 1.40 (s, 6H), 0.72 (d, *J* = 5.9 Hz, 6H). **^{13}C{^{1}H} NMR** (126 MHz, C_6D_6) δ 166.27, 146.51, 145.28, 142.13, 139.84, 137.77, 136.95, 136.14, 130.61, 128.91, 127.52, 127.46, 127.24, 126.47, 125.28, 125.14, 124.40, 96.48, 66.21, 29.33, 24.04, 22.01, 21.21.

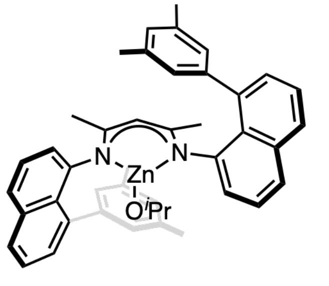

***rac*-($^{3,5\text{-}F}$BDI*)ZnO^{i}Pr:** Following general procedure 3, *rac*-$^{3,5\text{-}F}$BDI*H (0.0987 g, 0.172 mmol, 1.00 equiv), $Zn(N(SiMe_3)_2)_2$ (69 µL, 0.17 mmol, 1.0 equiv) and PhMe (4 drops) were combined in a 4 mL vial and heated to 150 °C overnight. The reaction was cooled to 25 °C, diluted with PhMe (2.6 mL) and cooled further to -25 °C for 15 min. At 25 °C, ^{i}PrOH solution (119 µL, 0.172 mmol, 1.00 equiv, 10% v/v in PhMe) was added dropwise with stirring and was allowed to react at 25 °C for 16 h. Half of the volatiles were removed *in vacuo* and the remaining liquid layered with hexanes (2 mL) to yield the title compound as a pale yellow crystalline solid (0.084 g, 70%). **^{1}H NMR** (500 MHz, C_6D_6) δ 7.57 (dd, *J* = 8.2, 1.4 Hz, 2H), 7.47 (dd, *J* = 8.2, 1.4 Hz, 2H), 7.39 (dd, *J* = 8.2, 7.3 Hz, 2H), 7.24 (dd, *J* = 7.4, 1.3 Hz, 2H), 7.15 – 7.11 (m, 2H), 7.08 (dd, *J* = 8.2, 7.0 Hz, 2H), 6.89 (dd, *J* = 7.0, 1.4 Hz, 2H), 6.52 – 6.37 (m, 4H), 4.13 (s, 1H), 3.55 (hept, *J* = 5.9 Hz, 1H), 1.33 (s, 6H), 0.65 (d, *J* = 5.9 Hz, 6H). **^{13}C{^{1}H} NMR** (126 MHz, C_6D_6) δ 167.93, 164.16 – 161.61 (2 x dd), 149.13 (t, *J* = 10.0 Hz), 145.35, 136.64, 136.04, 130.19, 129.95, 126.64, 126.39, 124.94, 124.66, 124.63, 112.30 (dt, *J* = 21.8, 2.8 Hz), 101.51 (t, *J* = 25.1 Hz), 97.32, 66.36, 29.54, 23.72. **^{19}F NMR** (470 MHz, C_6D_6) δ -107.61 (q, *J* = 8.4 Hz), -111.11 (q, *J* = 8.6 Hz).

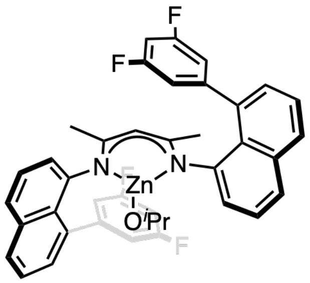

***rac*-($^{3,5\text{-}Cl}$BDI*)ZnO^{i}Pr:** Following general procedure 3, *rac*-$^{3,5\text{-}Cl}$BDI*H (0.0995 g, 0.155 mmol, 1.00 equiv), $Zn(N(SiMe_3)_2)_2$ (63 µL, 0.16 mmol, 1.0 equiv) and PhMe (4 drops) were combined in a 4 mL vial and heated to 150 °C overnight. The reaction was cooled to 25 °C, diluted with PhMe (2.4 mL) and cooled further to -25 °C for 15 min. At 25 °C, ^{i}PrOH solution (108 µL, 0.155 mmol, 1.00 equiv, 10% v/v in PhMe) was added dropwise with stirring and was allowed to react at 25 °C for 16 h. Half of the volatiles were removed *in vacuo* and the remaining liquid layered with hexanes (2 mL) to yield the title compound as a pale orange crystalline solid containing 0.2 equiv PhMe (0.097 g, 80%). **^{1}H NMR** (500 MHz, C_6D_6) δ 7.57 (dd, *J* = 8.3, 1.4 Hz, 2H), 7.49 (dd, *J* = 8.2, 1.3 Hz, 2H), 7.45 – 7.38 (m, 4H), 7.19 (dd, *J* = 7.2, 1.4 Hz, 2H), 7.11 (t, *J* = 2.0 Hz, 2H), 7.06 (dd, *J* = 8.2, 7.0 Hz, 2H), 6.79 (dd, *J* = 7.0, 1.3 Hz, 2H), 6.70 (t, *J* = 1.7 Hz, 2H), 4.45 (s, 1H), 3.49 (hept, *J* = 6.0 Hz, 1H), 1.43 (s, 6H), 0.62 (d, *J* = 6.0, 6H). **^{13}C{^{1}H} NMR** (126 MHz, C_6D_6) δ 167.55, 148.28, 145.07, 136.18, 136.00, 134.78, 134.68, 130.61, 129.96, 127.39, 127.14, 126.59, 126.28, 126.21, 125.40, 124.98, 97.65, 66.24, 29.38, 23.94.

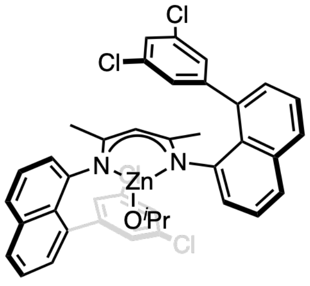

***rac*-($^{2\text{-naphthyl}}$BDI*)ZnO^{i}Pr):** Following general procedure 3, *rac*-$^{2\text{-naphthyl}}$BDI*H (0.0915 g, 0.152 mmol, 1.00 equiv), $Zn(N(SiMe_3)_2)_2$ (61 µL, 0.15 mmol, 1.0 equiv) and PhMe (4 drops) were combined in a 4 mL vial and heated to 150 °C overnight. The reaction was cooled to 25 °C, diluted with PhMe (2.3 mL) and cooled further to -25 °C for 15 min. At 25 °C, ^{i}PrOH solution (105 µL, 0.152 mmol, 1.00 equiv, 10% v/v in PhMe) was added dropwise with stirring and was allowed to react at 25 °C for 16 h. Half of the volatiles were removed *in vacuo* and the remaining liquid layered with hexanes (2 mL) to yield the title compound as a pale brown crystalline solid containing 0.3 equiv PhMe (0.096 g, 84%). The title compound was isolated as a mixture of rotational isomers. See Figure S80 and Figure S81 for more details. **^{1}H NMR** (500 MHz, C_6D_6) δ 8.15 – 5.00 (m, 26H, Ar*H*), 4.09 – 3.11 (3 x s, 0.17: 0.56:0.30, 1H, CH_3CHCH_3), 3.81 (bs, 1H, $OCH(CH_3)_2$), 1.32 – 0.68 (m, 12H, CH_3CHCH_3 and $OCH(CH_3)_2$. **^{13}C{^{1}H} NMR** (126 MHz, C_6D_6) δ 167.62, 167.57, 166.30, 165.98, 146.56, 146.33, 145.75, 145.48, 143.87, 143.75, 142.99, 142.66, 139.33, 139.25, 139.15, 139.12, 139.03, 136.33, 136.14, 135.87, 134.46, 134.32, 133.68, 133.66, 132.58, 132.50, 132.34, 132.30, 131.30, 131.04, 130.61, 130.56, 129.64, 129.51, 129.40, 129.34, 129.27, 129.16, 129.00, 128.66, 128.57, 127.78, 127.75, 127.67, 127.64, 127.53, 127.29, 127.22, 126.95, 126.92, 126.84, 126.50, 126.43, 126.39, 126.35, 126.33, 126.26, 126.14, 126.12, 126.08, 126.06, 126.01, 125.93, 125.64, 125.62, 125.55, 125.33, 125.15, 124.97, 124.88, 124.21, 124.14, 123.61, 98.16, 97.76, 97.67, 66.60, 31.97, 29.86, 23.76, 23.57, 23.40, 23.06, 14.36.

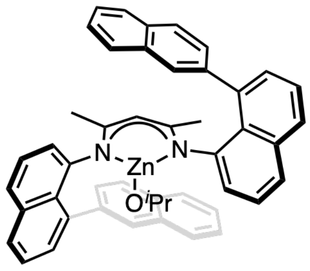

+ rotational isomers

***rac*-($^{3,5\text{-}CF_3}$BDI*)ZnO^{i}Pr:** Following general procedure 3, *rac*-$^{3,5\text{-}CF_3}$BDI*H (0.102 g, 0.132 mmol, 1.00 equiv), $Zn(N(SiMe_3)_2)_2$ (53 µL, 0.13 mmol, 1.0 equiv) and PhMe (4 drops) were combined in a 4 mL vial and heated to 150 °C overnight. The reaction was cooled to 25 °C, diluted with PhMe (2.0 mL) and cooled further to -25 °C for 15 min. At 25 °C, ^{i}PrOH solution (91 µL, 0.13 mmol, 1.0 equiv, 10% v/v in PhMe) was added dropwise with stirring and was allowed to react at 25 °C for 16 h. The volatiles were removed *in vacuo* and the residue recrystallized from hexanes (2 mL) to yield the title compound as a brown-green crystalline solid (0.070 g, 59%). **^{1}H NMR** (500 MHz, C_6D_6) δ 7.92 (s, 2H), 7.76 (s, 2H), 7.55 (dd, *J* = 8.3, 1.3 Hz, 2H), 7.46 (dd, *J* = 8.3, 1.3 Hz, 2H), 7.37 (t, *J* = 7.7 Hz, 2H), 7.09 (s, 2H), 7.02 (dd, *J* = 8.2, 7.1 Hz, 2H), 6.66 (d, *J* = 7.3 Hz, 2H), 6.62 (dd, *J* = 7.2, 1.4 Hz, 2H), 4.35 (s, 1H), 3.29 (hept, *J* = 5.9 Hz, 1H), 1.42 (s, 6H), 0.42 (d, *J* = 5.9 Hz, 6H). **^{13}C{^{1}H} NMR** (126 MHz, C_6D_6) δ 167.20, 147.33, 144.35, 136.07, 135.61, 131.71 (q, *J* = 32.7 Hz), 131.71, 130.47, 130.32 (q, *J* = 33.0 Hz), 129.82 (q, *J* = 5.1 Hz), 128.71 (q, *J* = 4.3 Hz), 126.91, 126.51, 126.08, 125.24, 125.14, 124.04 (q, *J* = 268.0 Hz), 123.96 (q, *J* = 273.2 Hz), 119.87 (hept, *J* = 3.5 Hz), 98.87, 66.20, 29.04, 23.92. **^{19}F NMR** (470 MHz, C_6D_6) δ -61.58, -62.15.

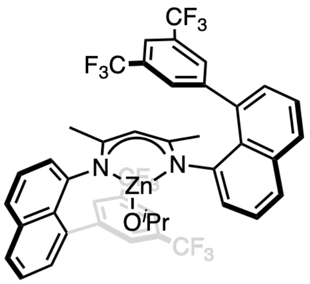

2.4 Synthesis of [(^{iPr}BDI)ZnO^{i}Pr]$_2$

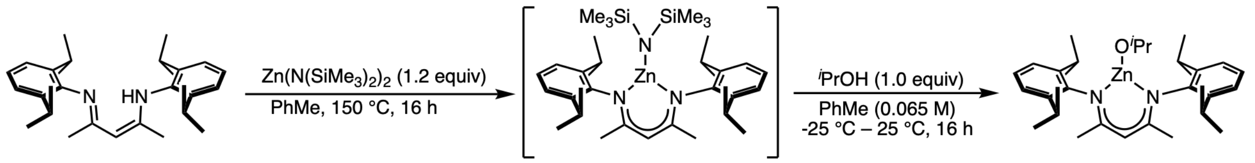

[^{iPr}BDIZn(O^{i}Pr)]$_2$: In a glovebox, ^{iPr}BDIH[12] (0.199 g, 0.475 mmol, 1.00 equiv), Zn(N(SiMe$_3$)$_2$)$_2$ (0.23 mL, 0.57 mmol, 1.2 equiv) and PhMe (8 drops) were added to an 8 mL vial with stir bar and heated to 150 °C for 16 h. Then, the volatiles were removed *in vacuo* at 150 °C for 7 h. The reaction was cooled to 25 °C, diluted and transferred to a 20 mL vial with PhMe (6.9 mL). The solution was cooled to -25 °C for 15 min. Next, ^{i}PrOH solution (364 µL, 0.475 mmol, 1.00 equiv, 10% v/v in PhMe) was added dropwise with stirring at 25 °C, and the reaction was left stirring for 16 h. Half of the solvent was removed *in vacuo* before the solution was chilled to -25 °C for 3 days. The mother liquor was removed and the solids dried *in vacuo* to a minimum to yield the title compound as a white crystalline solid containing 0.72 equiv PhMe (0.172 g, 52%). **^{1}H NMR** (500 MHz, C_6D_6) δ 7.10 (s, 6H), 4.92 (s, 1H), 3.85 (hept, *J* = 5.9 Hz, 1H), 3.18 (hept, *J* = 7.0 Hz, 4H), 1.65 (s, 6H), 1.40 (d, *J* = 6.8 Hz, 12H), 1.17 (d, *J* = 6.9 Hz, 12H), 0.92 (d, *J* = 6.0 Hz, 6H). **^{13}C{^{1}H} NMR** (126 MHz, C_6D_6) δ 169.57, 143.84, 141.72, 126.49, 123.97, 94.94, 66.49, 29.33, 28.69, 24.44, 23.52, 23.41. Chemical shift data is in agreement with literature.[13]

2.5 Synthesis of enantioenriched (*R*,*R*)-*trans*-DMPL

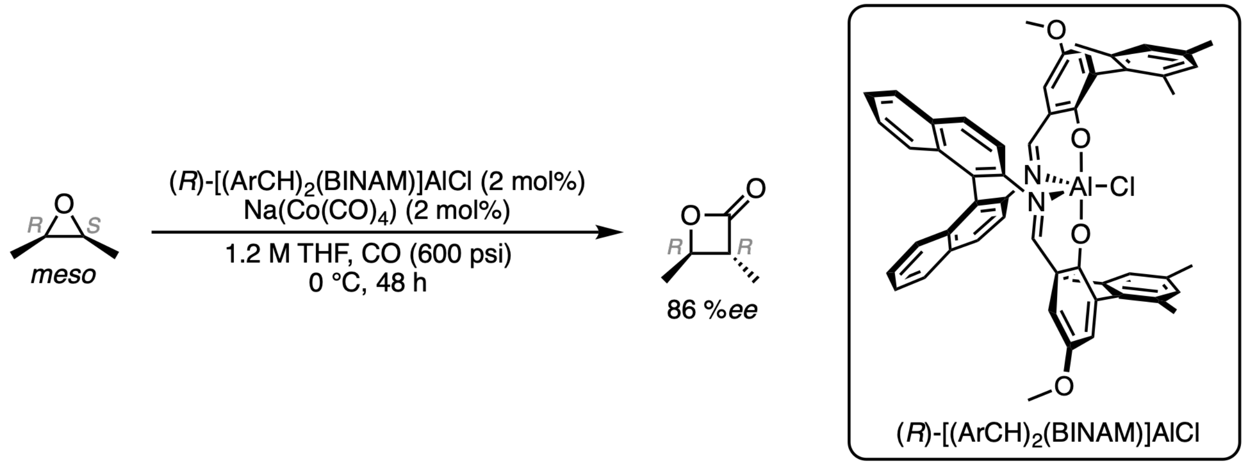

Enantioenriched (*R,R*)-*trans*-2,3-dimethylpropiolactone (DMPL): A pre-chilled (15 min at -25 °C) solution of *cis*-2-buteneoxide (3.39 g, 47.1 mmol, 51.0 equiv) in THF (30 min) was added to a pre-chilled (15 min at -25 °C) solution of $[(ArCH)_2(BINAM)]AlCl$[2] (790 mg, 0.929 mmol, 1.00 equiv) and $Na(Co(CO)_4)$ (182 mg, 0.947 mmol, 1.02 equiv) in THF (10 mL) in a pre-chilled Parr reactor (45 min at -25 °C). The reactor was sealed, brought out of the glovebox and charged and vented with 600 psi CO three times. The reaction was stirred at 0 °C for 48 h before being vented. The THF was removed *in vacuo*, before the lactone was distilled away from the catalyst residue. The resulting orange-red liquid was stirred over decolorizing carbon and CaH_2 for 3 days. The monomer was vacuum transferred to yield the title compound as a colorless oil with 0.05 equiv THF (1.56 g, 33%). The monomer was brought into a glovebox and stored over alumina beads at -25 °C for 24 h before use. The monomer was found to have an 86 %*ee* by chiral GC analysis. NMR chemical shift data is in agreement with literature.[3]

2.6 General polymerization procedure

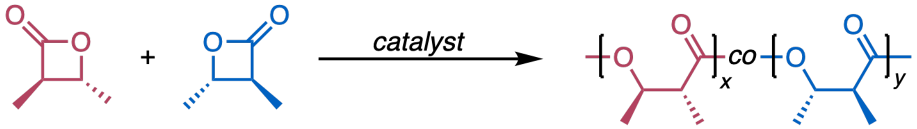

trans-DMPL was added to a solution of catalyst in solvent or solid catalyst (for neat polymerizations) in a vial with stir bar. For polymerization using CTA, ^{i}PrOH as a solution in PhMe was then added. The vial was capped and stirred at the appropriate temperature. Once polymerization time concluded, the vial was brought out of the glovebox, quenched with benchtop CH_2Cl_2, and homogenized with a vortex and gentle heat when necessary. An aliquot was taken for ^{1}H NMR spectroscopic analysis to determine conversion and for SEC analysis to determine crude molar mass data.

Precipitation procedure:

For highly isotactic samples (*mm*% ≥ 65%), polymer was precipitated from a concentrated (~100 mg/mL) solution in CH_2Cl_2 with cold MeOH (7 mL, -20 °C) in vials or culture tubes. The polymer suspensions were placed at -20 °C for 15 min before being centrifuged. The supernatant was gently pipetted off and the polymer dried *in vacuo*. The polymer was then redissolved in a minimal amount of CH_2Cl_2 and precipitated with cold (-20 °C) hexanes. The polymer suspensions were placed at -20 °C for 15 min before being centrifuged. The supernatant was gently pipetted off and the polymer dried *in vacuo* for 16 h before being subjected to DSC, ^{13}C{^{1}H} NMR spectroscopy, SEC and uniaxial tensile testing.

For less isotactic samples and the enantioenriched polymers used to determine stereoerrors, the polymers were purified using the above procedure except using cold hexanes for both precipitations.

2.7 Synthesis of syndioenriched *trans*-PHMB

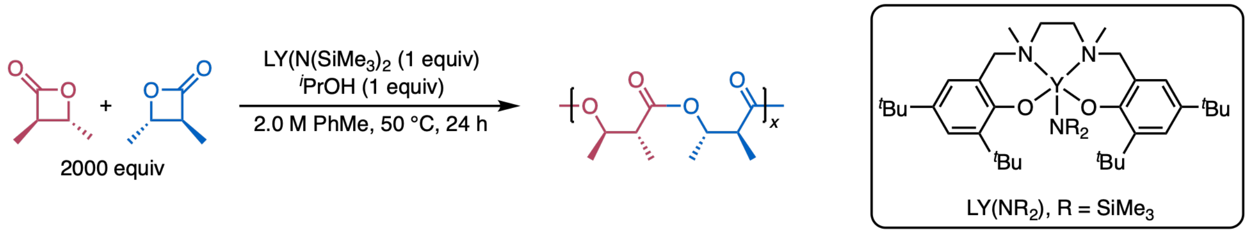

A solution of of ^{i}PrOH (60 μL, 6.0 μmol, 0.10 M in PhMe) and PhMe (4.70 mL) was added to a solution of precatalyst $LY(N(SiMe_3)_2)$[4] (4.5 mg, 0.0058 mmol) in PhMe (60 μL). Next, *rac-trans* DMPL (1.20 mL, 12.0 mmol) was added and the polymerization stirred at 50 °C for 24 h. To quench the polymerization, benzoic acid solution (180 μL, 0.018 mmol, 0.10 M) was added. Then, the polymer was precipitated into MeOH (400 mL), stirred for 30 min and filtered. The resulting white powder was dried *in vacuo* at 40 °C overnight.

3 Experimental details and polymer characterization

3.1 Determination of stereoerrors in $^{13}C\{^1H\}$ NMR

To help to decipher the stereosequence identity of the peaks in the $^{13}C\{^1H\}$ NMR spectra, a series of isotactic trans-PHMB polymers were prepared with specific tacticities. These polymers were made by doping in rac-trans-DMPL to enantioenriched (e.r.) (R,R)-trans-DMPL and polymerizing with a nonselective catalyst, [^{iPr}BDIZn(O^{i}Pr)]$_2$ (mm% = 20, see section 3.5).

Table S1: Polymer samples with targeted tacticity to determine $^{13}C\{^1H\}$ NMR signals

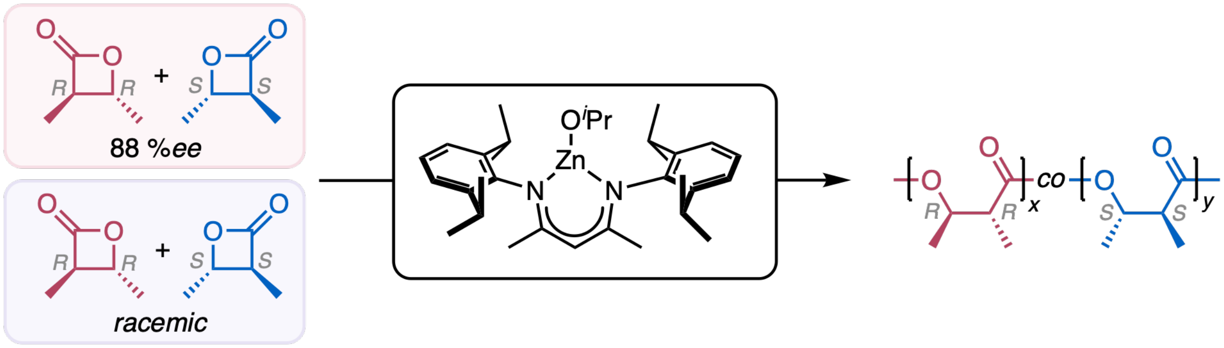

	Amount (µL)		Amount (mol%)							
Sample	88 %ee (*R*,*R*)-*trans*-DMPL	*rac*-*trans*-DMPL	(*R*,*R*)-*trans*-DMPL	(*S*,*S*)-*trans*-DMPL	*mm*% predicted[a]	Conv. (%)[b]	$M_{n, theo}$ (kDa)	$M_{n, SEC}$ (kDa)[c]	*Đ*[c]	T_m (°C)[d]
A	50	0	94	6	83	99	9.9	11.0	1.18	181/163
B	44	6	89	11	71	99	9.9	12.2	1.05	141
C	39	11	84	16	60	99	9.9	11.3	1.04	n/a

[a]Calculated using Equation S1. [b]Determined by ^{1}H NMR spectroscopic analysis comparing the relative integration of polymer and residual monomer. [c]Determined by SEC in THF at 30 °C, calibrated relative to monodisperse polystyrene standards. [d]Peak T_m values determined by DSC, polymorph 1/ polymorph 2.

Since [^{iPr}BDIZn(O^{i}Pr)]$_2$ is a nonselective catalyst, the probability of a meso linkage is simply the sum of the probability of three sequential (R,R)-trans-DMPL additions and three sequential (S,S)-trans-DMPL additions (i.e. a mock enantiomorphic site control mechanism).

Equation S1. Determination of the theoretical *m*% for polymerizations of enantioenriched *trans*-DMPL with a random catalyst

$$mm\% = [(mol\%\ R\ monomer)^3 + (mol\%\ S\ monomer)^3] * 100$$

Example: For sample C,

$$60\ mm\% = [(0.84)^3 + (0.16)^3]\bullet 100$$

Sample C was subjected to band-selective HSQCAD experiments to determine the order of stereoerrors being observed (dyad, triad, tetrad etc.). We observed the best resolution in the ^{13}C dimension for the carbon resonance at ca. 44.50 ppm (carbon α to the carbonyl)

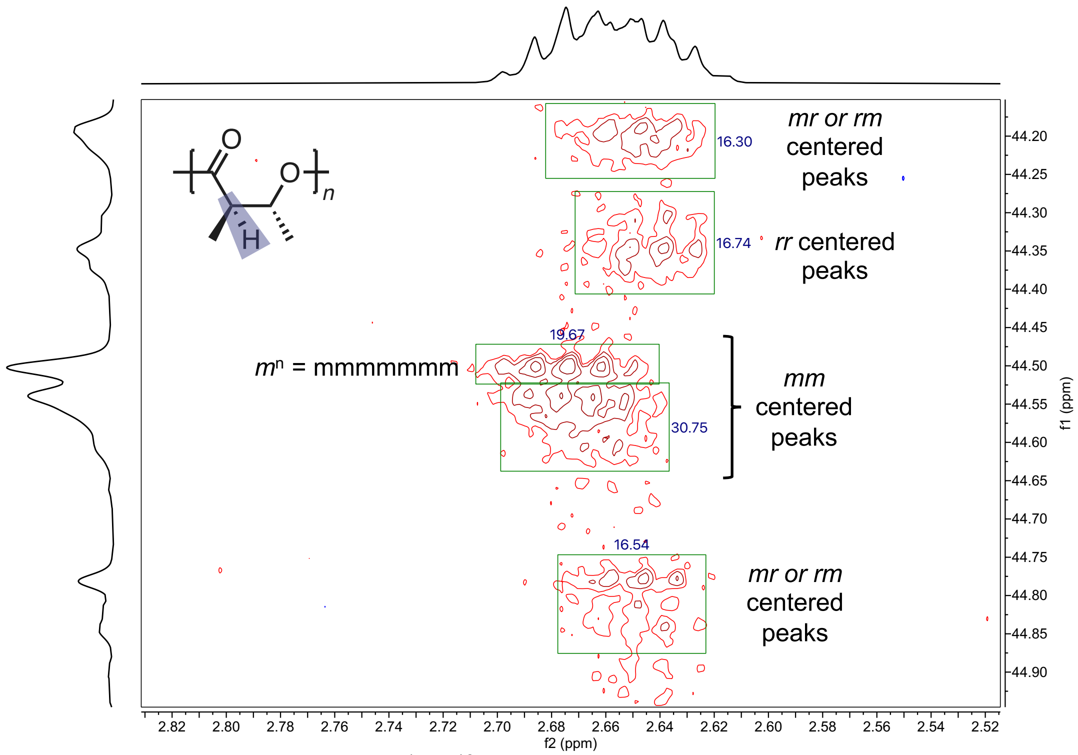

Figure S1: Band-selective HSQCAD (^{1}H, ^{13}C, 600/151 MHz, $CDCl_3$) for sample C in Table S1. *Vide infra* for peak assignments. Blue numbers are integrals.

The band-selective HSQCAD revealed many ^{13}C signals split into 4 groups of signals, suggesting a general triad resolution. The smaller peaks within each grouping must represent higher order errors centered about the triad. The most intense peak in the spectrum in Figure S1 *must be the mm centered peaks (ca. 44.55 ppm) due to the enantioenriched feedstock. A sample of syndiotactic trans-PHMB was synthesized (rr%= 66, Figure S2, see section 3.5 for synthetic details) revealing the peaks around 44.35 ppm are the rr centered peaks. By process of elimination, the groupings of peaks around 44.80 ppm and 44.20 ppm must be the mr and rm triad centered peaks.*

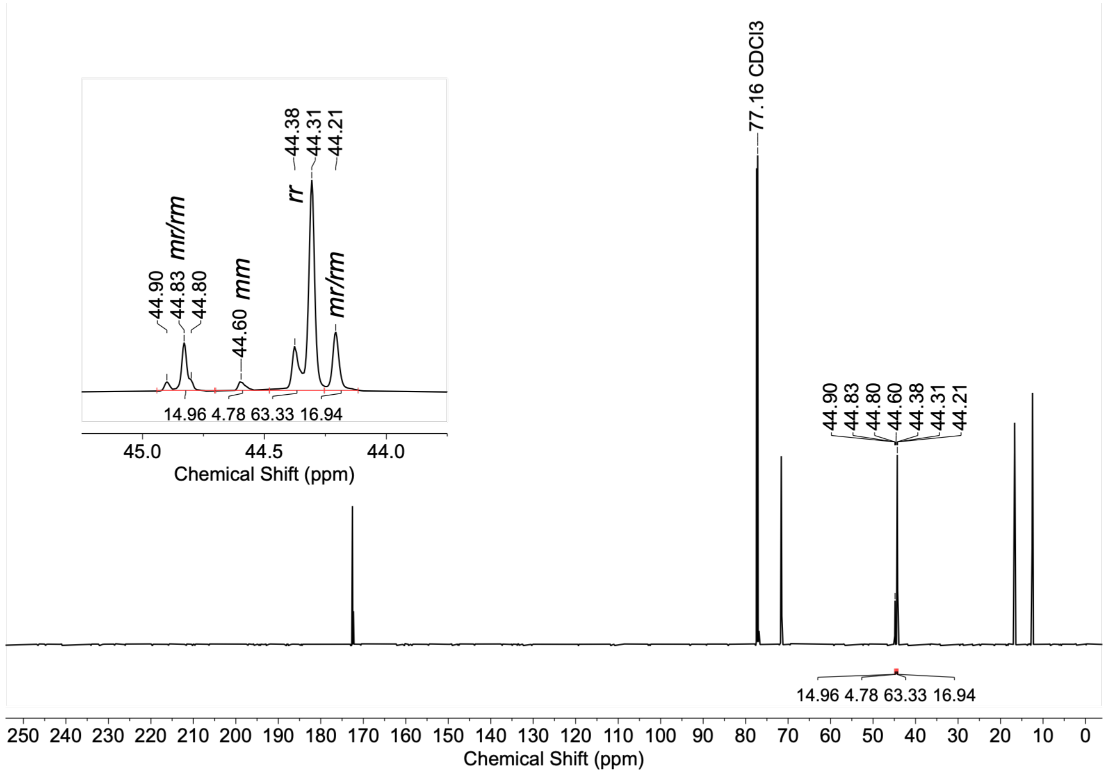

Figure S2: $^{13}C\{^1H\}$ NMR (126 MHz, $CDCl_3$) spectrum of syndioenriched *trans*-PHMB. See section 3.5 for polymer characterization details.

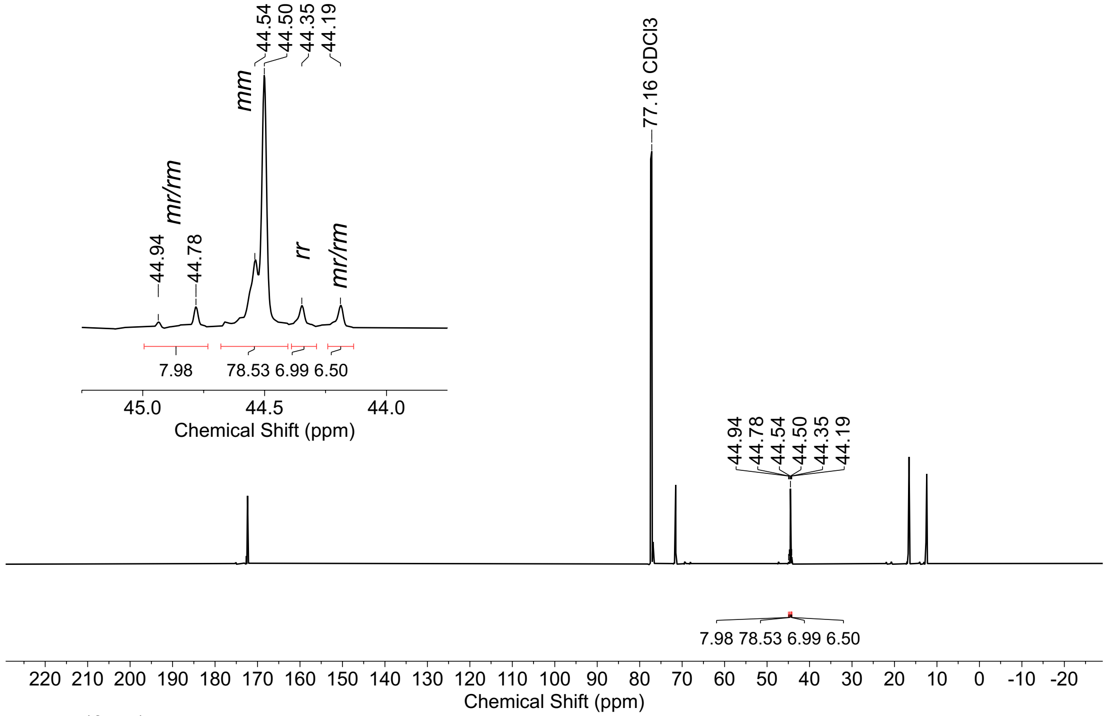

Figure S3: $^{13}C\{^1H\}$ NMR (126 MHz, $CDCl_3$) spectrum of sample A in Table S1.

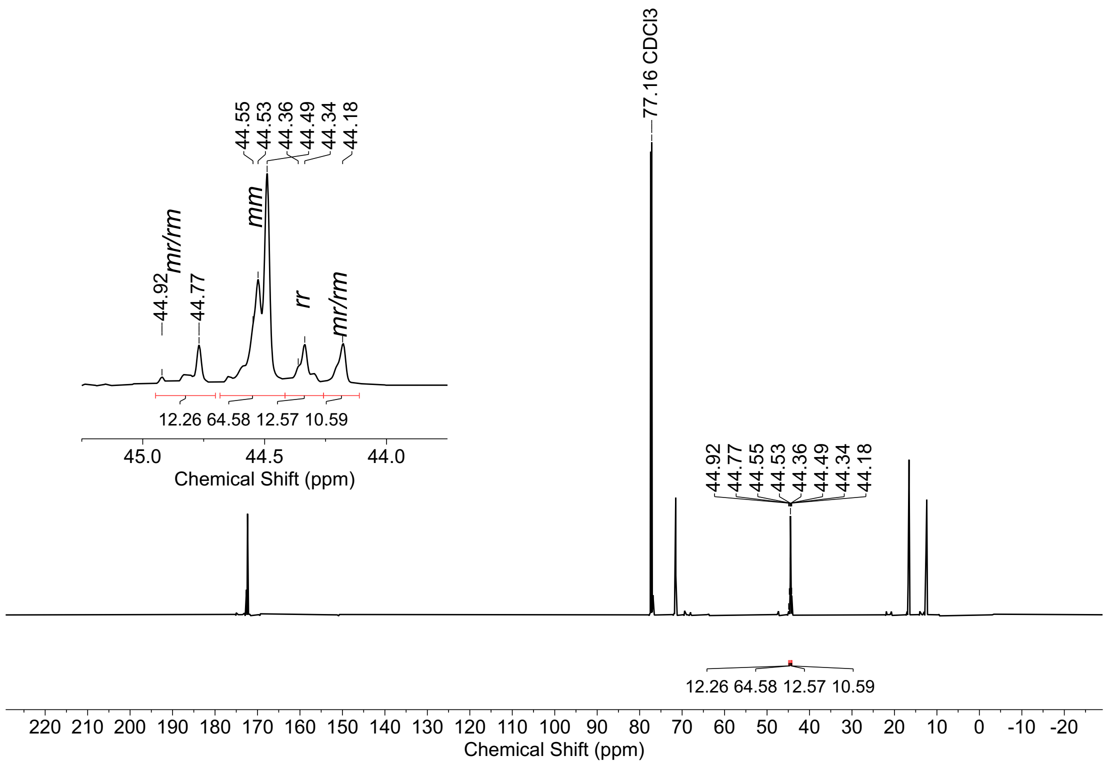

Figure S4: $^{13}C\{^1H\}$ NMR (126 MHz, $CDCl_3$) spectrum of sample B in Table S1.

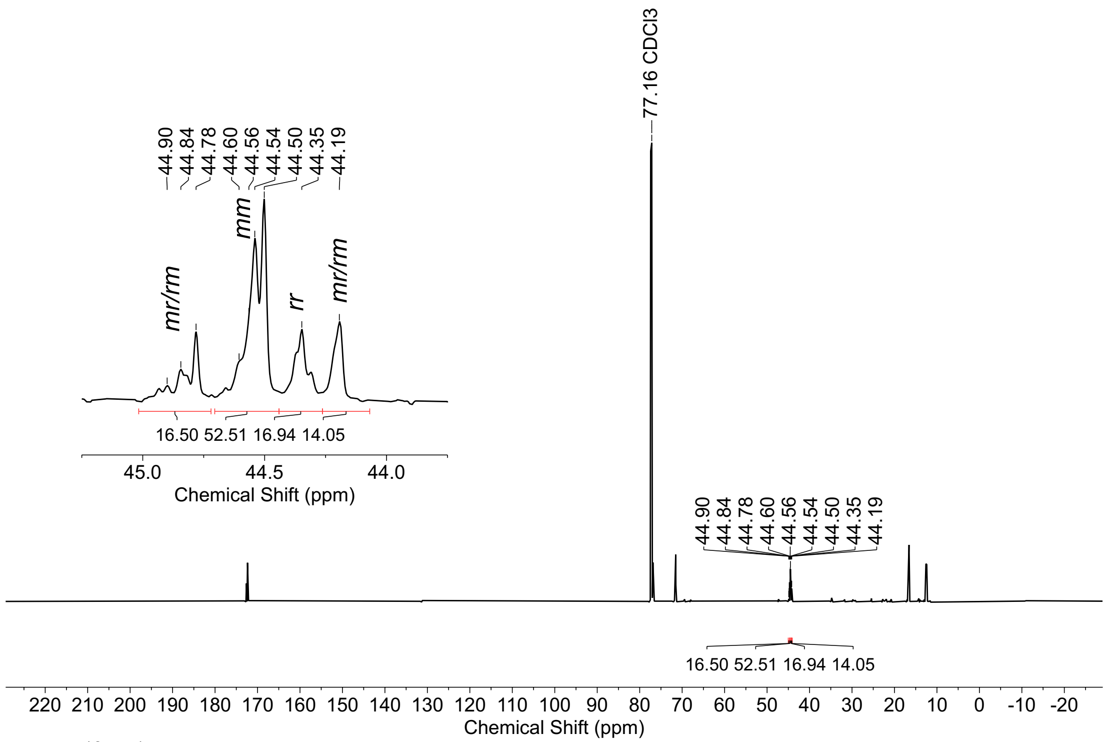

Figure S5: $^{13}C\{^1H\}$ NMR (126 MHz, $CDCl_3$) spectrum of sample C in Table S1.

Equation S2: Determination of *mm%* from a $^{13}C\{^1H\}$ NMR spectrum

$$mm\% = \frac{(mm)}{(mm) + (rr) + (mr) + (rm)} * 100$$

If α is defined as the probability of inserting an (R,R)-trans-DMPL monomer, then

Equation S3: Relationship between α and *mm*%

$$mm\% = \alpha^3 + (1 - \alpha)^3$$

Table S2: $^{13}C\{^1H\}$ NMR integration data for samples A, B and C.

	NMR integrations							
**Sample**	***mr/rm* (44.20 ppm)**	***rr* (44.35 ppm)**	***mm* (44.55 ppm)**	***mr/rm* (44.80 ppm)**	***mm*%^{a}**	**Predicted^b *mm*%**	**α^b**	**Predicted α^c**
A	6.50	6.99	78.53	7.98	78	83	0.92	0.94
B	10.59	12.57	64.58	12.26	65	71	0.87	0.89
C	14.05	16.94	52.51	16.50	53	60	0.81	0.84

[a]Calculated using Equation S2. [b]Calculated using Equation S3. [c]Equivalent to mol% (*R,R*)-*trans*-DMPL monomer added due to a non-selective catalyst used.

To determine the order of stereoerror observed by the tallest mm centered peak (Figure S1), we calculated the probabilities of m^n for sample C using our experimental α.

Table S3: Probability of each m^n peak for sample C.

Order	Signal Meaning	Probability Expression	*m*^{n}% Anticipated
dyad	*m*	$(0.81)^2 + (0.19)^2$	69
triad	*mm*	$(0.81)^3 + (0.19)^3$	54
tetrad	*mmm*	$(0.81)^4 + (0.19)^4$	43
pentad	*mmmm*	$(0.81)^5 + (0.19)^5$	35
hexad	*mmmmm*	$(0.81)^6 + (0.19)^6$	28
heptad	*mmmmmm*	$(0.81)^7 + (0.19)^7$	23
octad	*mmmmmmm*	$(0.81)^8 + (0.19)^8$	19
nonad	*mmmmmmmm*	$(0.81)^9 + (0.19)^9$	15

Since the m^n peak we observe constitutes ~20% of the total area (Figure S1), we hypothesize we are observing an mmmmmmm peak, signifying octad resolution.

3.2 Example tacticity calculation

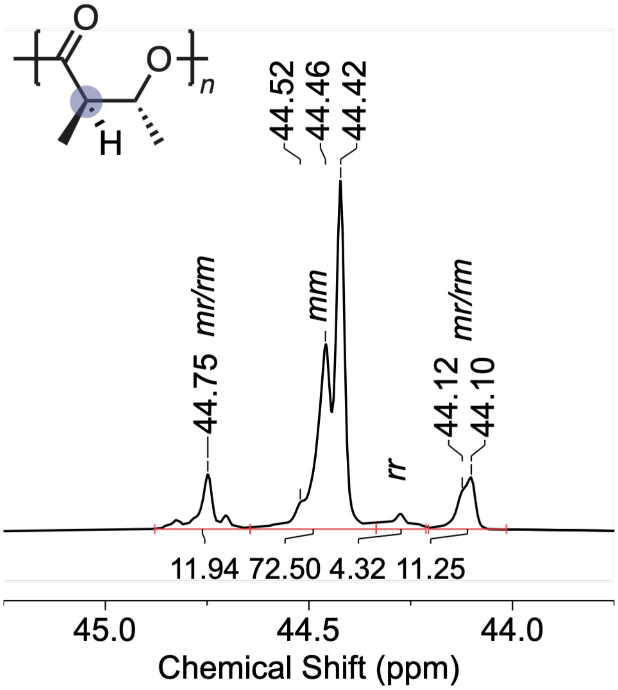

Figure S6: Portion of the $^{13}C\{^1H\}$ NMR (126 MHz, $CDCl_3$) spectrum of isoenriched *trans*-PHMB from Table 1, entry 2.

Using **Equation S2**,

$$mm\% = \frac{(72.50)}{(72.50) + (11.94) + (11.25) + (4.32)} * 100 = 73\%$$

Equation S4: Bernoulli triad test for chain-end control. If B ≅ 1, then system operates by a chain-end control mechanism of stereocontrol.

$$B = \frac{4(mm)(rr)}{[(rm) + (mr)]^2}$$

For the sample made by ($^{4\text{-Me}}$BDI*)ZnO^{i}Pr in Figure S6,

$$B = \frac{4(72.50)(4.32)}{[(11.94) + (11.25)]^2} = 2.33$$

Since B is close to 1, the mechanism of stereocontrol is dominated by a chain-end control model. Future work is focusing on the interplay of chain-end and enantiomorphic site control in this system.

3.3 Optimization of polymerization conditions

Table S4: Polymerization optimization for *rac*-($^{4\text{-Me}}$BDI*)ZnO^{i}Pr.

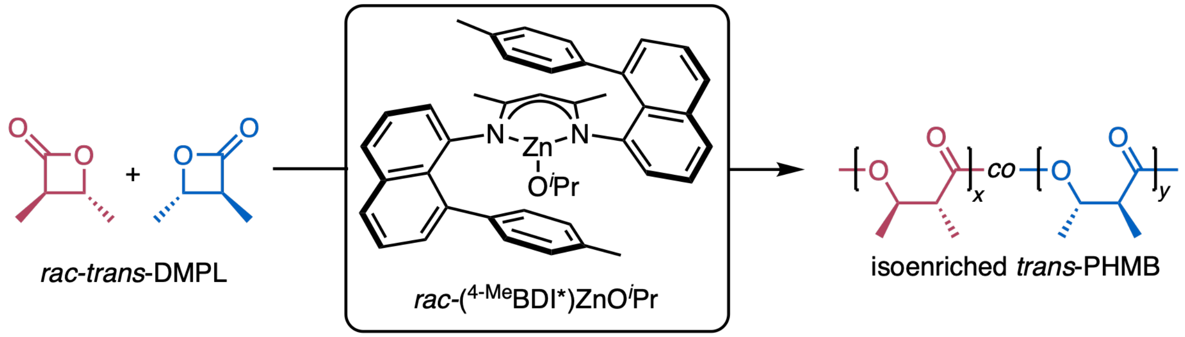

Entry[a]	solvent	conc. [DMPL]	T_{rxn} (°C)	Conv. (%)[b]	$M_{n,theo}$ (kDa)	$M_{n,SEC}$ (kDa)[c]	*Đ*[c]	T_m (°C)[d]	*mm*%[e]
1	none	–	0	77	15.4	20.5	1.54	166/145	74
2	CH_2Cl_2	4.0	0	69	13.8	16.1	1.04	159/135	74
3	CH_2Cl_2	2.5	0	42	8.4	10.3	1.04	173/158/140	74
4	PhMe	4.0	0	97	19.4	27.3	1.14	170/150	77
5	PhMe	2.5	0	91	18.2	26.0	1.09	169/149	77
6	none	–	25	98	19.6	24.5	1.13	157/135	68
7	CH_2Cl_2	4.0	25	>99	20.0	22.1	1.05	151/134	69
8	CH_2Cl_2	2.5	25	95	19.0	20.8	1.05	150/133	69
9	THF	4.0	25	>99	20.0	22.0	1.06	152/131	72
10	PhMe	4.0	25	>99	20.0	27.4	1.06	155/135	73
11	PhMe	2.5	25	>99	20.0	28.3	1.07	154/134	72

[a][*rac-trans*-DMPL]$_0$:[*rac*-$^{4\text{-Me}}$BDI*Zn(O^{i}Pr)]$_0$ = 200:1, t_{rxn} = 18 h. [b]Determined by ^{1}H NMR spectroscopic analysis comparing the relative integration of polymer and residual monomer. [c]Determined by SEC in THF at 30 °C, calibrated relative to monodisperse polystyrene standards. [d]Determined by DSC, polymorph 1/polymorph 2. [e]Determined by ^{13}C{^{1}H} NMR spectroscopic analysis using the carbon α to the carbonyl.

3.4 Comparison of [(^{iPr}BDI)ZnO^{i}Pr]$_2$ and ($^{4\text{-Me}}$BDI*)ZnO^{i}Pr rates

Table S5: Comparison of [(^{iPr}BDI)ZnO^{i}Pr]$_2$ and ($^{4\text{-Me}}$BDI*)ZnO^{i}Pr rates.

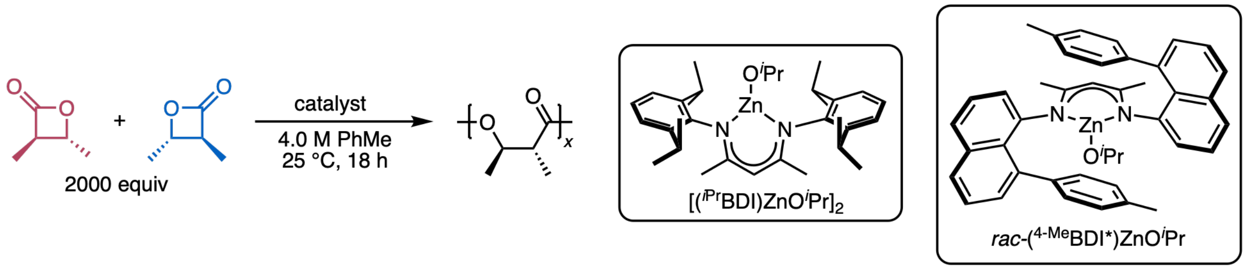

Entry[a]	catalyst	Conv. (%)[b]	$M_{n,theo}$ (kDa)	$M_{n,SEC}$ (kDa)[c]	*Đ*[c]
1	[(^{iPr}BDI)ZnO^{i}Pr]$_2$	13	26.0	21.1	1.03
2	($^{4\text{-Me}}$BDI*)ZnO^{i}Pr	96	192.2	185.9	1.04

[a][*rac-trans*-DMPL]$_0$:[catalyst]$_0$ = 2000:1, t_{rxn} = 18 h. [b]Determined by ^{1}H NMR spectroscopic analysis comparing the relative integration of polymer and residual monomer. [c]Determined by SEC in THF at 30 °C, calibrated relative to monodisperse polystyrene standards.

3.5 Synthesis of atactic and syndioenriched *trans*-PHMB

Polymerizations to synthesize atactic and enantioenriched trans-PHMB were set up following the general polymerization procedure in section 2.6.

Table S6: Polymer characterization data for polymerizations using $[(^{iPr}BDI)ZnO^{i}Pr]_2$.

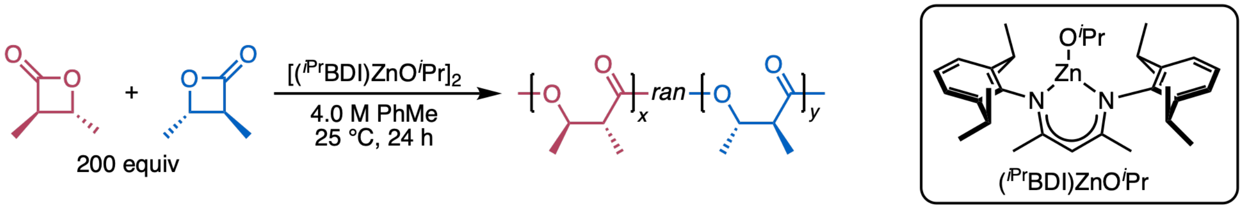

Entry[a]	monomer	Conv. (%)[b]	$M_{n,theo}$ (kDa)	$M_{n,SEC}$ (kDa)[c]	$Đ$[c]	T_m (°C)[d]	*mm*%[e]
1	*racemic*	97	19.4	21.8	1.03	N/A	20
2	86 %*ee* (*R*,*R*)	97	19.4	18.2	1.11	154	73

[a] $[rac\text{-}trans\text{-DMPL}]_0:[(^{iPr}BDI)ZnO^{i}Pr]_0$ = 200:1, t_{rxn} = 24 h. [b] Determined by ^1H NMR spectroscopic analysis comparing the relative integration of polymer and residual monomer. [c] Determined by SEC in THF at 30 °C, calibrated relative to monodisperse polystyrene standards. [d] Determined by DSC. [e] Determined by $^{13}C\{^1H\}$ NMR spectroscopic analysis using the carbon α to the carbonyl.

For entry 2 in Table S6, an aliquot was taken at 6 h (38% conversion by ^1H NMR). Chiral GC analysis of this aliquot revealed the residual monomer had an 86 %ee (Figure S42*) indicating that the diastereomeric transition states of $(^{iPr}BDI)ZnOR$ do not exhibit any stereoselectivity.*

Table S7: Polymer characterization data for syndioenriched *trans*-PHMB sample.

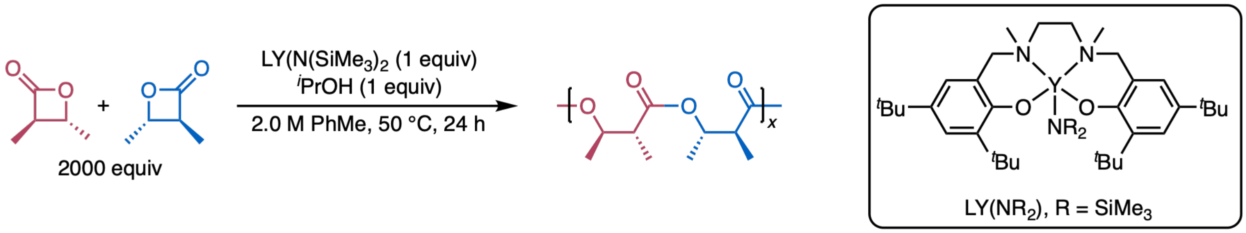

Entry	$M_{n,SEC}$ (kDa)[a]	$Đ$[a]	T_m (°C)[b]	*rr*%[c]
1	115.1	1.32	149	66

[a] Determined by SEC in THF at 30 °C, calibrated relative to monodisperse polystyrene standards. [b] Peak T_m values determined by DSC. [c] Determined by $^{13}C\{^1H\}$ NMR spectroscopic analysis using the carbon α to the carbonyl.

3.6 Polymer characterization of high molecular weight samples

Table S8: Polymer synthesis information and molecular weight data for high molecular weight isoenriched *trans*-PHMB samples used for tensile testing.

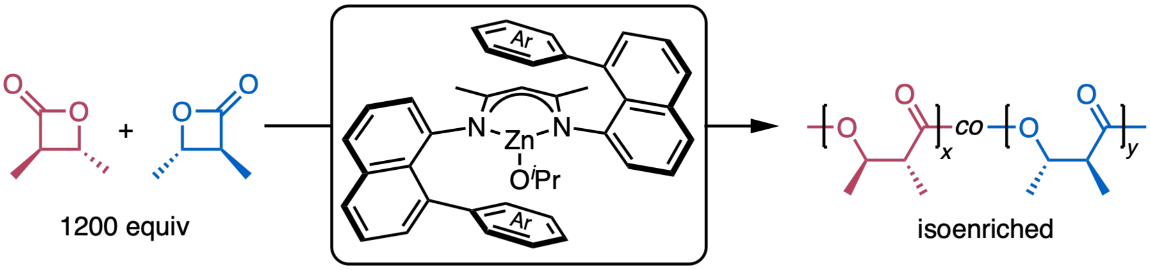

entry	catalyst	T_{rxn} (°C)	t_{rxn} (d)	Conv. (%)^a	$M_{n,theo}$ (kDa)	$M_{n,SEC}$ (kDa)^b	$Đ^b$	*mm%*^{c}
1	($^{4\text{-Me}}$BDI*)ZnO^{i}Pr	40	0.75	>99	120.1	110.3	1.15	68
2	($^{4\text{-Me}}$BDI*)ZnO^{i}Pr	25	0.75	99	118.9	107.3	1.04	73
3	($^{4\text{-Me}}$BDI*)ZnO^{i}Pr	0	1.75	>99	120.1	106.9	1.09	75
4	($^{2\text{-naphthyl}}$BDI*)ZnO^{i}Pr	0	7.00	96	96.1	66.4	1.99	78

^{a}Determined by ^{1}H NMR spectroscopic analysis comparing the relative integration of polymer and residual monomer. ^{b}Determined by SEC in THF at 30 °C, calibrated relative to monodisperse polystyrene standards. ^{c}Determined by ^{13}C{^{1}H} NMR spectroscopic analysis using the carbon α to the carbonyl.

Table S9: DSC analysis of thermal properties of *trans*-PHMB samples used for tensile testing.

entry	*mm%*	T_m (°C)^a	ΔH_m (J/g)^b	χ_c (%)^c
1	68	145	21	24
2	73	154/138	13	15
3	75	161	12	14
4	78	172/157	6	7

^{a}Determined by DSC. ^{d}Determined from integration of endothermic melting transition in the DSC thermogram. ^{c}Calculated using $\chi_c = (\Delta H_m/\Delta H_m°) \times 100$. $\Delta H_m°$ was previously determined to be 88 J/g.[14]

3.7 Other polymerizations catalyzed by *rac*-($^{4\text{-Me}}$BDI*)ZnO^{i}Pr

Table S10: Polymer characterization data for high molecular weight isoenriched *trans*-PHMB samples made with CTA.

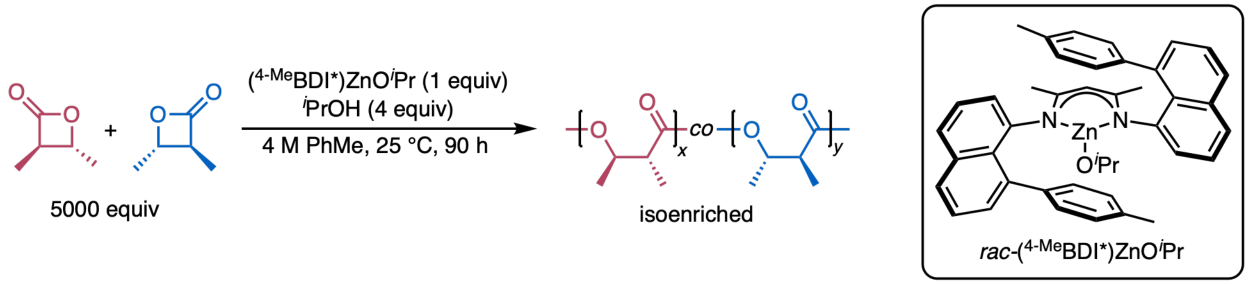

Entry	Conv. (%)[a]	$M_{n,theo}$ (kDa)	$M_{n,SEC}$ (kDa)[b]	$Đ$[b]	*mm*%[c]	T_m (°C)[d]
1	>99	100.1	93.9	1.10	73	153/134

[a]Determined by ^{1}H NMR spectroscopic analysis comparing the relative integration of polymer and residual monomer. [b]Determined by SEC in THF at 30 °C, calibrated relative to monodisperse polystyrene standards. [c]Determined by ^{13}C{^{1}H} NMR spectroscopic analysis using the carbon α to the carbonyl. [d]Determined by DSC.

Table S11: Polymer characterization data for polymerization of *cis*-DMPL by *rac*-($^{4\text{-Me}}$BDI*)ZnO^{i}Pr.

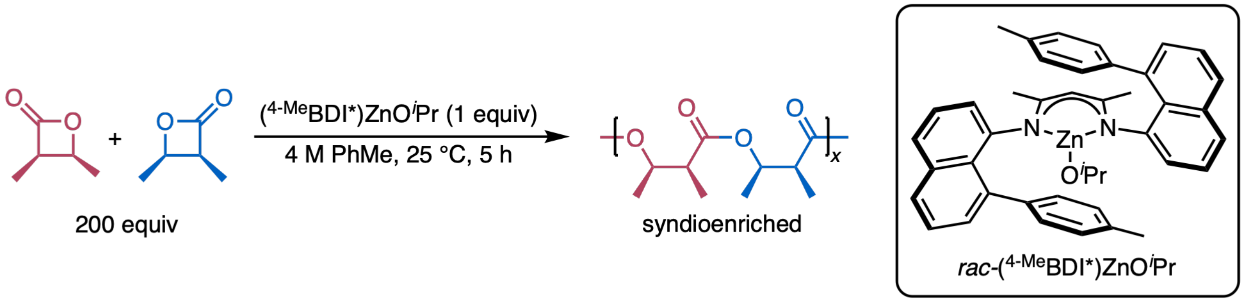

Entry	Conv. (%)[a]	$M_{n,theo}$ (kDa)	$M_{n,SEC}$ (kDa)[b]	$Đ$[b]	*r*%[c]
1	98	19.6	27.2	1.30	56

[a]Determined by ^{1}H NMR spectroscopic analysis comparing the relative integration of polymer and residual monomer. [b]Determined by SEC in THF at 30 °C, calibrated relative to monodisperse polystyrene standards. [c]Determined by ^{13}C{^{1}H} NMR spectroscopic analysis.[3]

3.8 SEC chromatograms

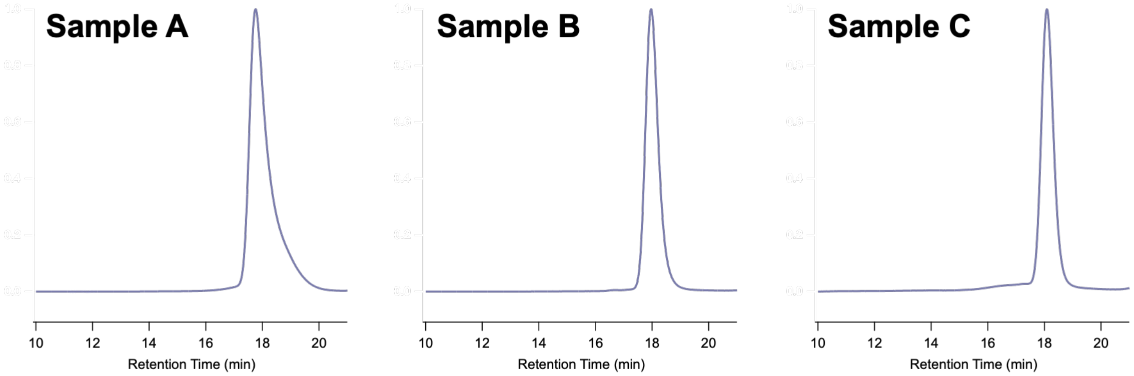

Figure S7: SEC chromatograms of enantioenriched *trans*-PHMB used to determine stereoerrors in Table S1 and Table S2.

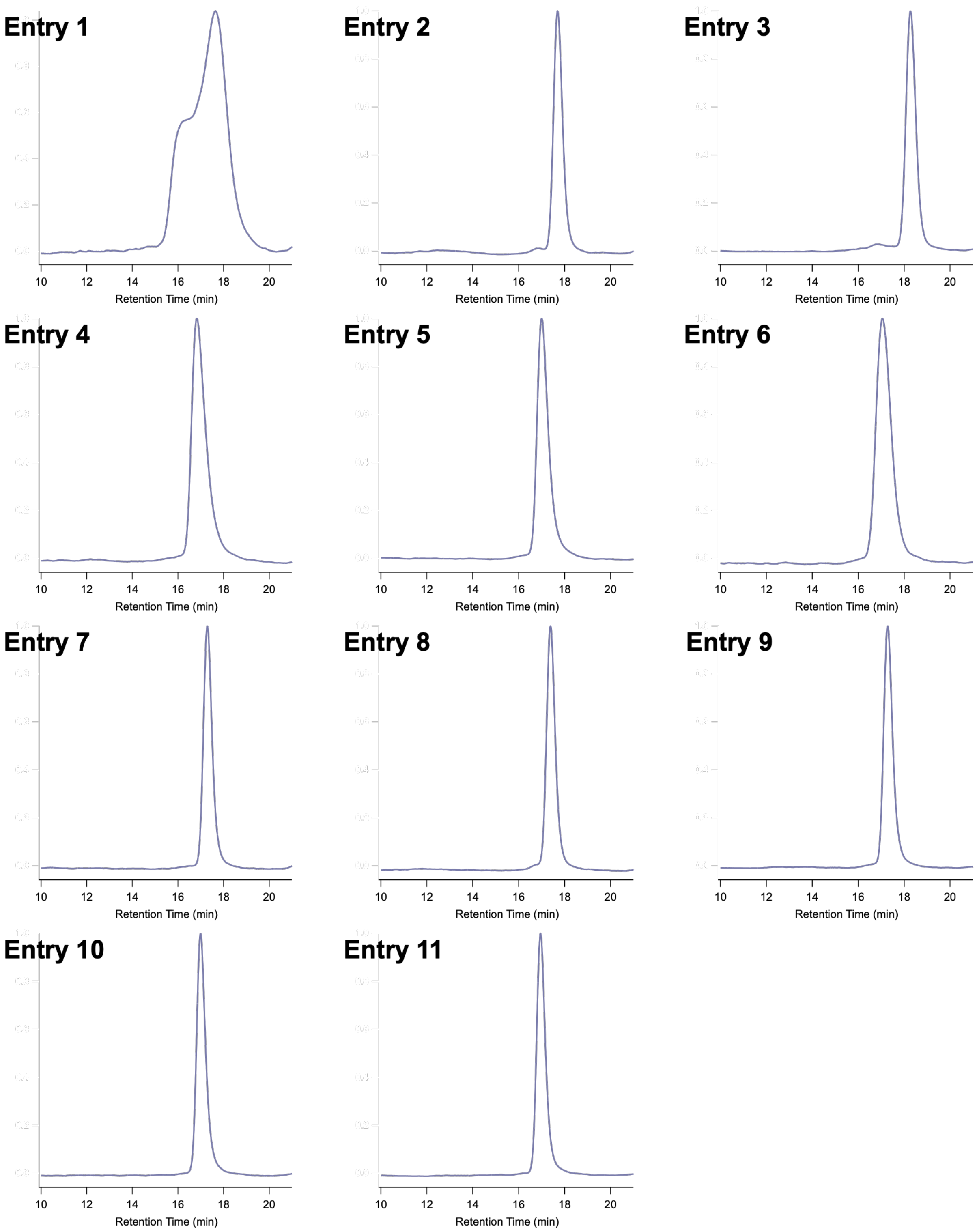

Figure S8: SEC chromatograms of the *trans*-PHMB samples from condition optimization (Table S4).

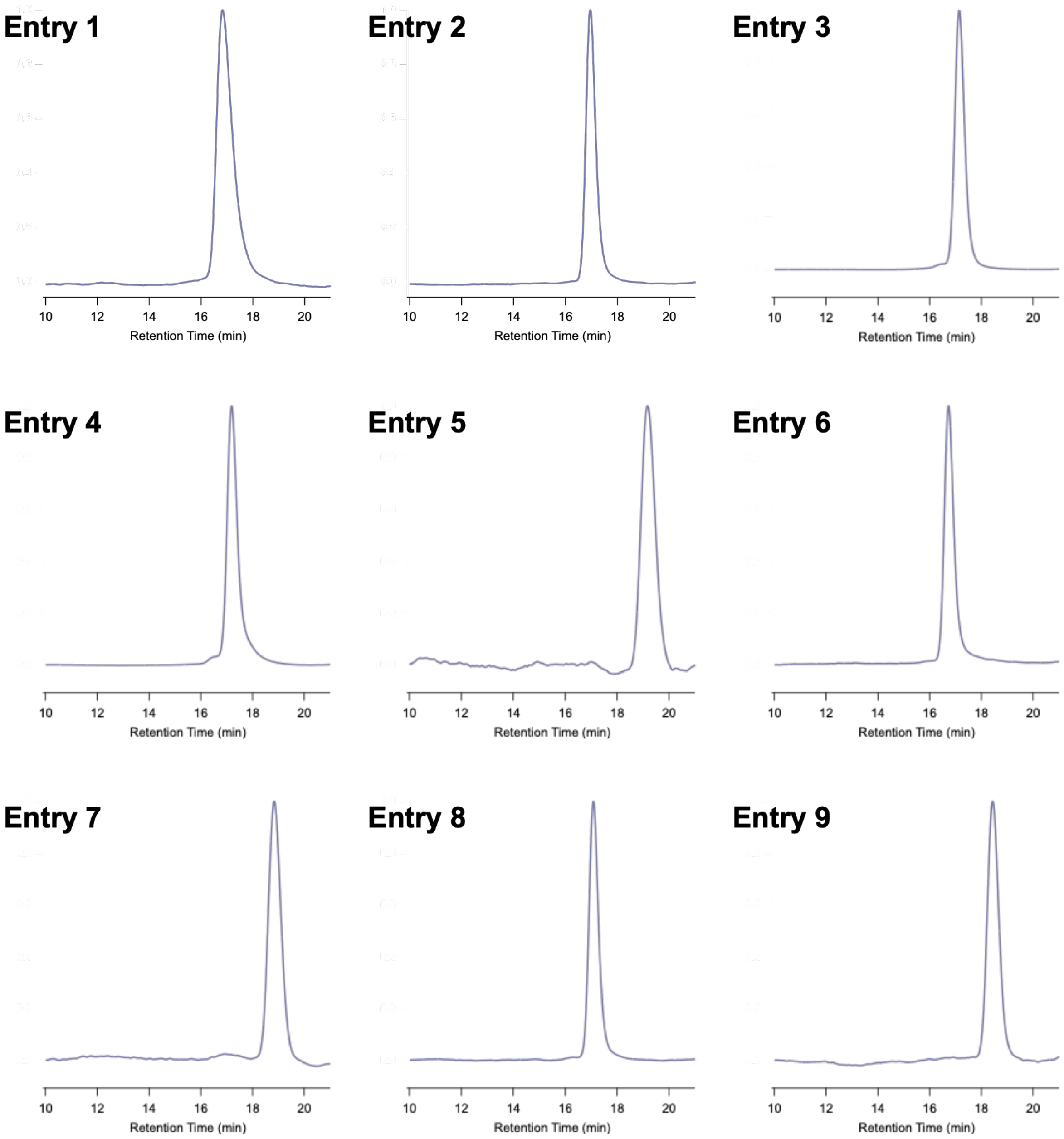

Figure S9: SEC chromatograms of the *trans*-PHMB made in the catalyst screen in Table 1.

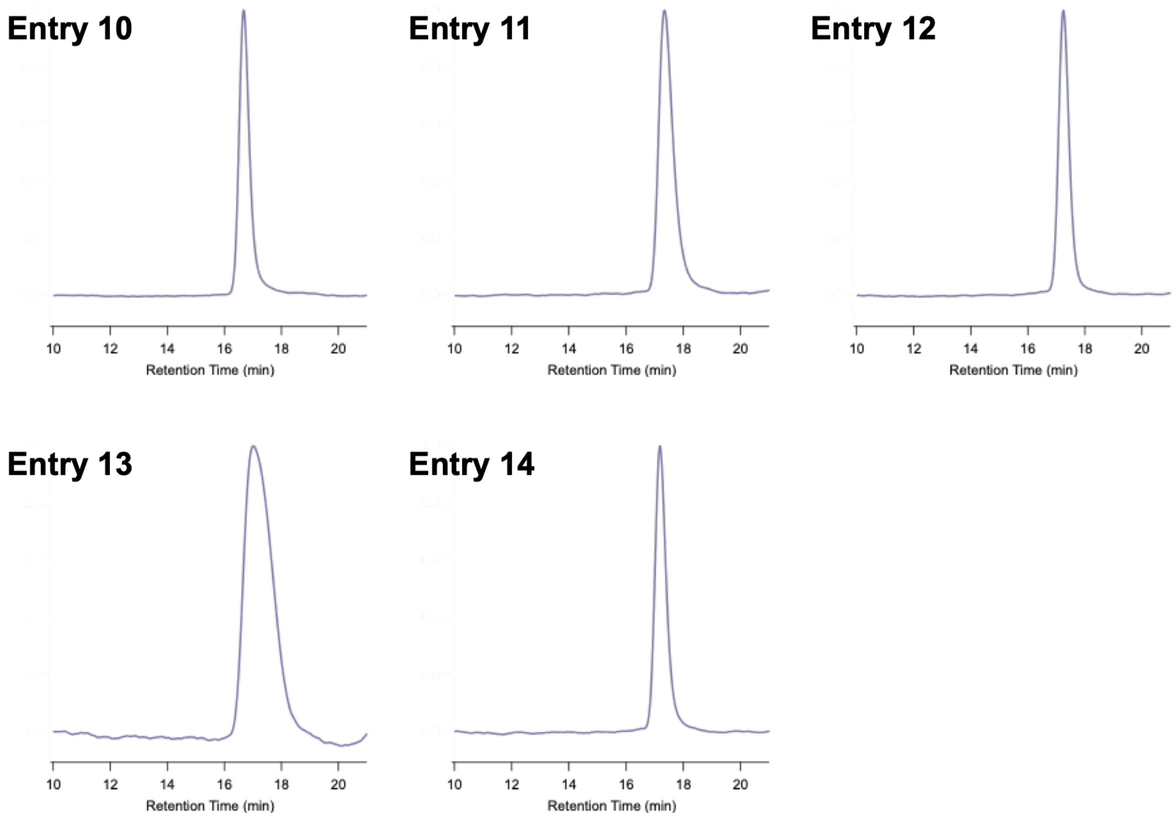

Figure S9 continued: SEC chromatograms of the *trans*-PHMB made in the catalyst screen in Table 1.

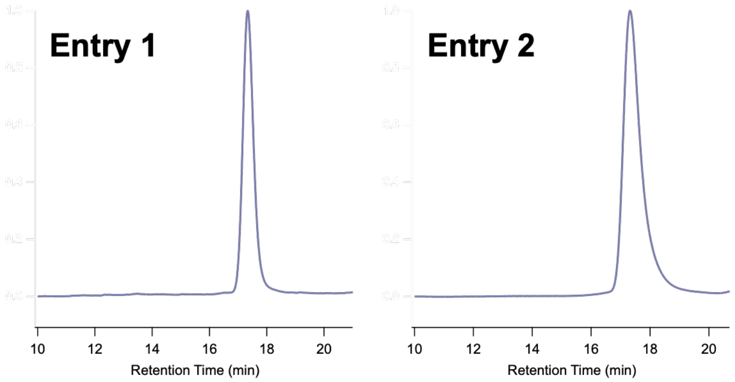

Figure S10: SEC chromatograms of *trans*-PHMB samples made by $[(^{iPr}BDI)ZnO^{i}Pr]_2$ in Table S6.

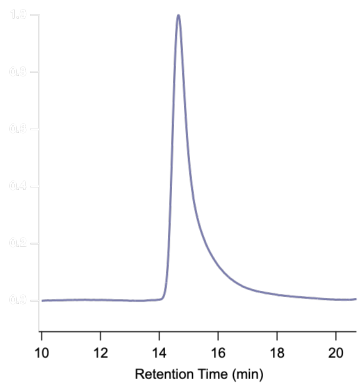

Figure S11: SEC chromatogram of syndioenriched *trans*-PHMB in Table S7.

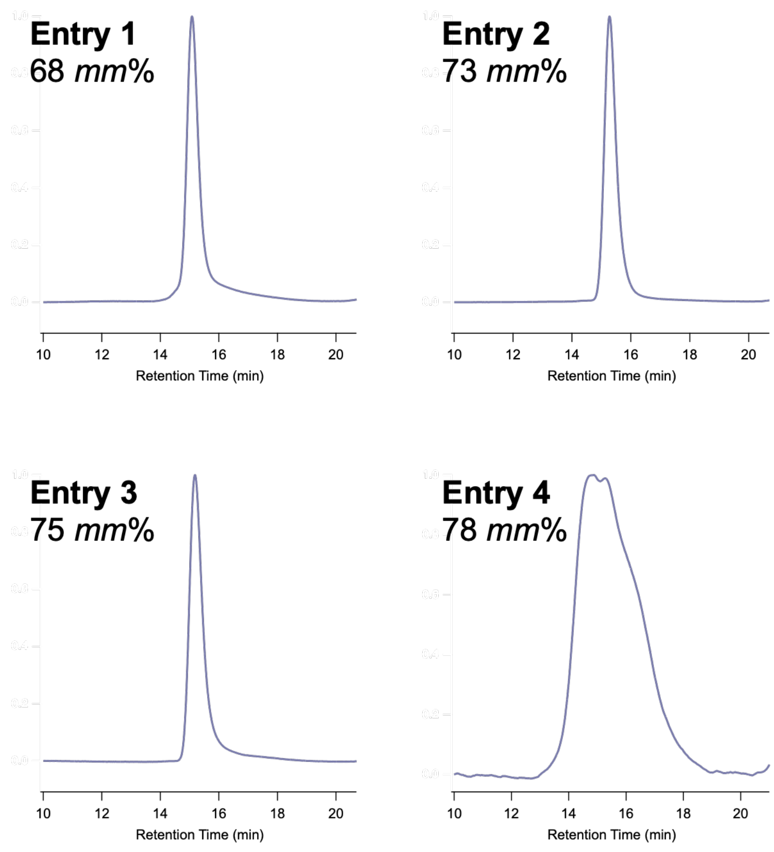

Figure S12: SEC chromatogram of high molecular weight isoenriched *trans*-PHMB samples used for tensile testing (Table S8).

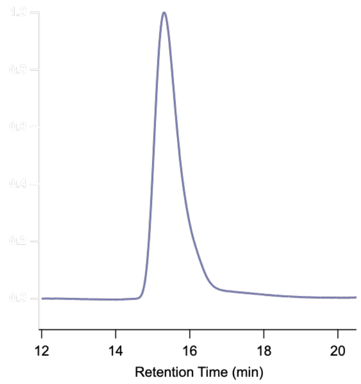

Figure S13: SEC chromatogram of high molecular weight isoenriched *trans*-PHMB made using CTA (Table S10).

3.9 DSC thermograms

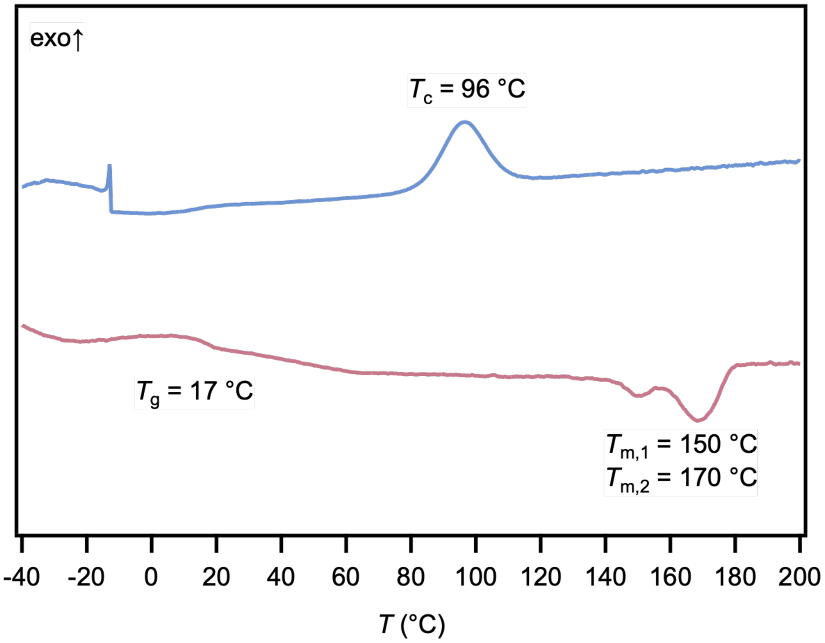

Figure S14: DSC thermogram showing 1st cool (blue) and 2nd heat (red) of isoenriched *trans*-PHMB (77 *mm*%; Table 1, entry 1).

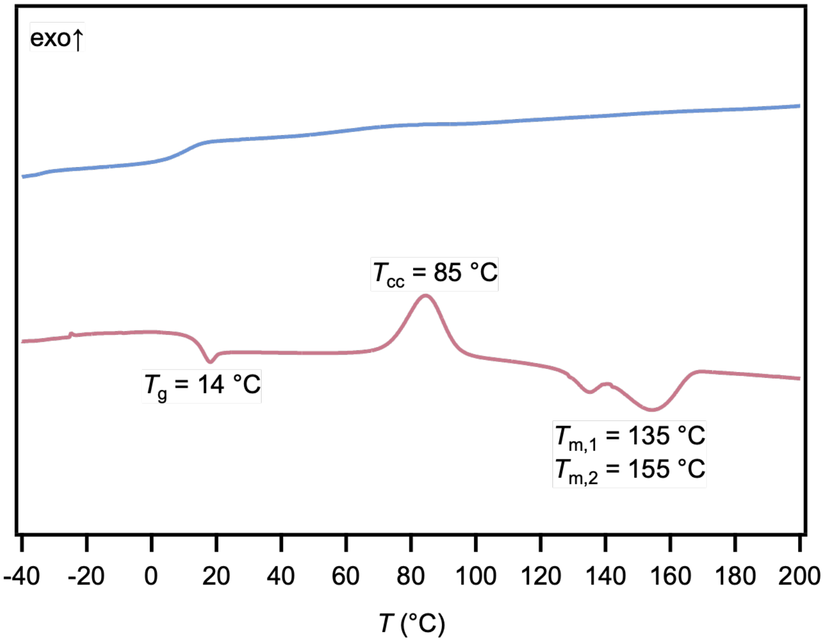

Figure S15: DSC thermogram showing 1st cool (blue) and 2nd heat (red) of isoenriched *trans*-PHMB (73 *mm*%; Table 1, entry 2).

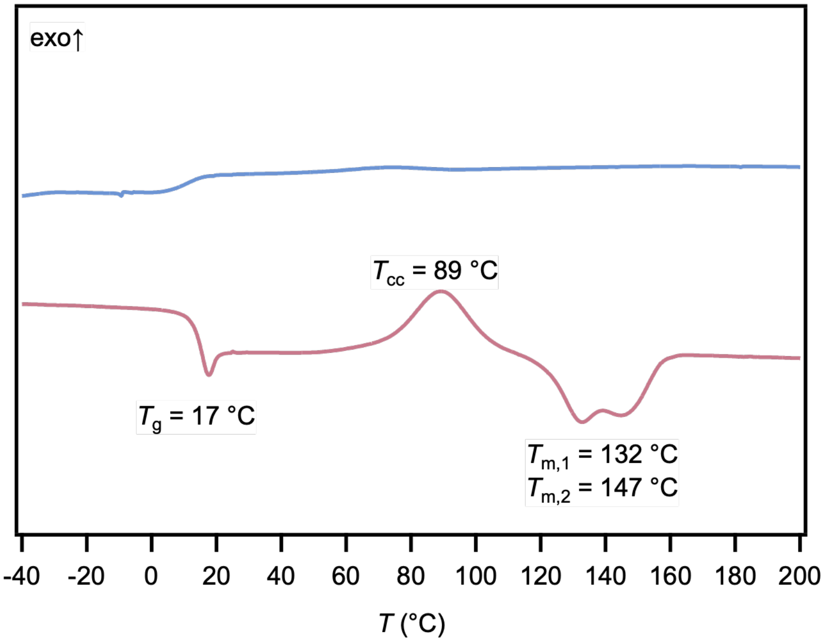

Figure S16: DSC thermogram showing 1st cool (blue) and 2nd heat (red) of isoenriched *trans*-PHMB (69 *mm*%; Table 1, entry 3).

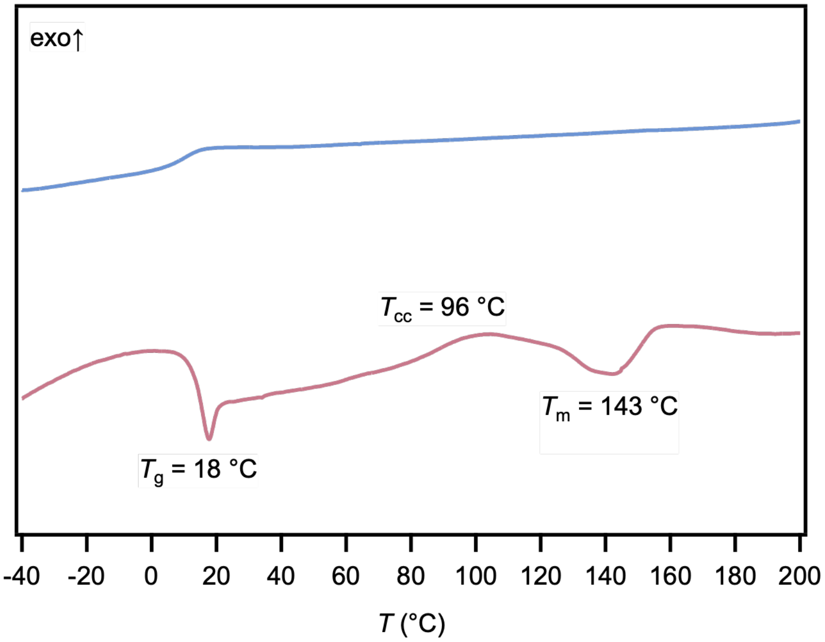

Figure S17: DSC thermogram showing 1st cool (blue) and 2nd heat (red) of isoenriched *trans*-PHMB (66 *mm*%; Table 1, entry 4).

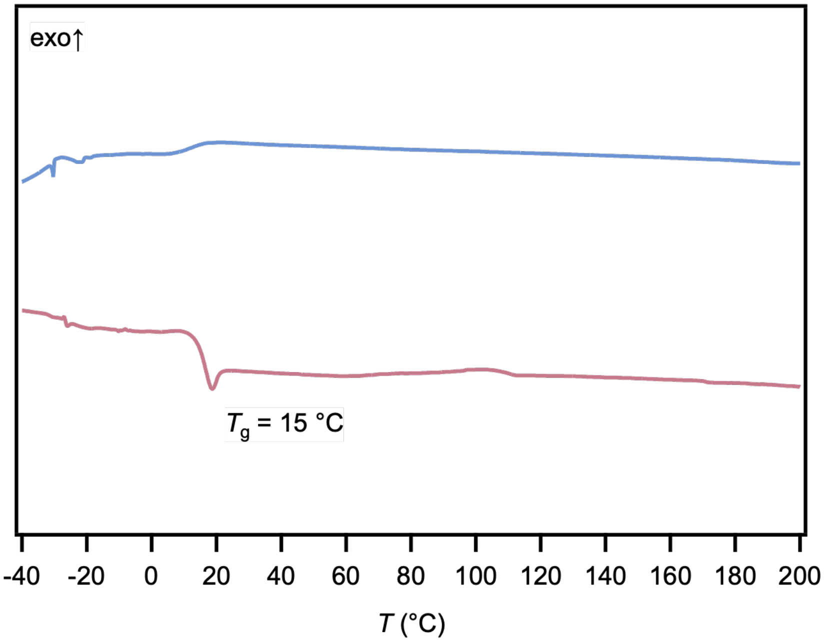

Figure S18: DSC thermogram showing 1st cool (blue) and 2nd heat (red) *trans*-PHMB (69 *mm*%; Table 1, entry 6).

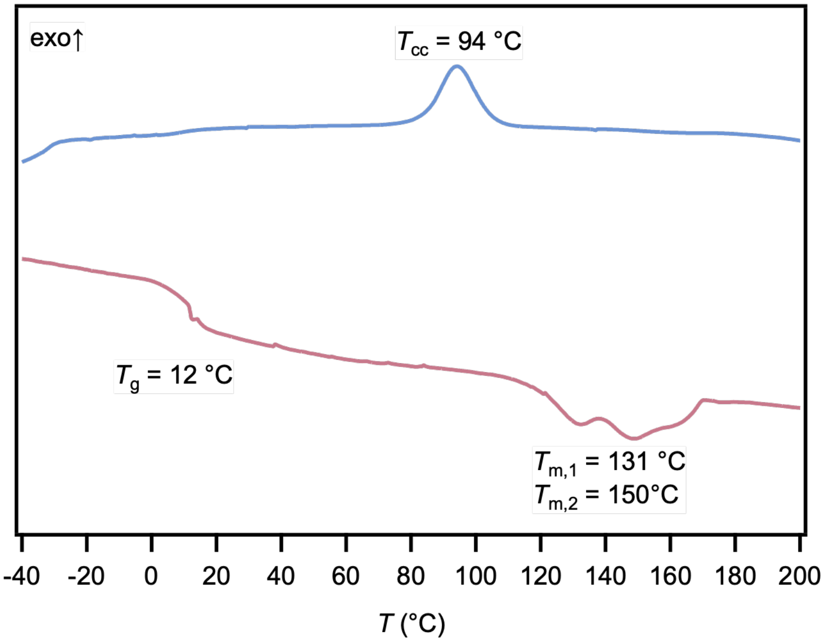

Figure S19: DSC thermogram showing 1st cool (blue) and 2nd heat (red) of *trans*-PHMB (61 *mm*%; Table 1, entry 7).

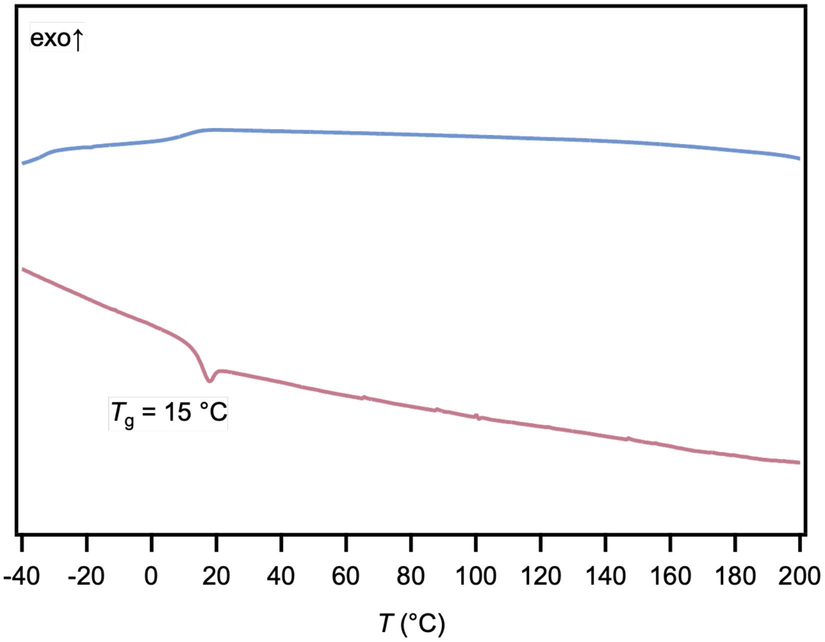

Figure S20: DSC thermogram showing 1st cool (blue) and 2nd heat (red) of isoenriched *trans*-PHMB (55 *mm*%; Table 1, entry 8).

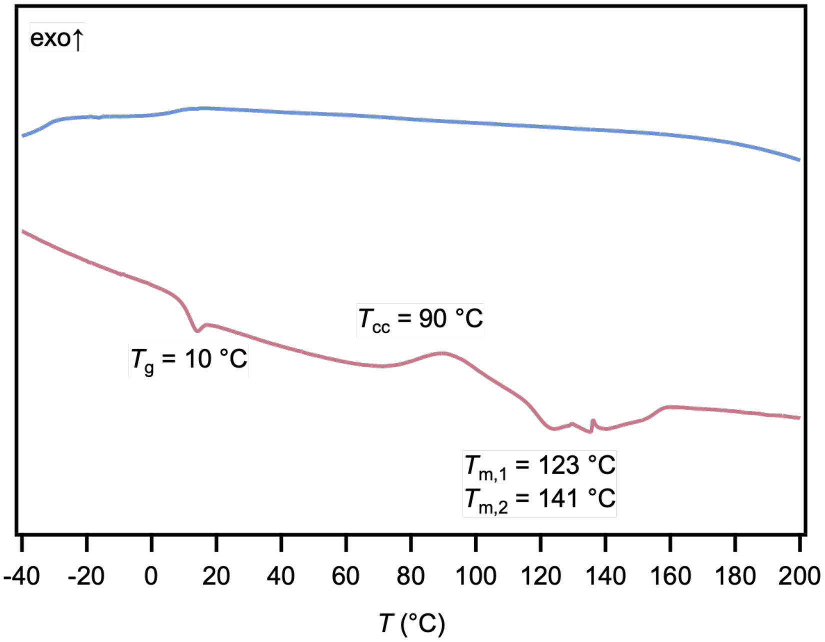

Figure S21: DSC thermogram showing 1st cool (blue) and 2nd heat (red) of isoenriched *trans*-PHMB (Table 1, entry 9).

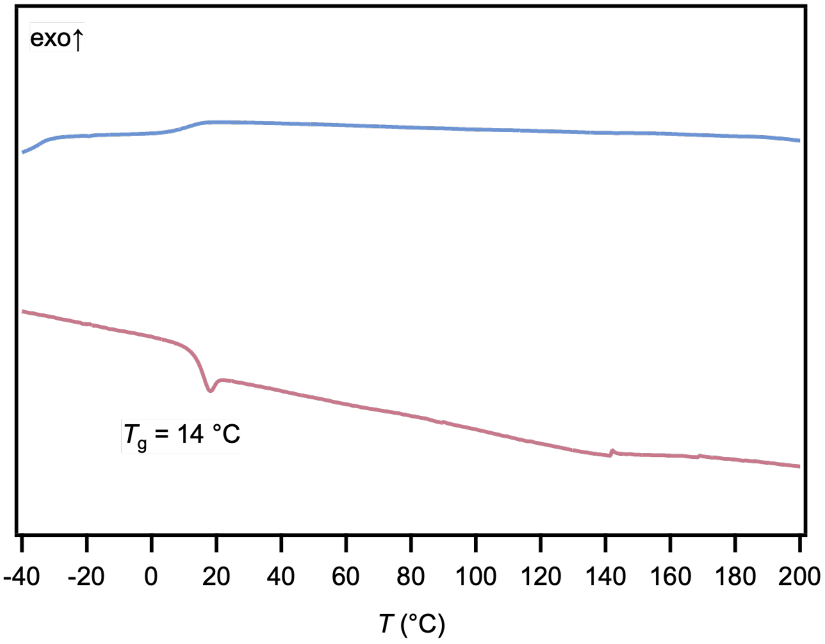

Figure S22: DSC thermogram showing 1st cool (blue) and 2nd heat (red) of isoenriched *trans*-PHMB (54 *mm*%; Table 1, entry 10).

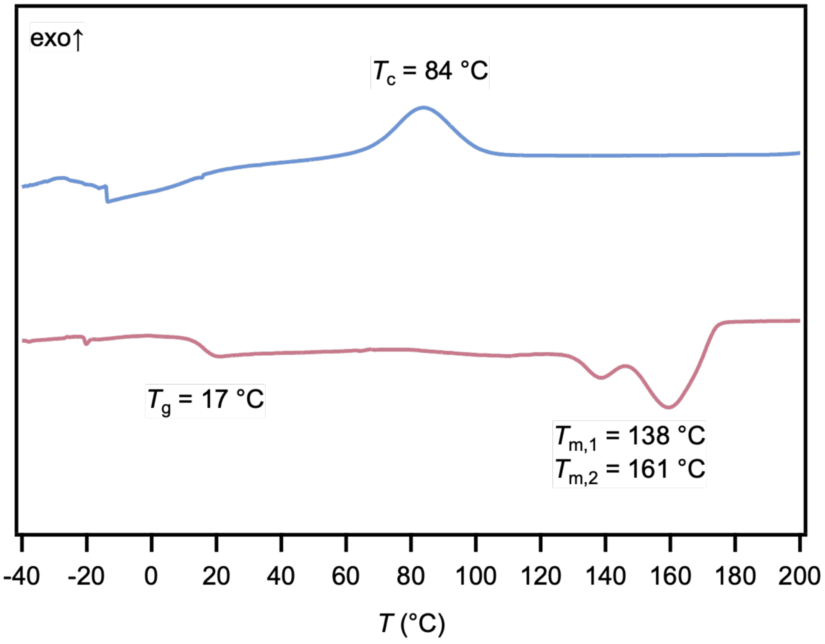

Figure S23: DSC thermogram showing 1st cool (blue) and 2nd heat (red) of isoenriched *trans*-PHMB (75 *mm*%; Table 1, entry 11).

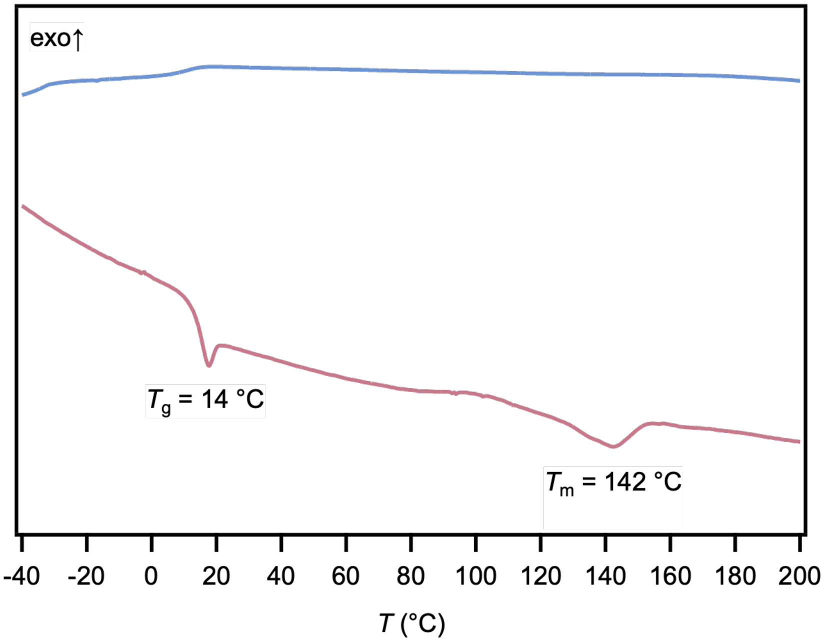

Figure S24: DSC thermogram showing 1st cool (blue) and 2nd heat (red) of isoenriched *trans*-PHMB (65 *mm*%; Table 1, entry 12).

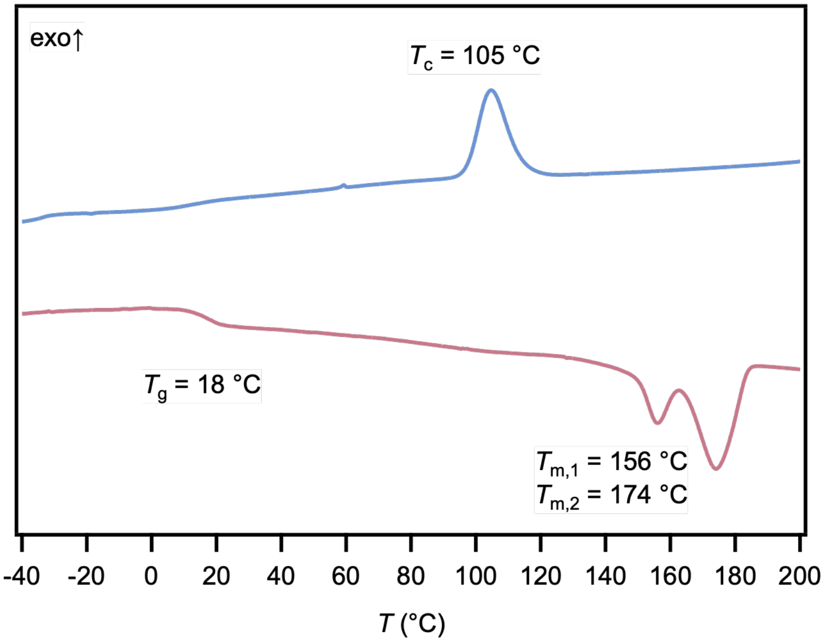

Figure S25: DSC thermogram showing 1st cool (blue) and 2nd heat (red) of isoenriched *trans*-PHMB (79 *mm*%; Table 1, entry 13).

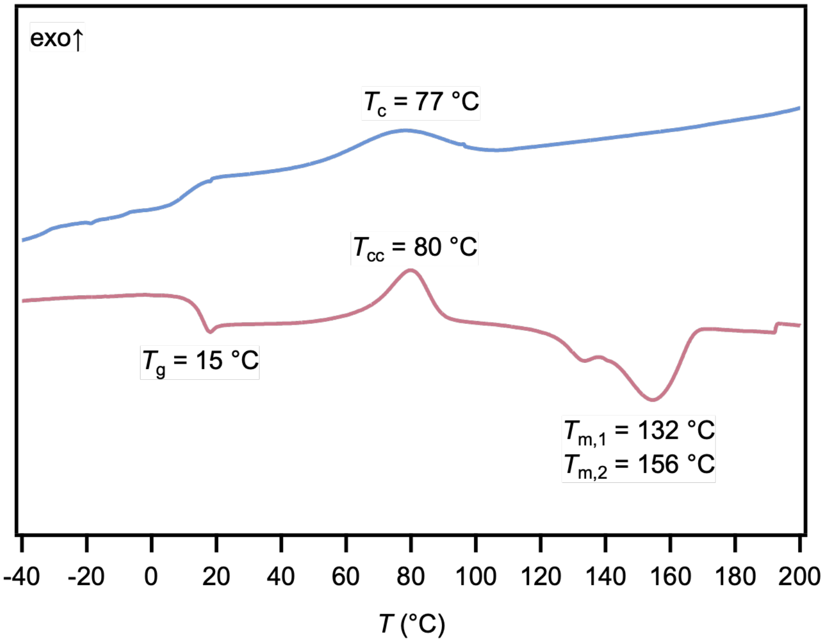

Figure S26: DSC thermogram showing 1st cool (blue) and 2nd heat (red) of isoenriched *trans*-PHMB (73 *mm*%; Table 1, entry 14).

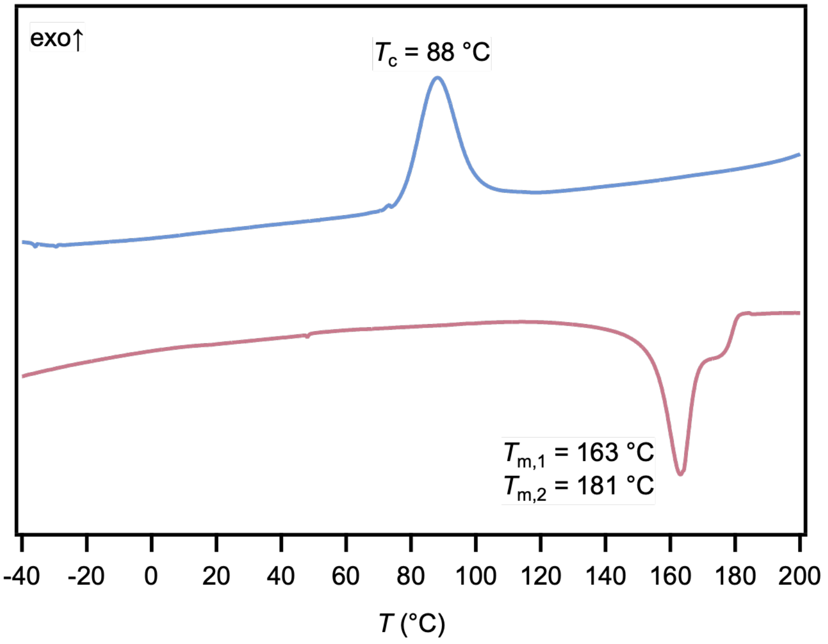

Figure S27: DSC thermogram showing 1st cool (blue) and 2nd heat (red) for Sample A made from enantioenriched *trans*-DMPL in Table S1.

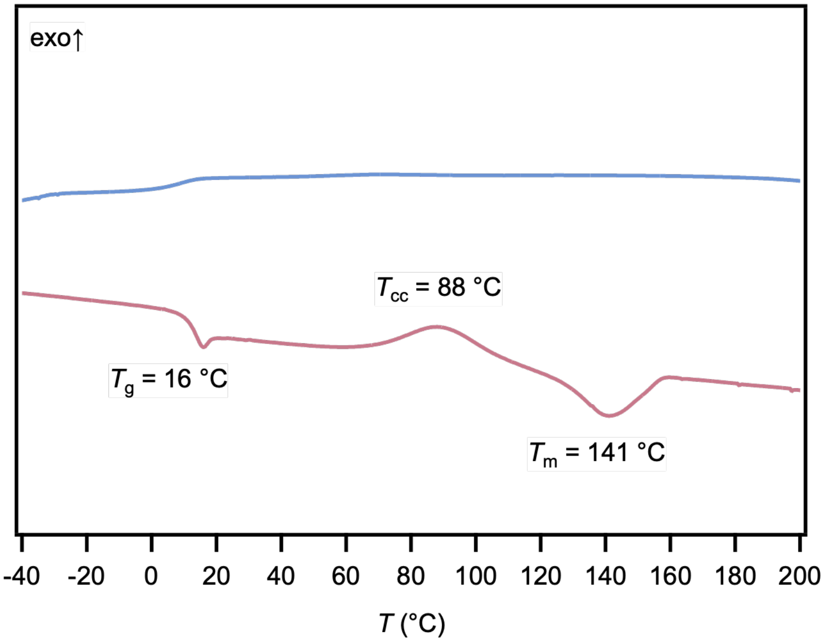

Figure S28: DSC thermogram showing 1st cool (blue) and 2nd heat (red) for Sample B made from enantioenriched *trans*-DMPL in Table S1.

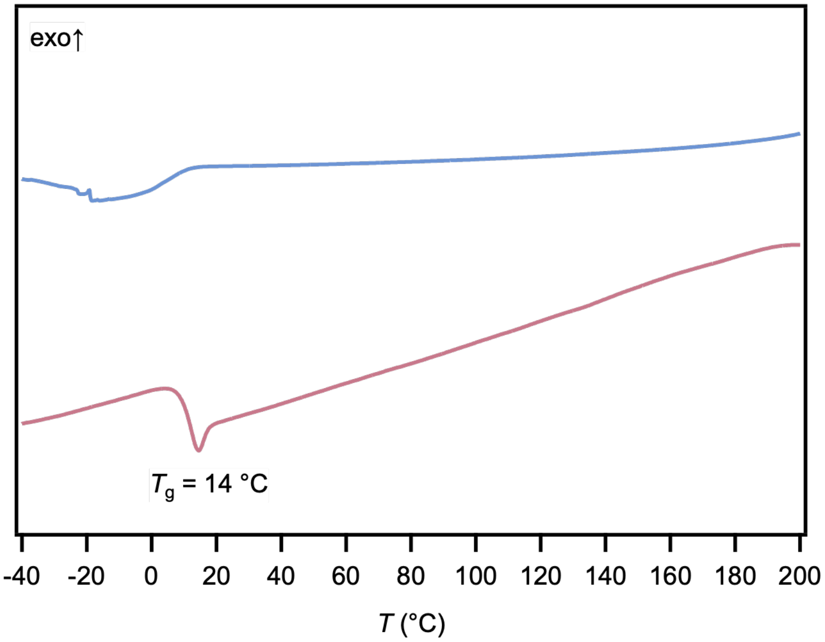

Figure S29: DSC thermogram showing 1st cool (blue) and 2nd heat (red) for Sample C made from enantioenriched *trans*-DMPL in Table S1.

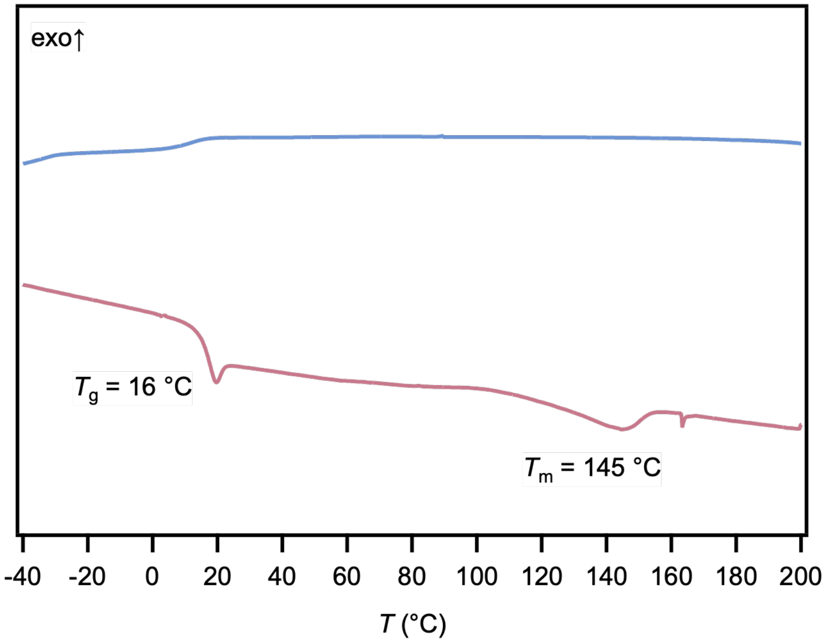

Figure S30: DSC thermogram showing 1st cool (blue) and 2nd heat (red) for high molecular weight *trans*-PHMB sample used for tensile testing with 68 *mm*% (Table S9, entry 1).

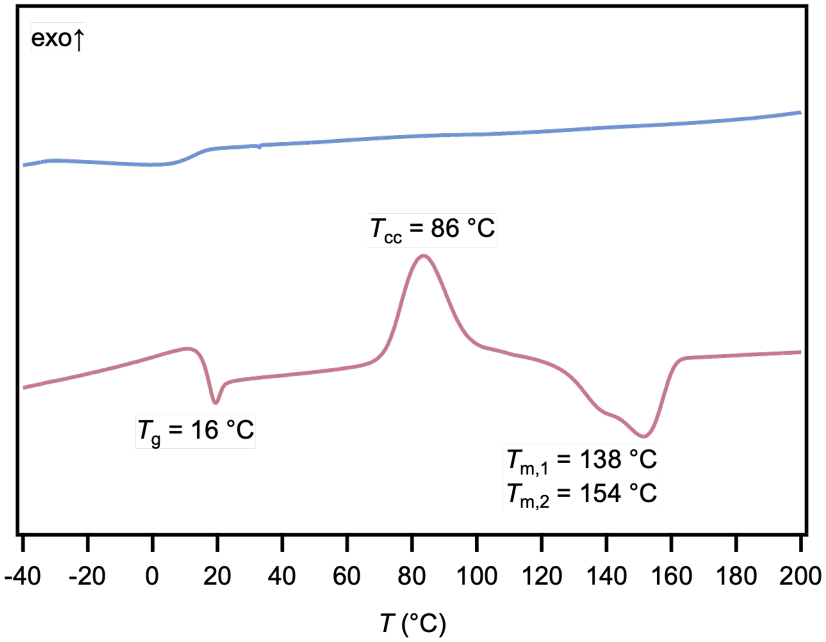

Figure S31: DSC thermogram showing 1st cool (blue) and 2nd heat (red) for high molecular weight *trans*-PHMB sample used for tensile testing with 73 *mm*% (Table S9, entry 2).

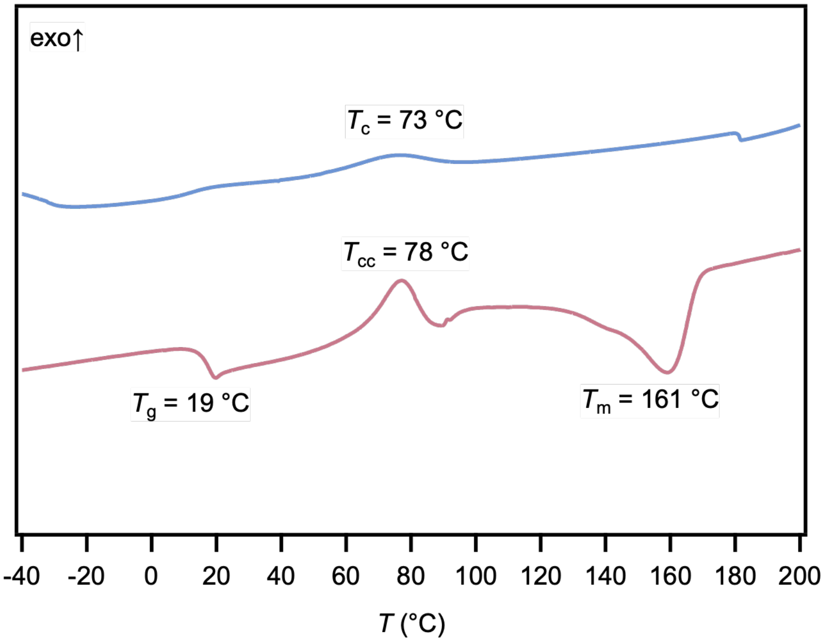

Figure S32: DSC thermogram showing 1st cool (blue) and 2nd heat (red) for high molecular weight *trans*-PHMB sample used for tensile testing with 75 *mm*% (Table S9, entry 3).

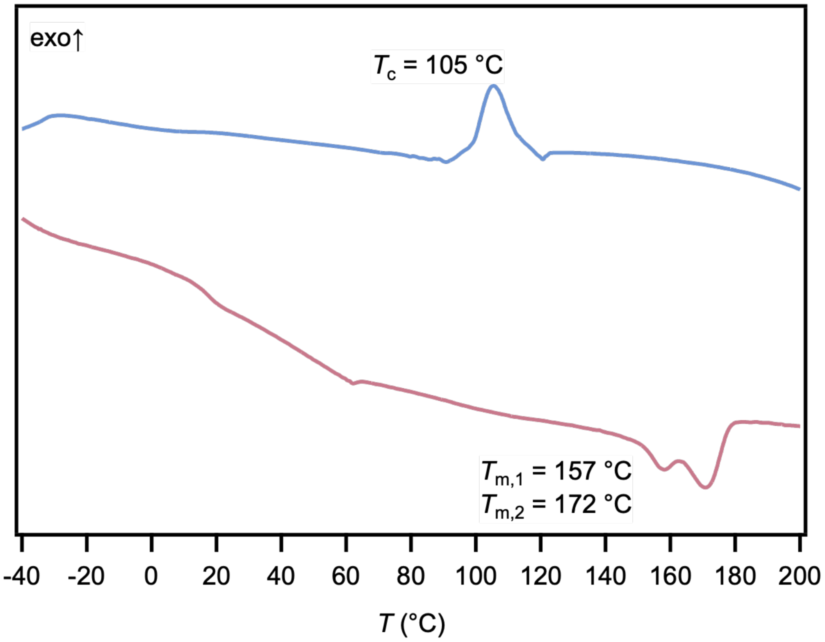

Figure S33: DSC thermogram showing 1st cool (blue) and 2nd heat (red) for high molecular weight *trans*-PHMB sample used for tensile testing with 78 *mm*% (Table S9, entry 4).

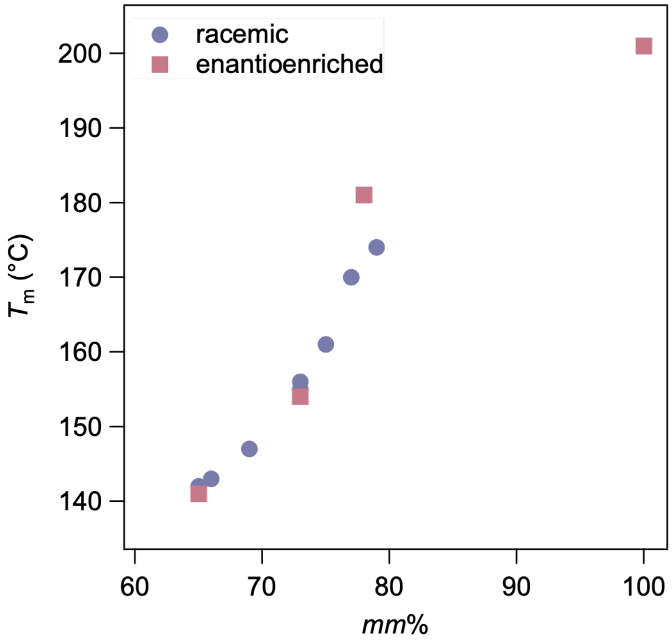

Figure S34: Relationship between T_m and *mm*% for racemic and enantioenriched *trans*-PHMB. T_m of 100 mm% sample is obtained from literature.[14,15]

3.10 Thermal stability

Procedure: The polymer samples (initial M_n = 105.4 kDa, *Đ* = 1.07) were placed in 5 individual DSC aluminum pans (~1.5 mg each), put in the DSC and heated to 170 °C at a rate of 10 °C/min and isothermally held at 170 °C for different lengths of time (1 min to 2 h) under N_2. After, the samples were removed from the pan, dissolved in THF, filtered through a 0.45 µm PTFE syringe filter and their molecular weights measured using SEC analysis.[16]

Table S12: M_n and *Đ* change of isoenriched *trans*-PHMB (*mm%* = 70 %) over time at 170 °C.

Time (min)	$M_{n,SEC}$ (kDa)	*Đ*
0	105.4	1.07
2	111.2	1.04
10	110.2	1.04
30	99.1	1.09
60	84.7	1.21
120	62.1	1.42

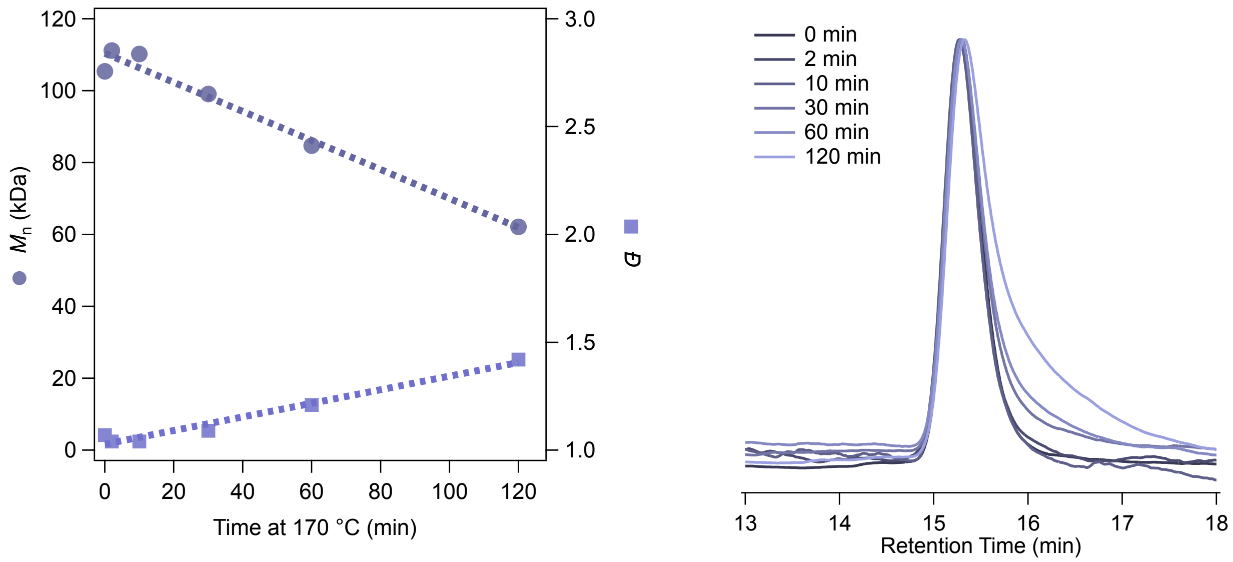

Figure S35: Left: M_n and *Đ* change for isoenriched *trans*-PHMB (*mm%* = 70 %) over time at 170 °C. **Right:** corresponding SEC chromatograms.

3.11 Preparation of tensile samples

The samples were placed in a rectangular mold between two sheets of Teflon between two stainless steel plates before being placed in a preheated Carver press. The samples were pressed for 10 min, quickly released from pressure, pressurized again for 1 min. The 1 min cycle was repeated two additional times. The heater was turned off, and the samples were slowly cooled below their T_c with water cooling under pressure. The pressure was released and the film was let to cool to 25 °C before being cut into ASTM 37-4 standard dog bones using a die cutter.

***Trans*-PHMB *mm*%**	**Melt-press temperature (°C)**	**Melt-press pressure**	**Melt-press time (min)**
68	155	5 metric tons	10 + 1 + 1 + 1
73	165	5 metric tons	10 + 1 + 1 + 1
75	170	5 metric tons	10 + 1 + 1 + 1
78	185	5 metric tons	10 + 1 + 1 + 1

3.12 Tensile properties

Table S13: Consolidated tensile data for isoenriched *trans*-PHMB samples

Sample	Yield Stress, σ_Y (MPa)	Stress at break, σ_B (MPa)	Strain at break, ε_B (%)	Young's modulus, *E* (MPa)
68 *mm*%	-	12.1 ± 0.9	1030 ± 60	58 ± 4
73 *mm*%	8.1 ± 0.4	9.9 ± 1.2	680 ± 130	120 ± 10
75 *mm*%	8.9 ± 0.4	13.00 ± 0.03	750 ± 40	120 ± 10
78 *mm*%	10.0 ± 0.5	9.0 ± 1.0	540 ± 70	130 ± 10

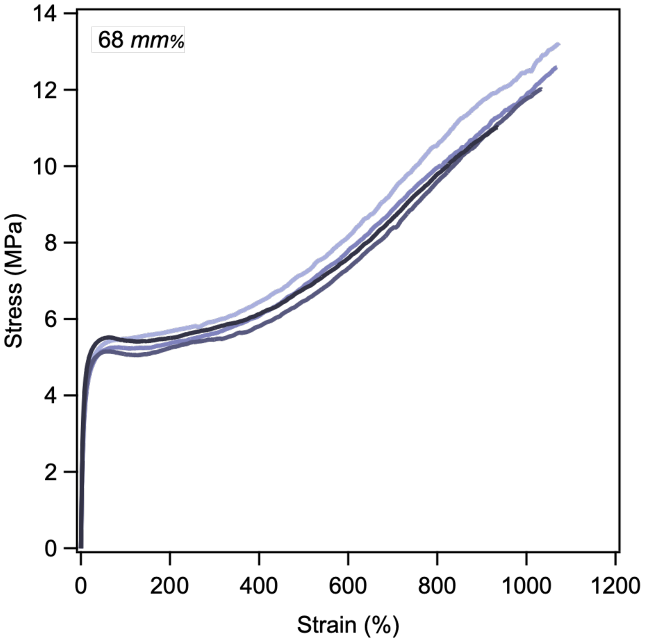

Figure S36: Tensile stress-strain curves for isoenriched *trans*-PHMB (68 *mm%*).

Table S14: Tensile properties for isoenriched *trans*-PHMB (68 *mm%*).

entry	Stress at break, σ_B (MPa)	Strain at break, ε_B (%)	Young's modulus, *E* (MPa)
1	13.1	1070	57
2	12.6	1070	54
3	11.8	1040	57
4	11.0	940	64
Average	12.1	1030	58
Std Deviation	0.9	60	4

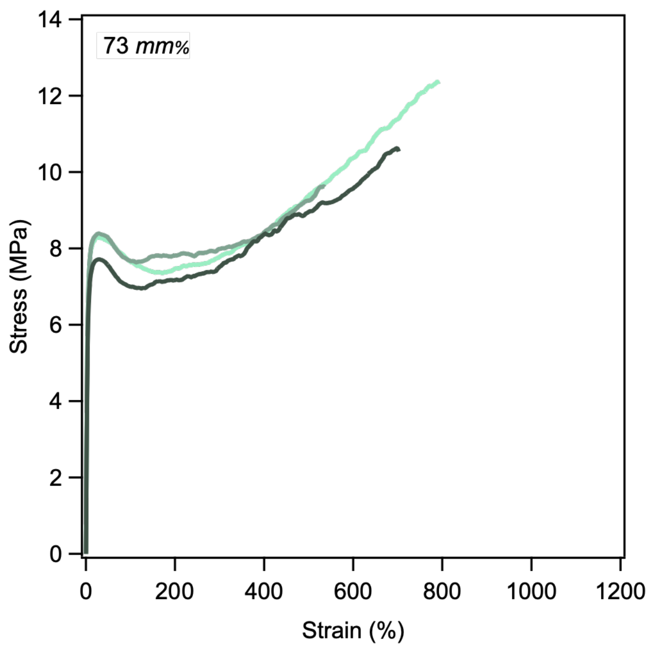

Figure S37: Tensile stress-strain curves for isoenriched *trans*-PHMB (73 *mm%*).

Table S15: Tensile properties for isoenriched *trans*-PHMB with (73 *mm%*).

entry	Yield Stress, σ_Y (MPa)	Stress at break, σ_B (MPa)	Strain at break, ε_B (%)	Young's modulus, E (MPa)
1	8.3	11.3	790	130
2	8.4	8.9	540	120
3	7.7	9.5	710	110
Average	8.1	9.9	680	120
Std Deviation	0.4	1.2	130	10

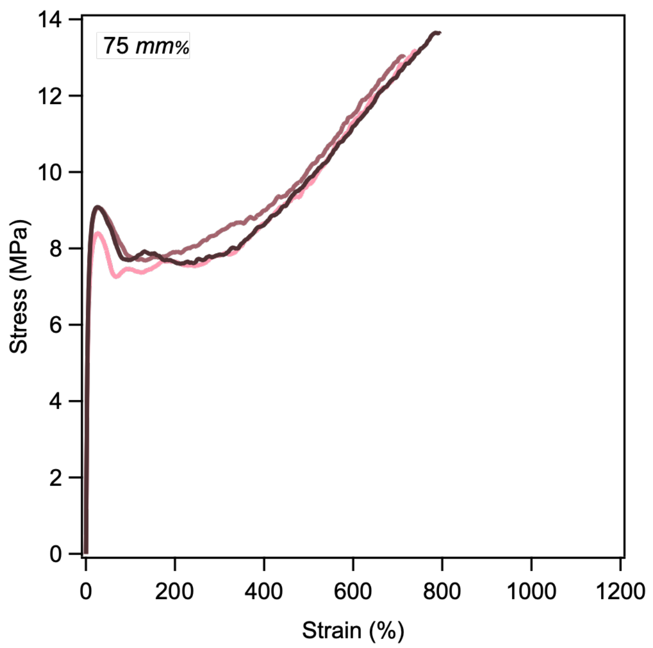

Figure S38: Tensile stress-strain curves for isoenriched *trans*-PHMB (75 *mm%*).

Table S16: Tensile properties for isoenriched *trans*-PHMB (75 *mm%*).

entry	Yield Stress, σ_Y (MPa)	Stress at break, σ_B (MPa)	Strain at break, ε_B (%)	Young's modulus, *E* (MPa)
1	8.4	13.00	740	110
2	9.1	13.00	710	120
3	9.1	13.00	800	120
Average	8.9	13.00	750	120
Std Deviation	0.4	0.03	40	10

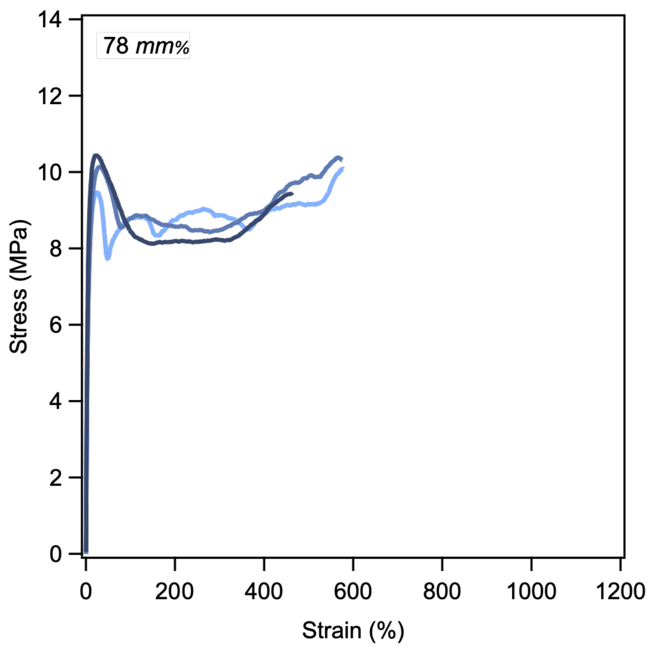

Figure S39: Tensile stress-strain curves for isoenriched *trans*-PHMB (78 *mm%*).

Table S17: Tensile properties for isoenriched *trans*-PHMB (78 *mm%*).

entry	Yield Stress, σ_Y (MPa)	Stress at break, σ_B (MPa)	Strain at break, ε_B (%)	Young's modulus, *E* (MPa)
1	9.5	10.1	580	120
2	10.1	8.9	580	120
3	10.5	8.2	460	140
Average	10.0	9.0	540	130
Std Deviation	0.5	1.0	70	10

3.13 Chiral GC chromatograms

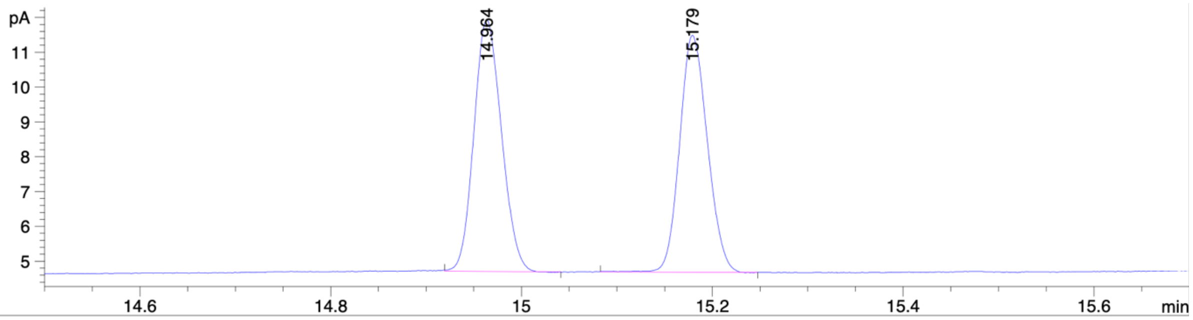

Figure S40: Chiral GC Chromatogram of *rac*-*trans*-DMPL.

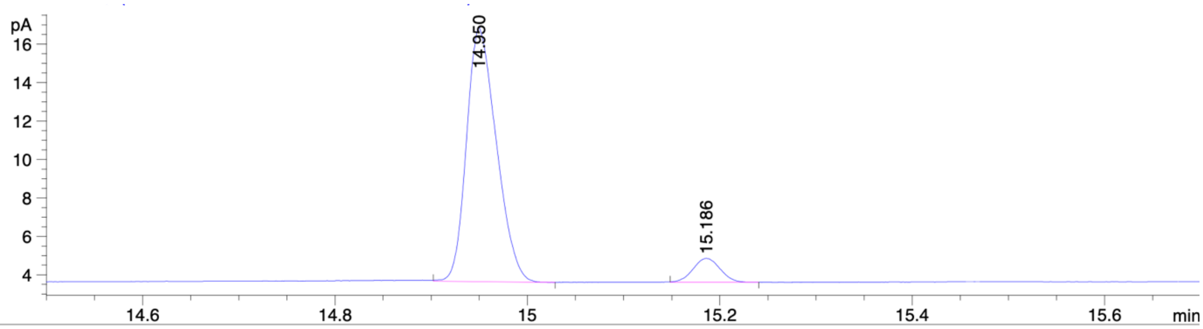

Figure S41: Chiral GC Chromatogram of enantioenriched (86 %*ee*) (*R,R*)-*trans*-DMPL.

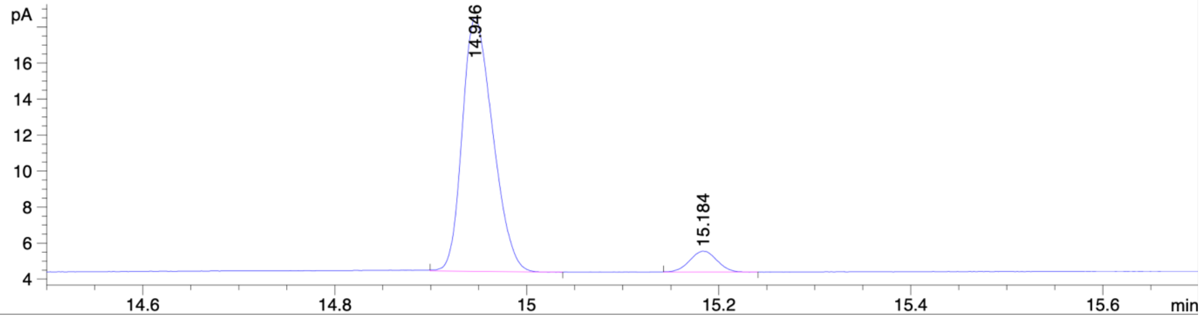

Figure S42: Chiral GC Chromatogram of residual monomer (at 6h, 38% conversion) from a polymerization of 86 %*ee* (*R,R*)-*trans*-DMPL with [(^{iPr}BDI)ZnO^{i}Pr]$_2$ (Table S6).

4 NMR Spectra

4.1 8-Arylnaphthylamines

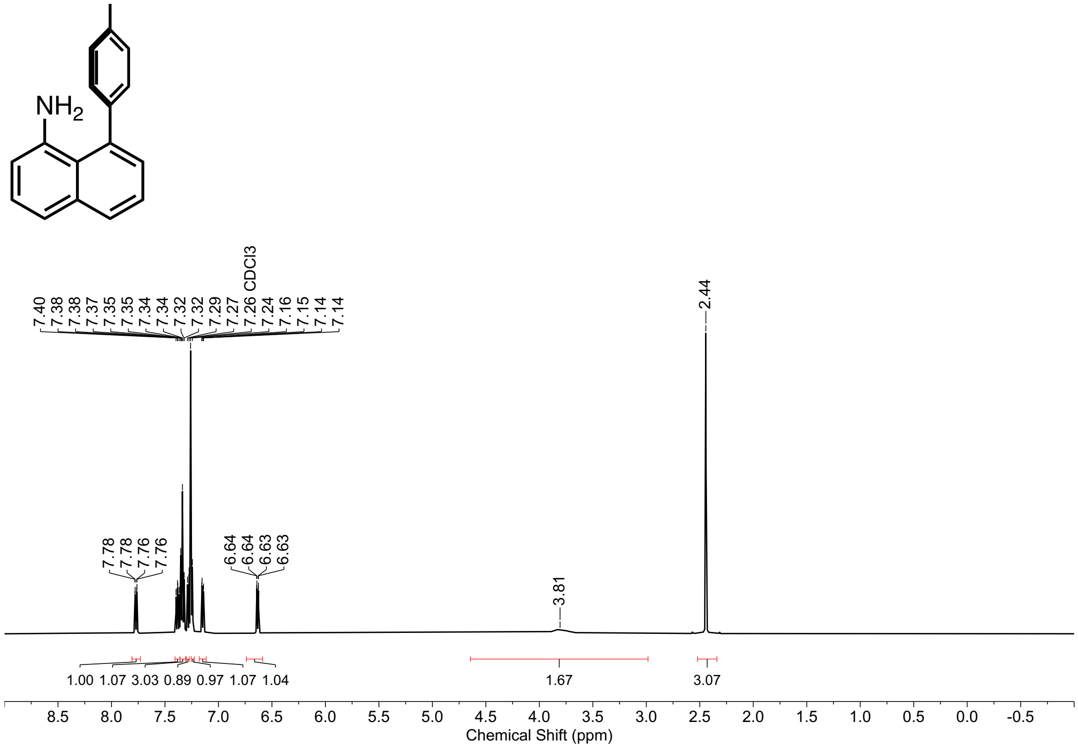

Figure S43: ^{1}H NMR (500 MHz, $CDCl_3$) spectrum of 8-(4-methylphenyl)naphthalen-1-amine.

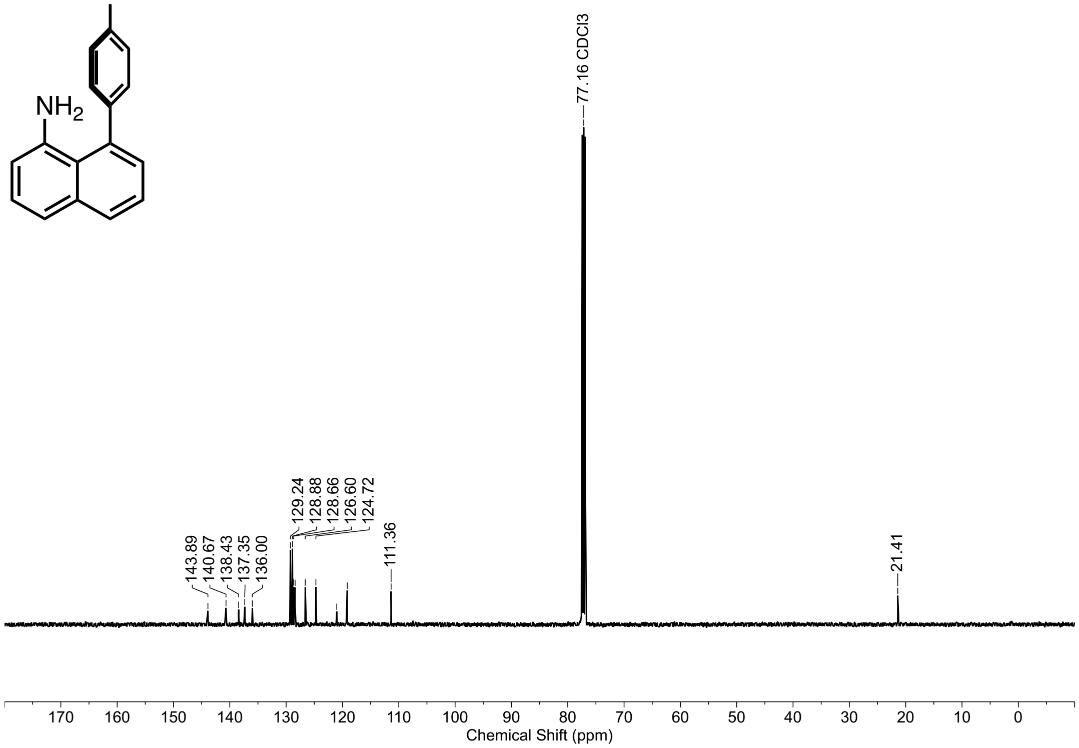

Figure S44: $^{13}C\{^{1}H\}$ NMR (126 MHz, $CDCl_3$) spectrum of 8-(4-methylphenyl)naphthalen-1-amine.

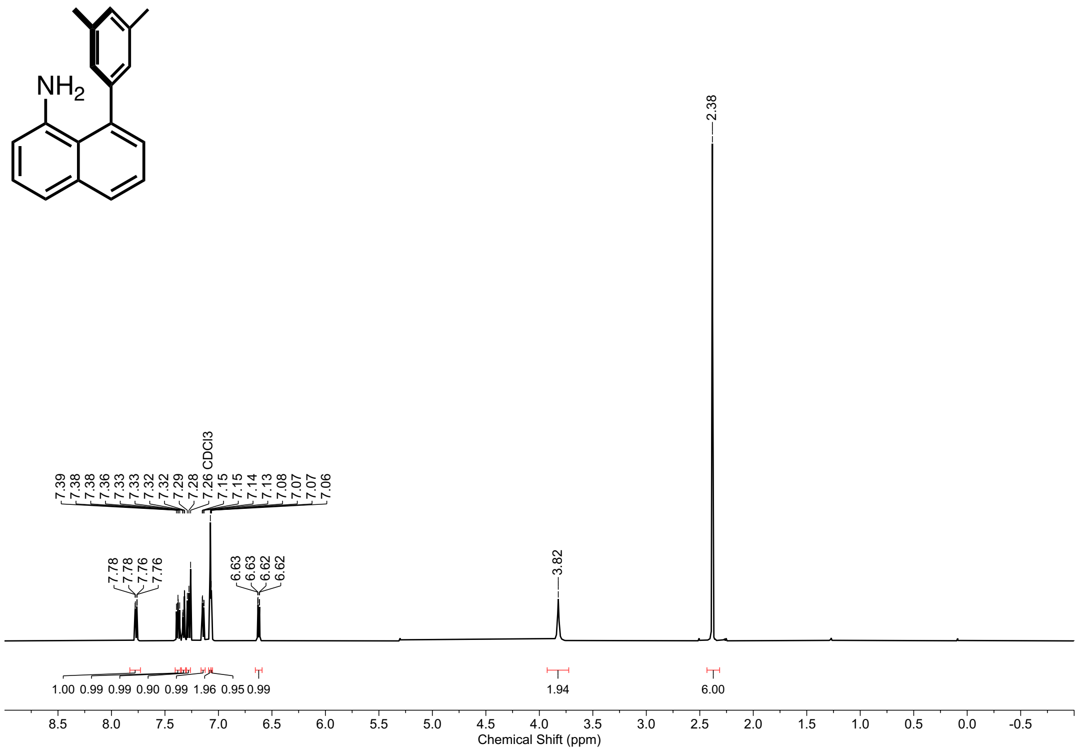

Figure S45: ^1H NMR (500 MHz, $CDCl_3$) spectrum of 8-(3,5-dimethylphenyl)naphthalen-1-amine.

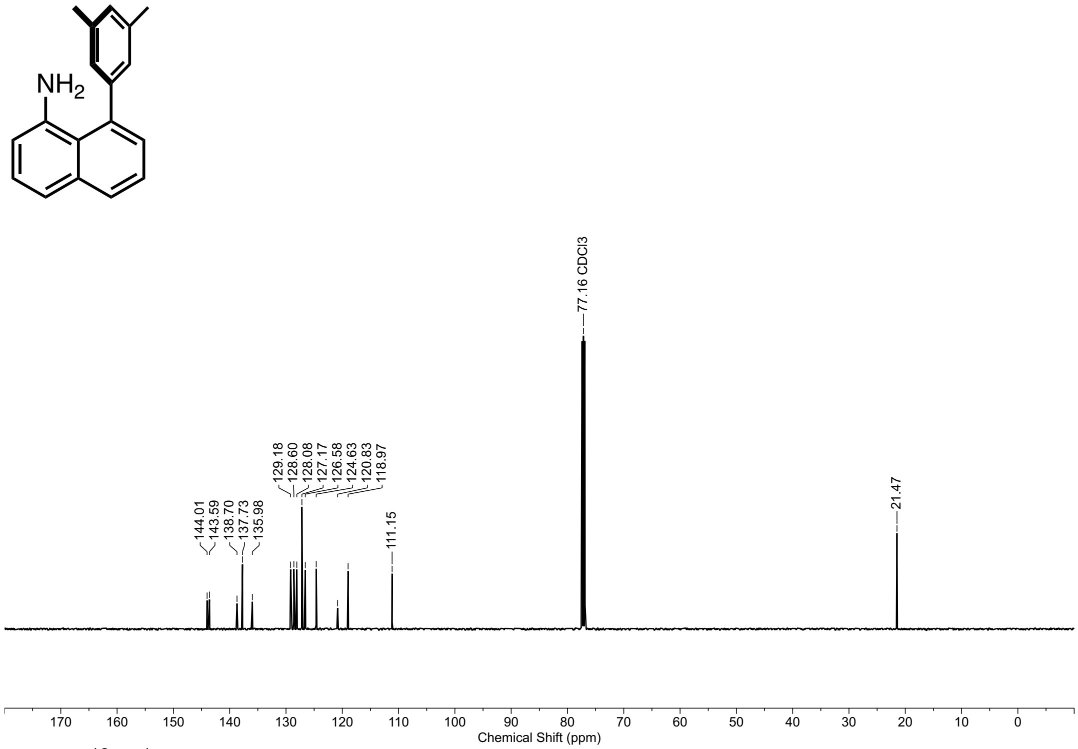

Figure S46: $^{13}C\{^1H\}$ NMR (126 MHz, $CDCl_3$) spectrum of 8-(3,5-dimethylphenyl)naphthalen-1-amine.

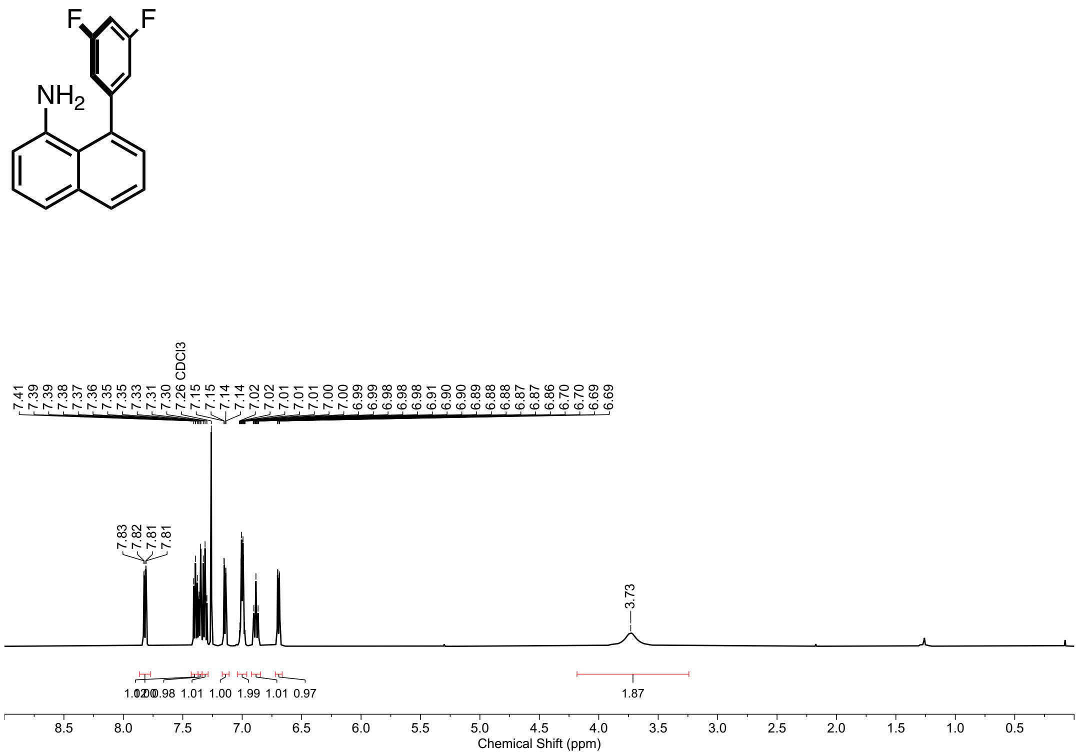

Figure S47: ^{1}H NMR (500 MHz, $CDCl_3$) spectrum of 8-(3,5-difluorophenyl)naphthalen-1-amine.

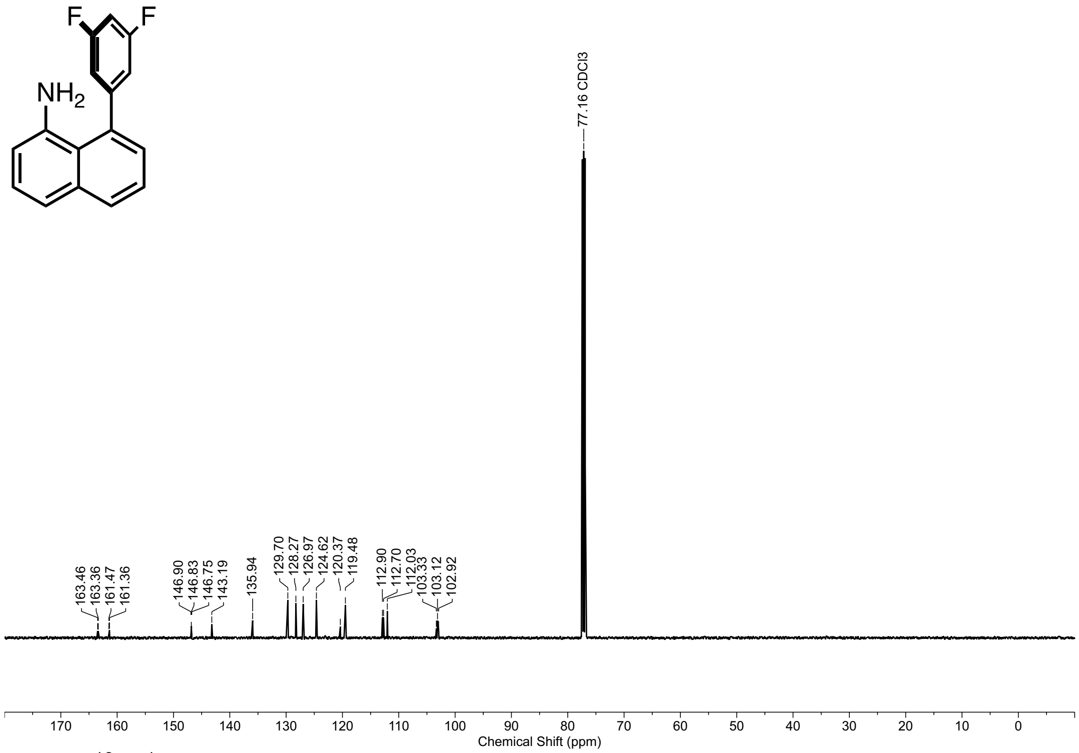

Figure S48: $^{13}C\{^{1}H\}$ NMR (126 MHz, $CDCl_3$) spectrum of 8-(3,5-difluorophenyl)naphthalen-1-amine.

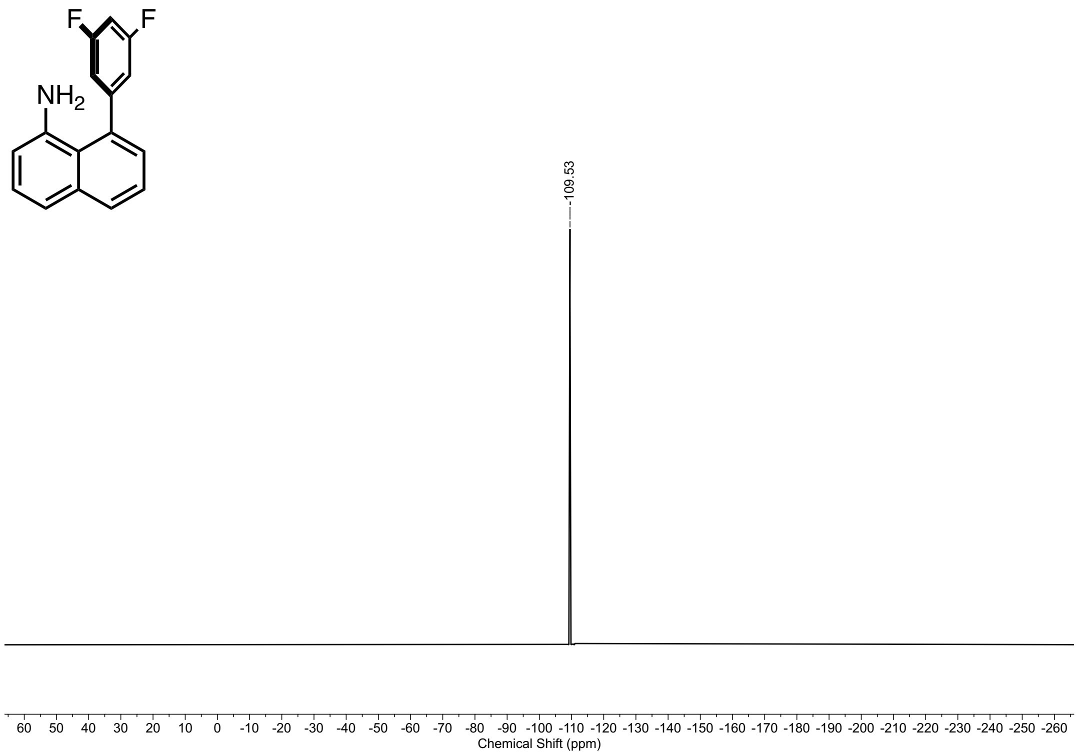

Figure S49: ^{19}F NMR (470 MHz, $CDCl_3$) spectrum of 8-(3,5-difluorophenyl)naphthalen-1-amine.

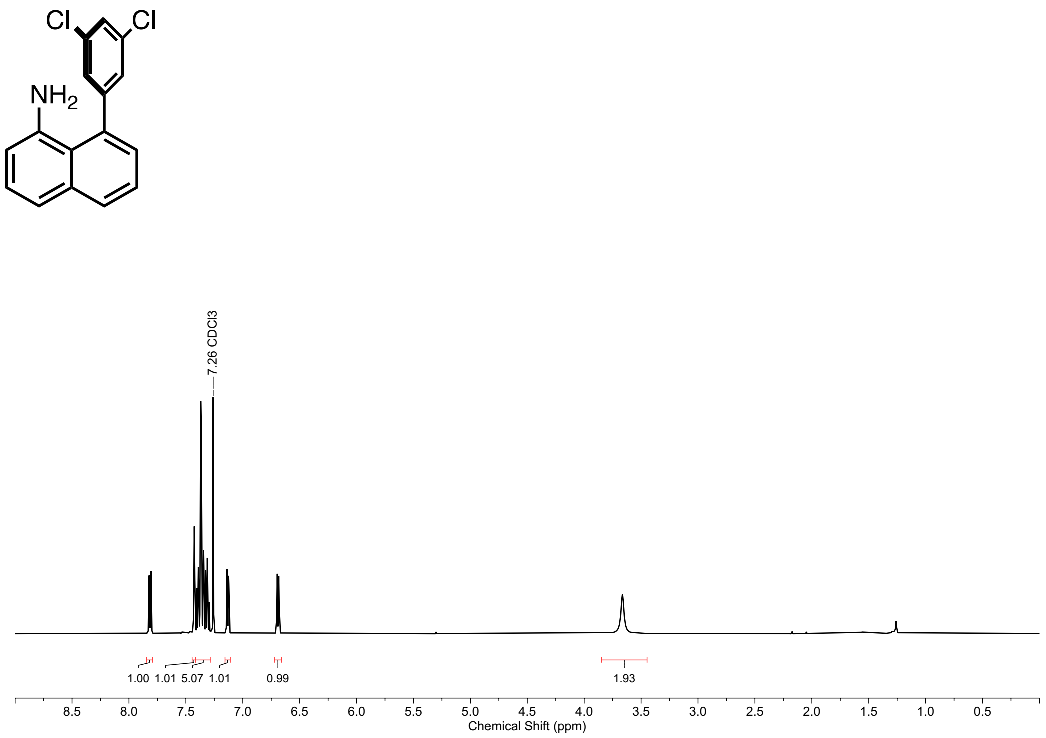

Figure S50: ^{1}H NMR (500 MHz, $CDCl_3$) spectrum of 8-(3,5-dichlorophenyl)naphthalen-1-amine.

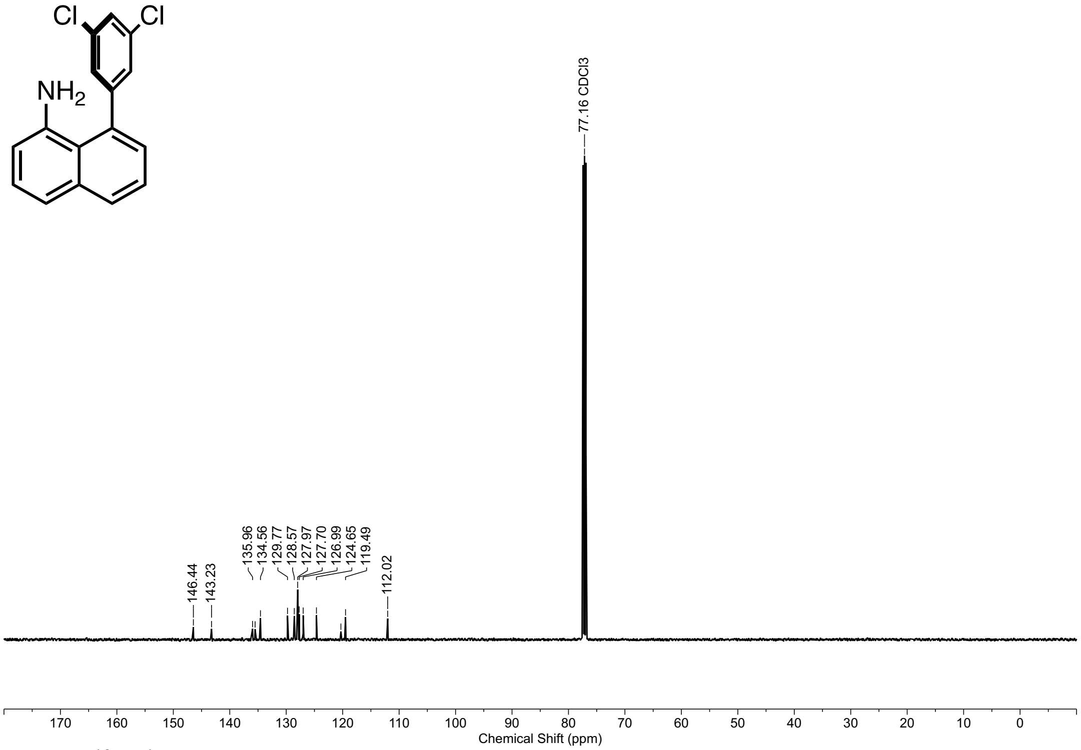

Figure S51: $^{13}C\{^{1}H\}$ NMR (126 MHz, $CDCl_3$) spectrum of 8-(3,5-dichlorophenyl)naphthalen-1-amine.

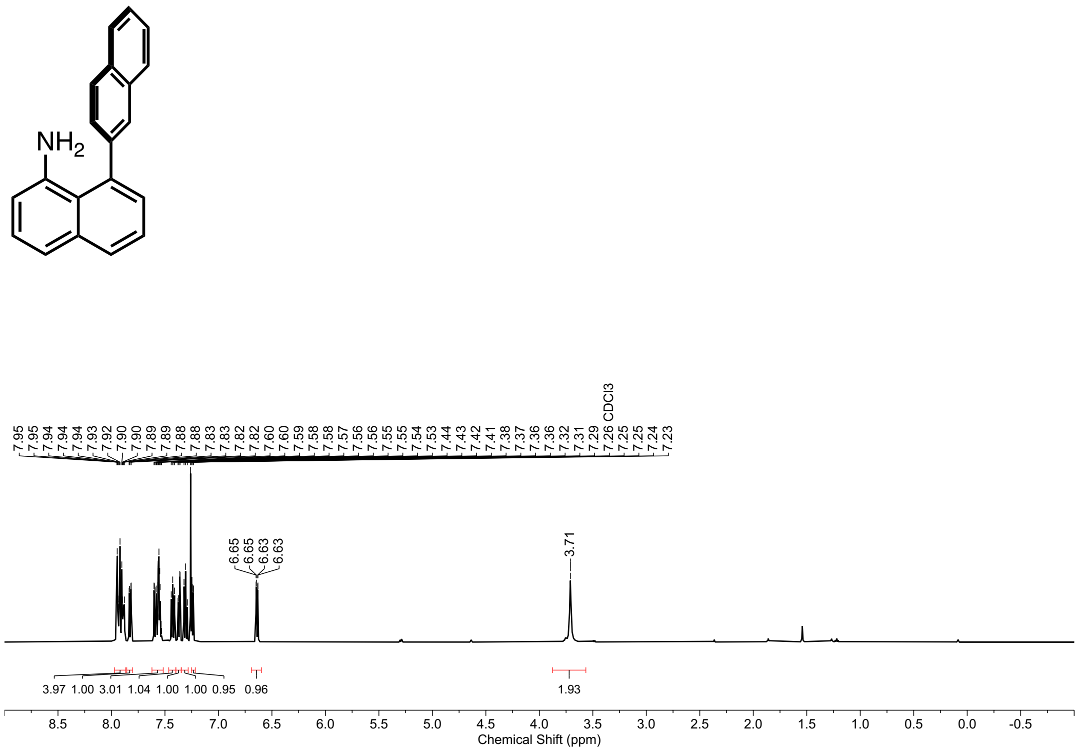

Figure S52: ^{1}H NMR (500 MHz, $CDCl_3$) spectrum of [1,2'-binaphthalen]-8-amine.

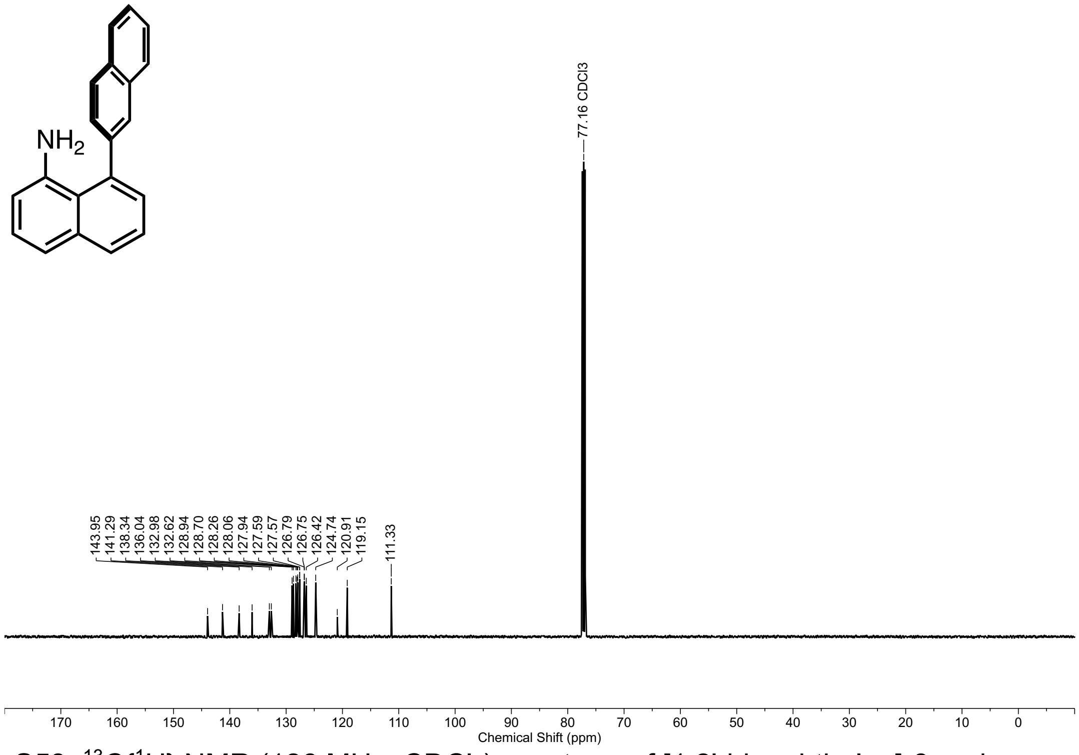

Figure S53: $^{13}C\{^{1}H\}$ NMR (126 MHz, $CDCl_3$) spectrum of [1,2'-binaphthalen]-8-amine.

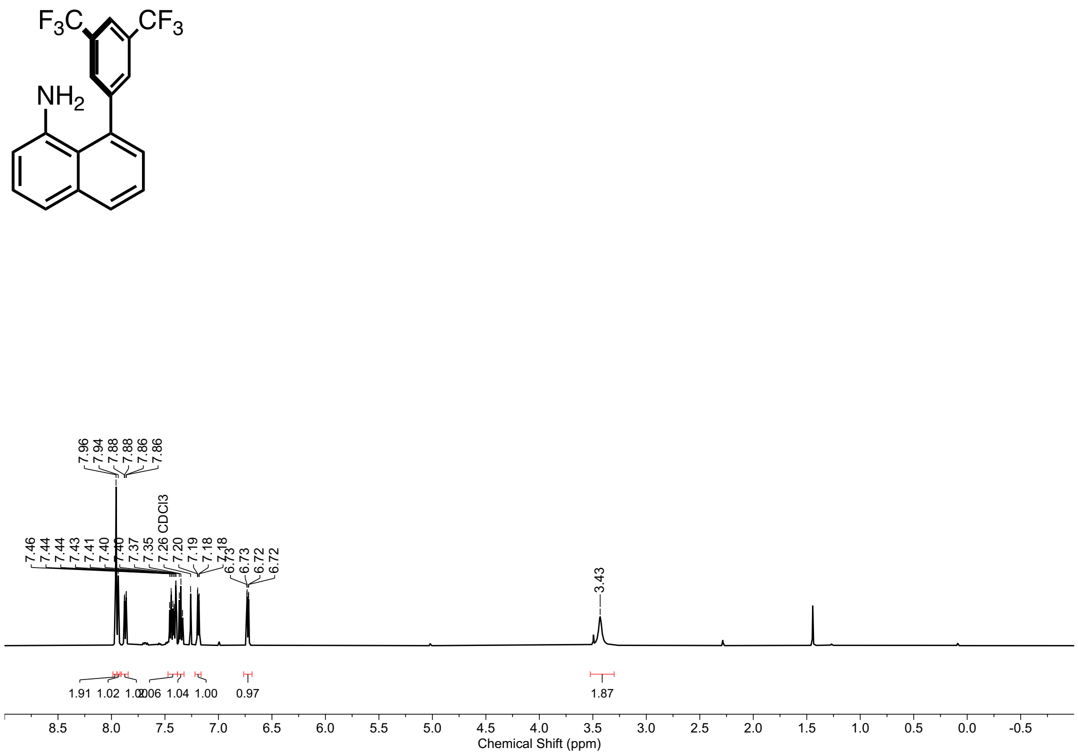

Figure S54: ^{1}H NMR (500 MHz, $CDCl_3$) spectrum of 8-(3,5-bis(trifluoromethyl)phenyl)naphthalen-1-amine.

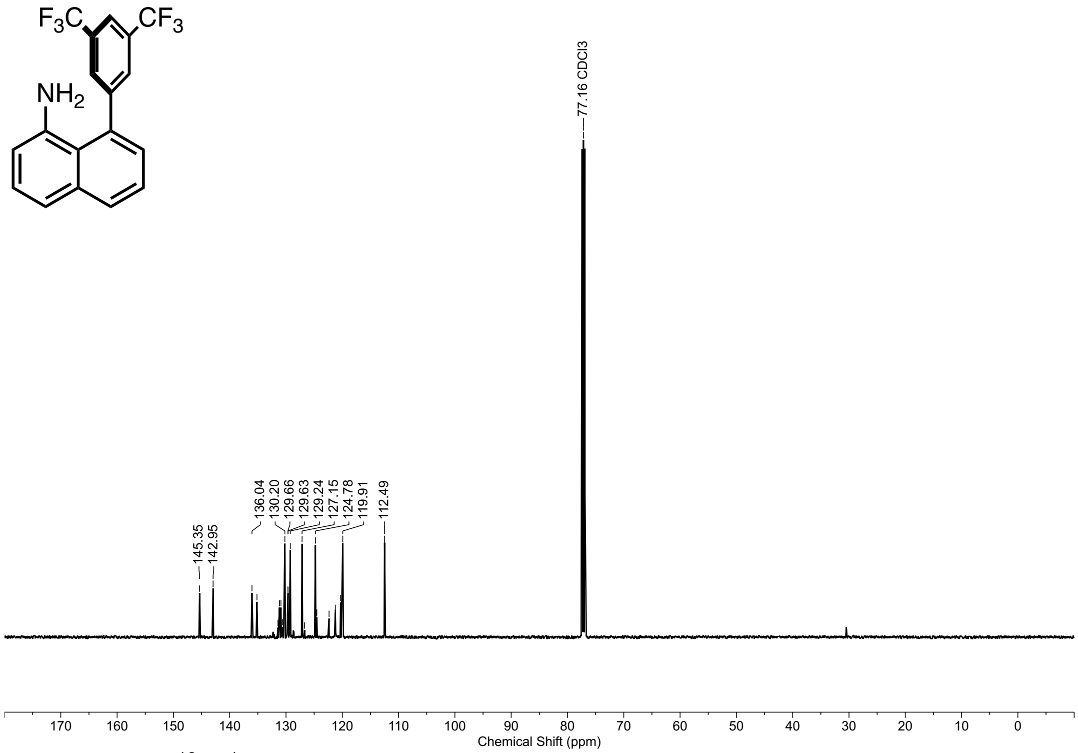

Figure S55: $^{13}C\{^1H\}$ NMR (126 MHz, $CDCl_3$) spectrum of 8-(3,5-bis(trifluoromethyl)phenyl)naphthalen-1-amine.

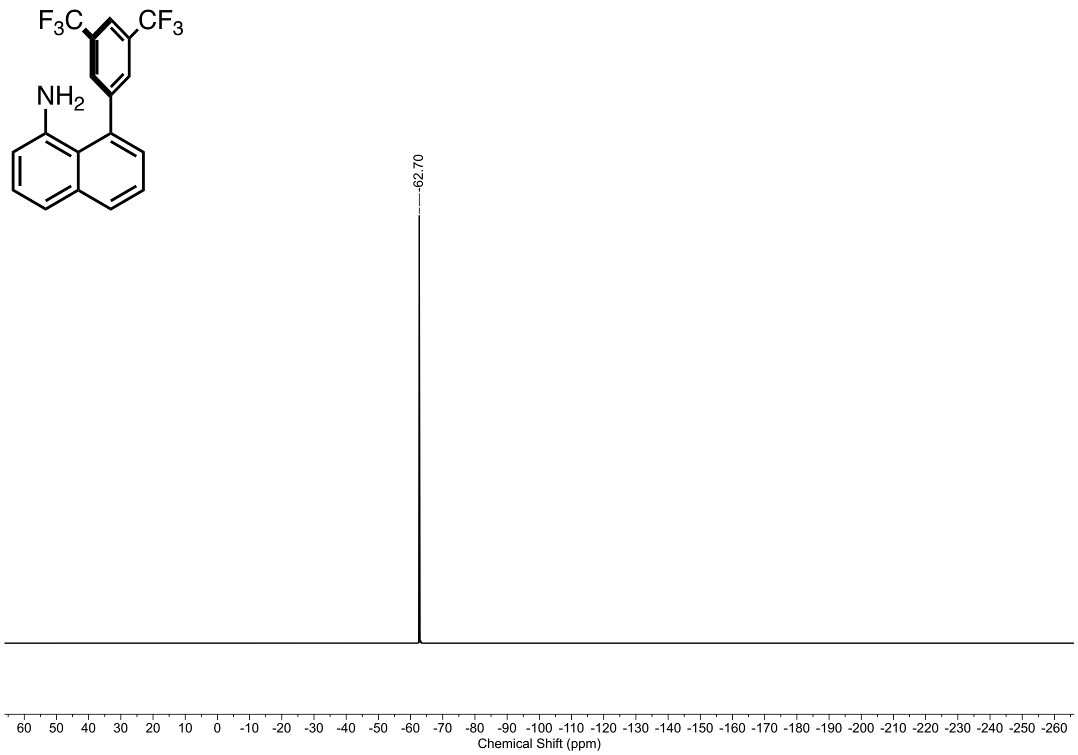

Figure S56: ^{19}F NMR (470 MHz, $CDCl_3$) spectrum of 8-(3,5-bis(trifluoromethyl)phenyl)naphthalen-1-amine.

4.2 *rac*-BDI*H ligands

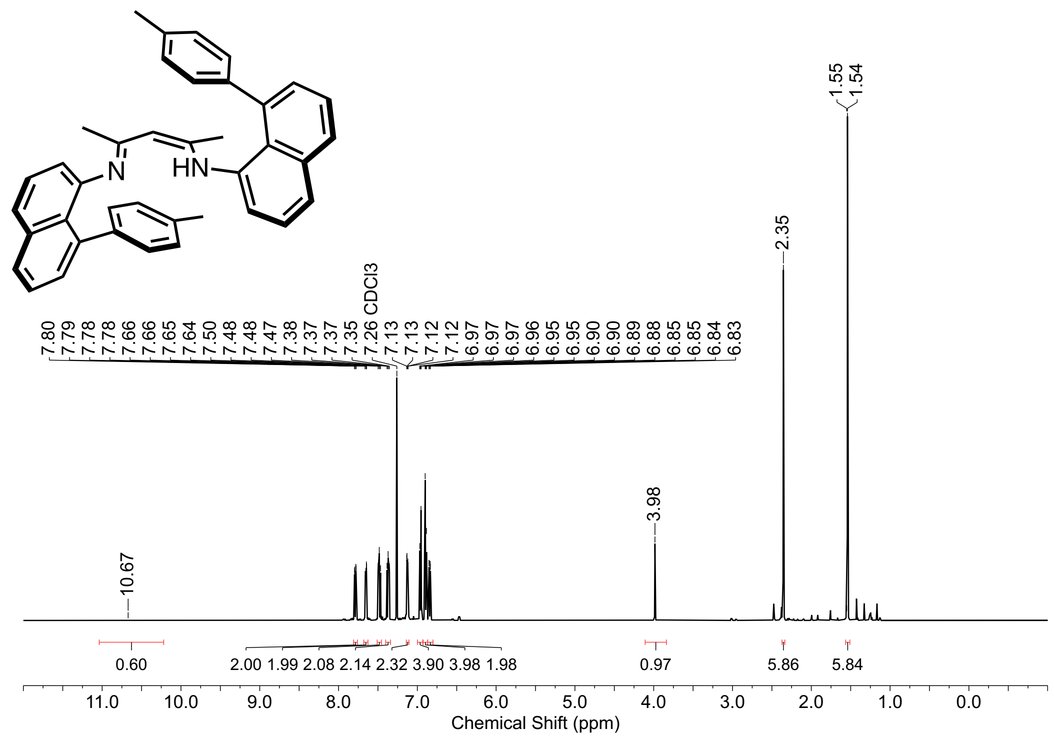

Figure S57: ^{1}H NMR (500 MHz, $CDCl_3$) spectrum of *rac*-$^{4\text{-}Me}$BDI*H.

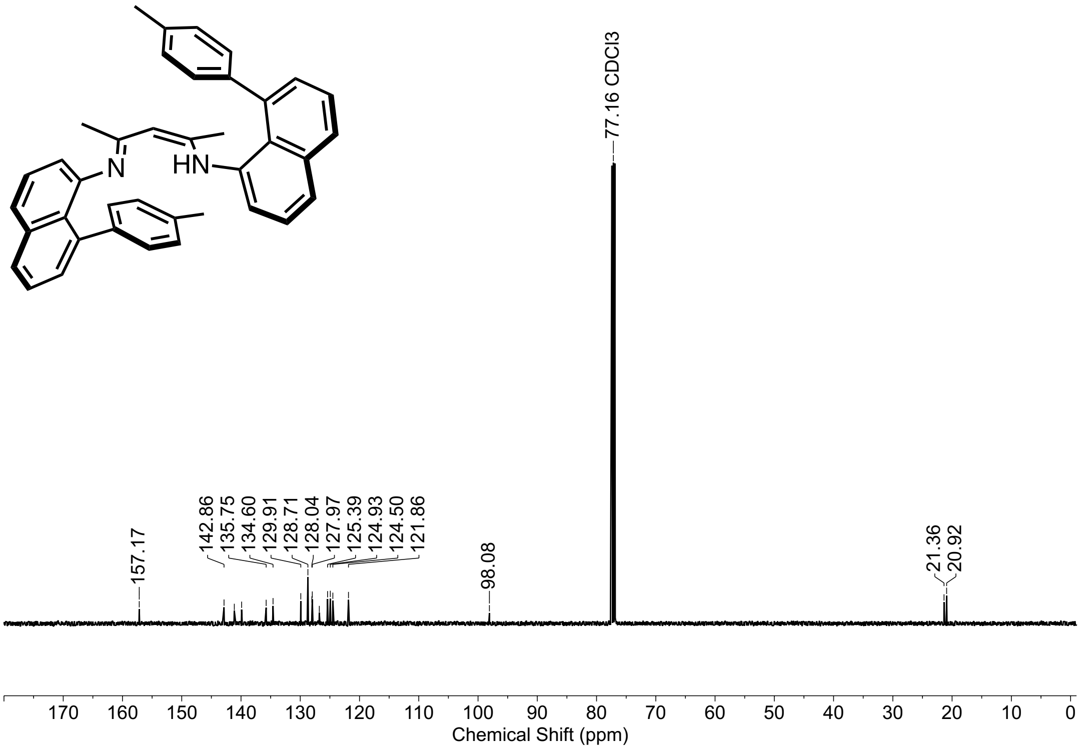

Figure S58: $^{13}C\{^{1}H\}$ NMR (126 MHz, $CDCl_3$) spectrum of *rac*-$^{4\text{-}Me}$BDI*H.

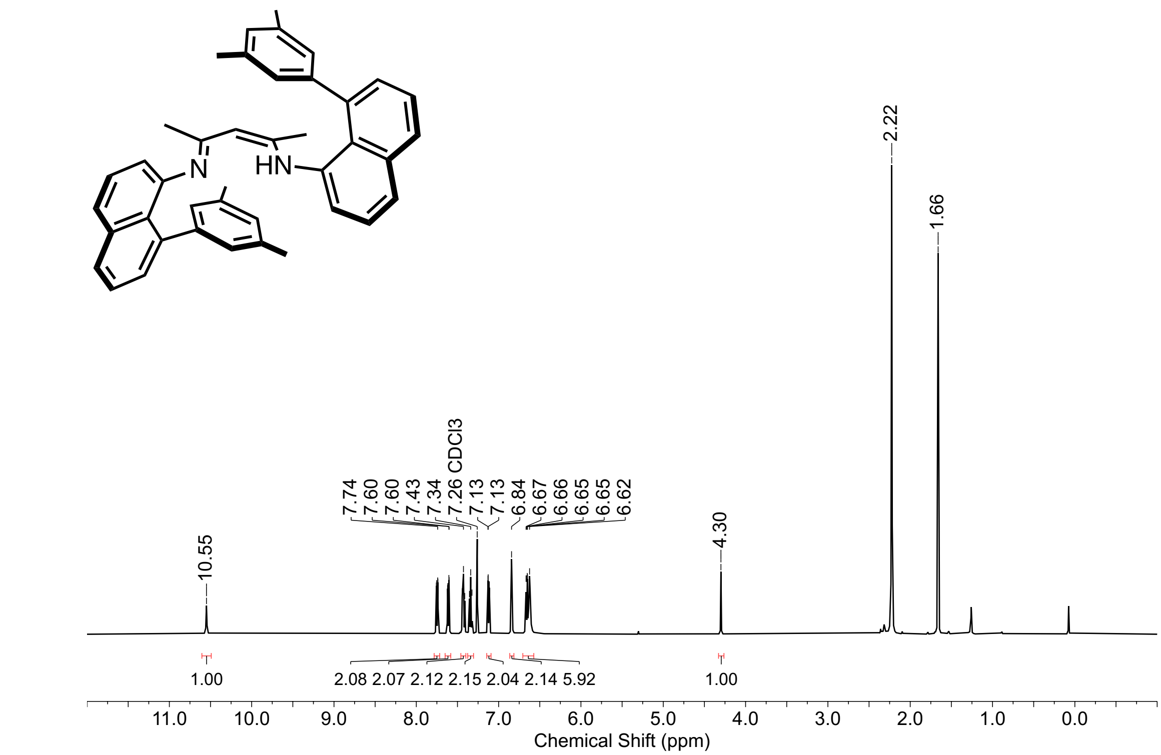

Figure S59: ^{1}H NMR (500 MHz, $CDCl_3$) spectrum of *rac*-$^{3,5\text{-Me}}$BDI*H.

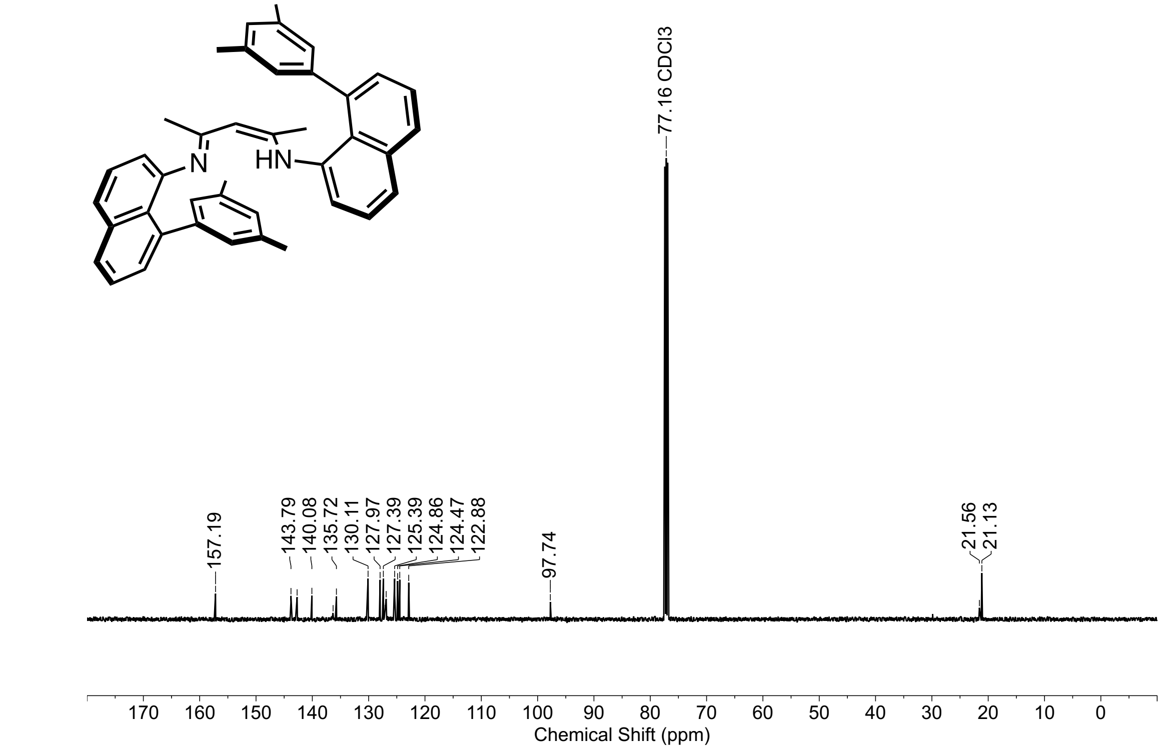

Figure S60: ^{13}C{^{1}H} NMR (126 MHz, $CDCl_3$) spectrum of *rac*-$^{3,5\text{-Me}}$BDI*H.

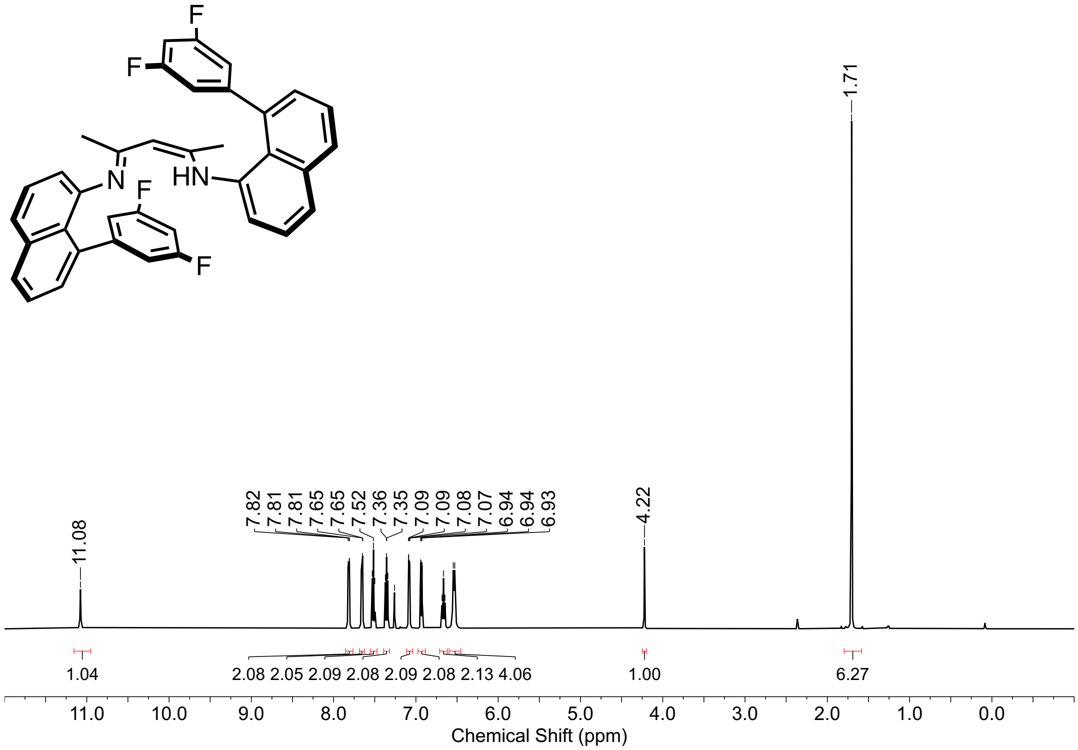

Figure S61: ^{1}H NMR (500 MHz, $CDCl_3$) spectrum of *rac*-$^{3,5\text{-}F}$BDI*H.

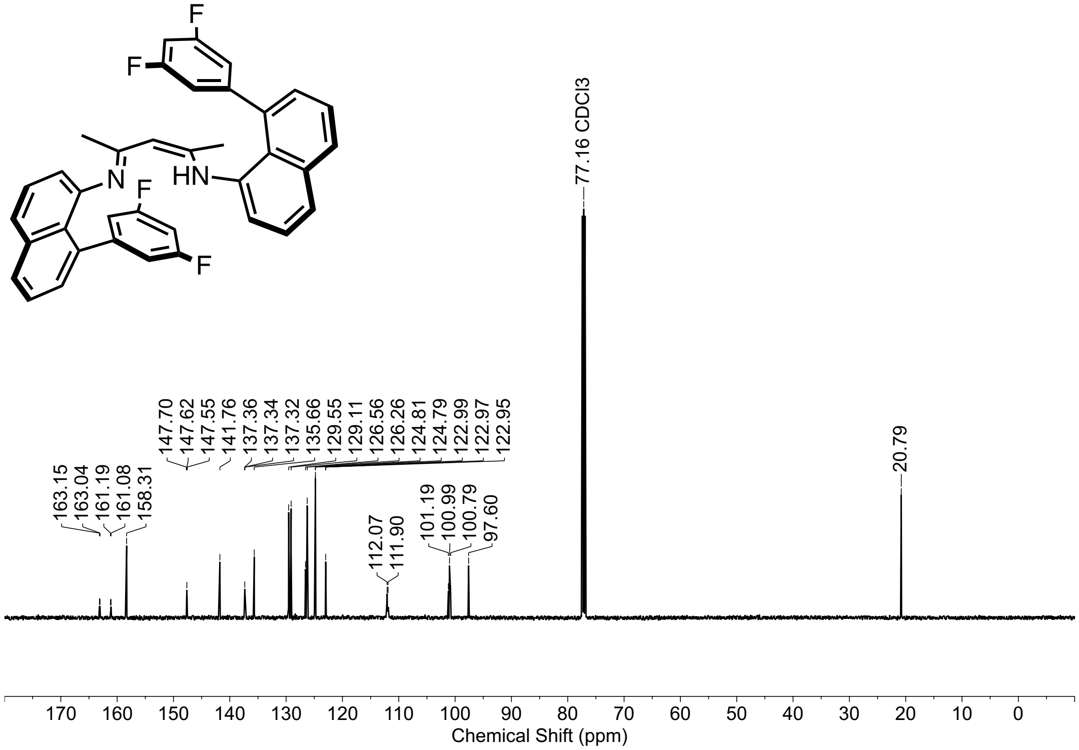

Figure S62: $^{13}C\{^{1}H\}$ NMR (126 MHz, $CDCl_3$) spectrum of *rac*-$^{3,5\text{-}F}$BDI*H.

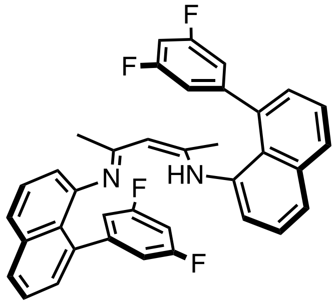

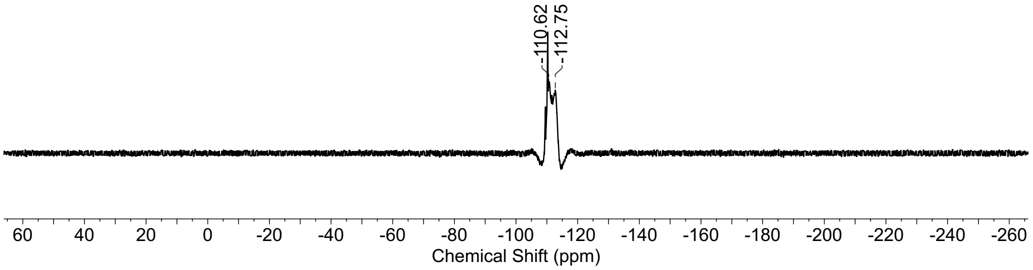

Figure S63: ^{19}F NMR (470 MHz, $CDCl_3$) spectrum of *rac*-$^{3,5\text{-}F}$BDI*H.

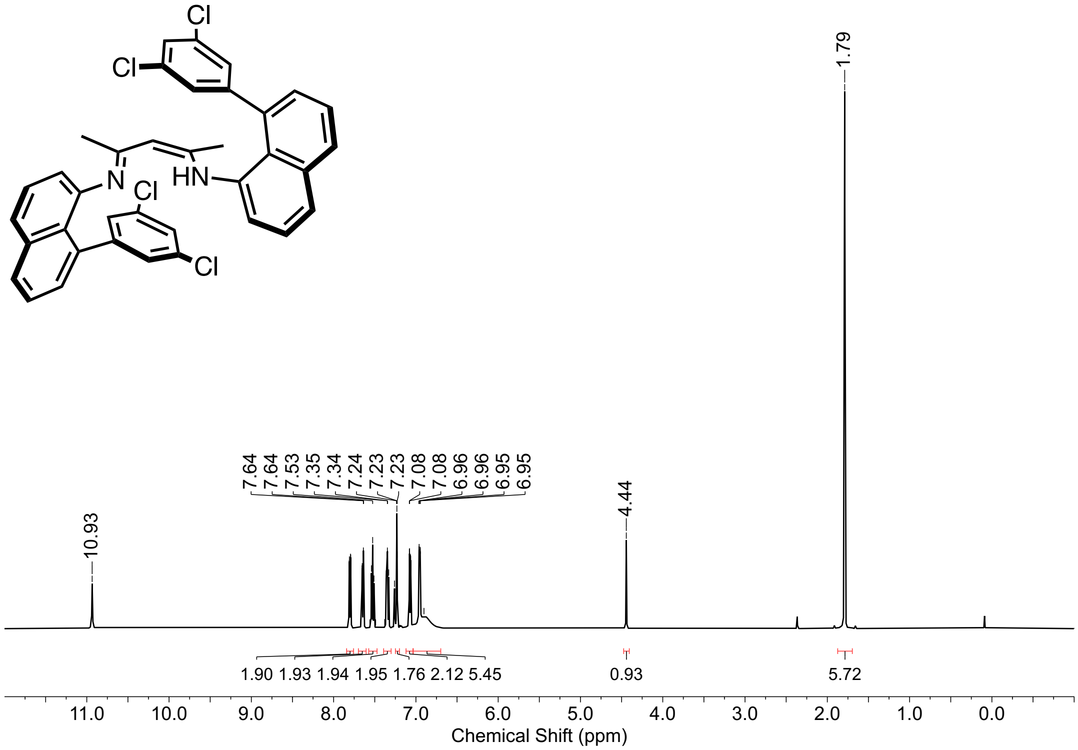

Figure S64: ^{1}H NMR (500 MHz, $CDCl_3$) spectrum of *rac*-$^{3,5\text{-}Cl}$BDI*H.

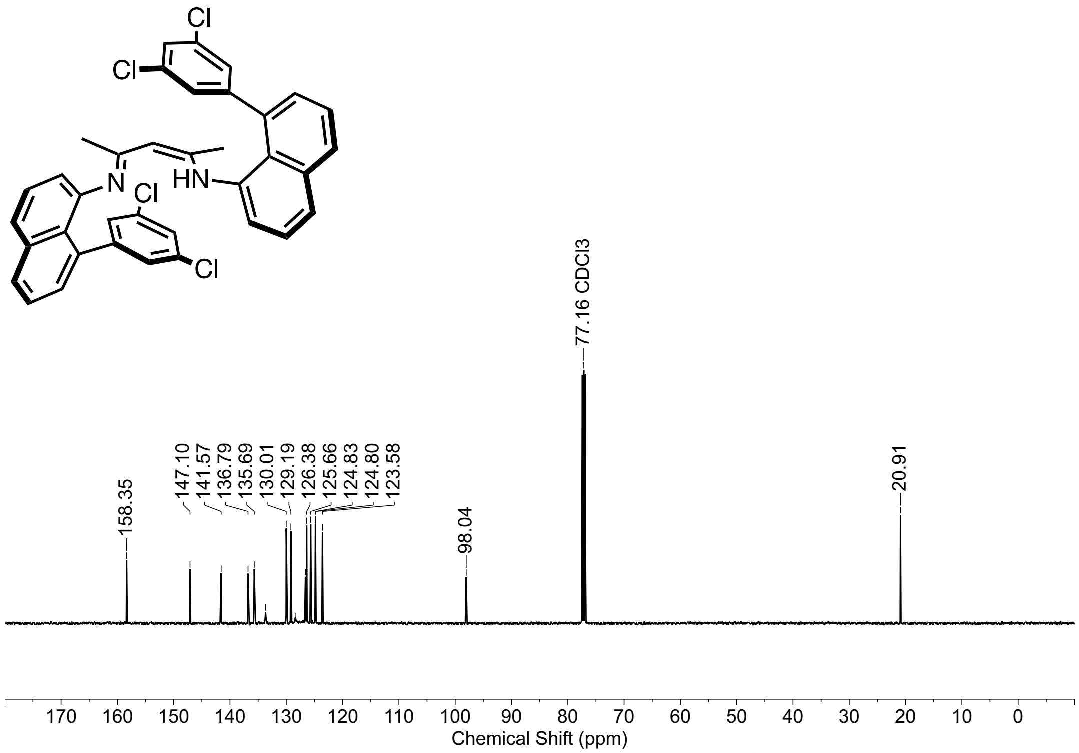

Figure S65: $^{13}C\{^{1}H\}$ NMR (126 MHz, $CDCl_3$) spectrum of *rac*-$^{3,5\text{-}Cl}$BDI*H.

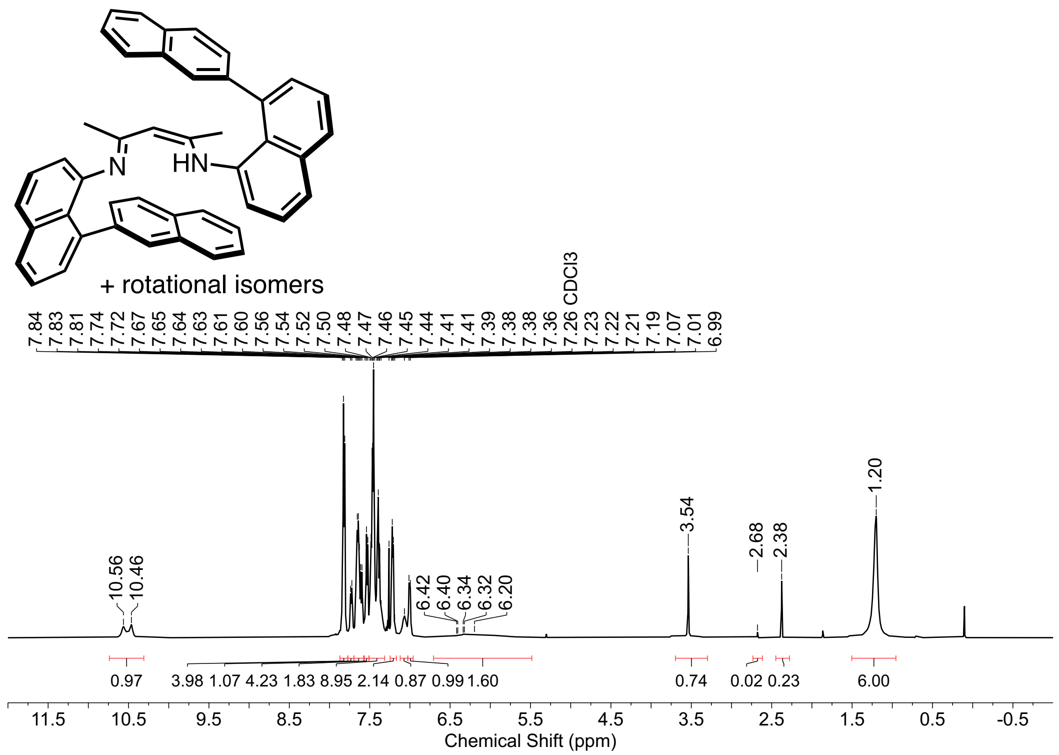

Figure S66: ^{1}H NMR (500 MHz, $CDCl_3$) spectrum of *rac*-$^{\text{2-naphthyl}}$BDI*H.

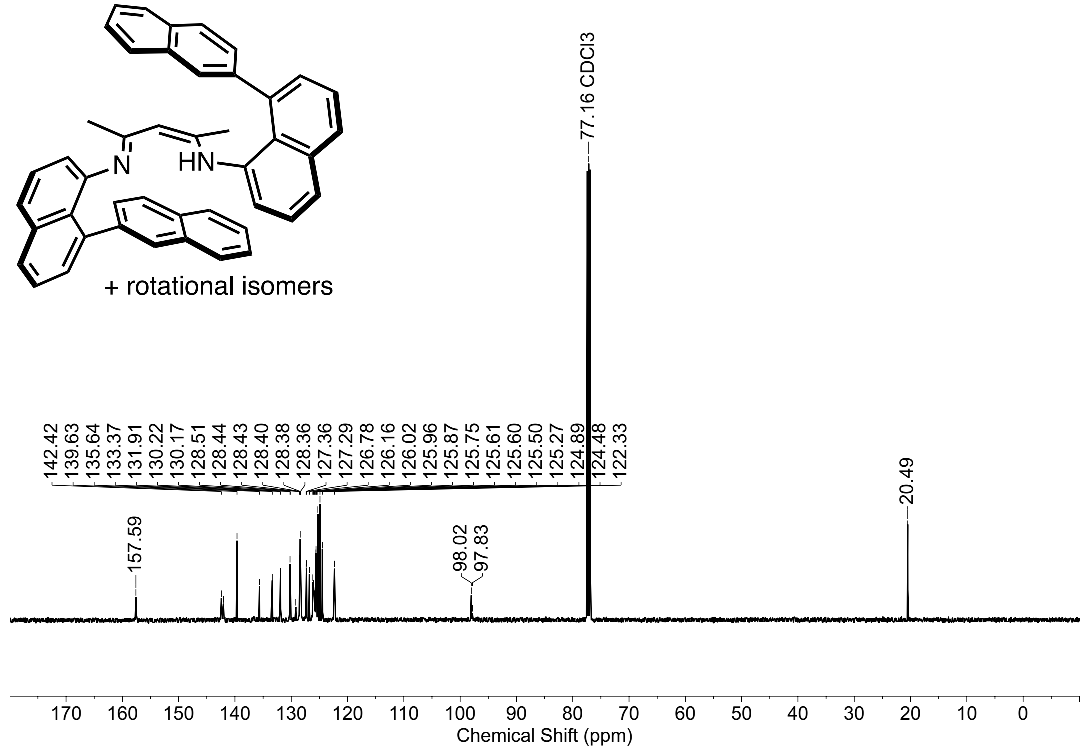

Figure S67: $^{13}C\{^{1}H\}$ NMR (126 MHz, $CDCl_3$) spectrum of *rac*-$^{\text{2-naphthyl}}$BDI*H.

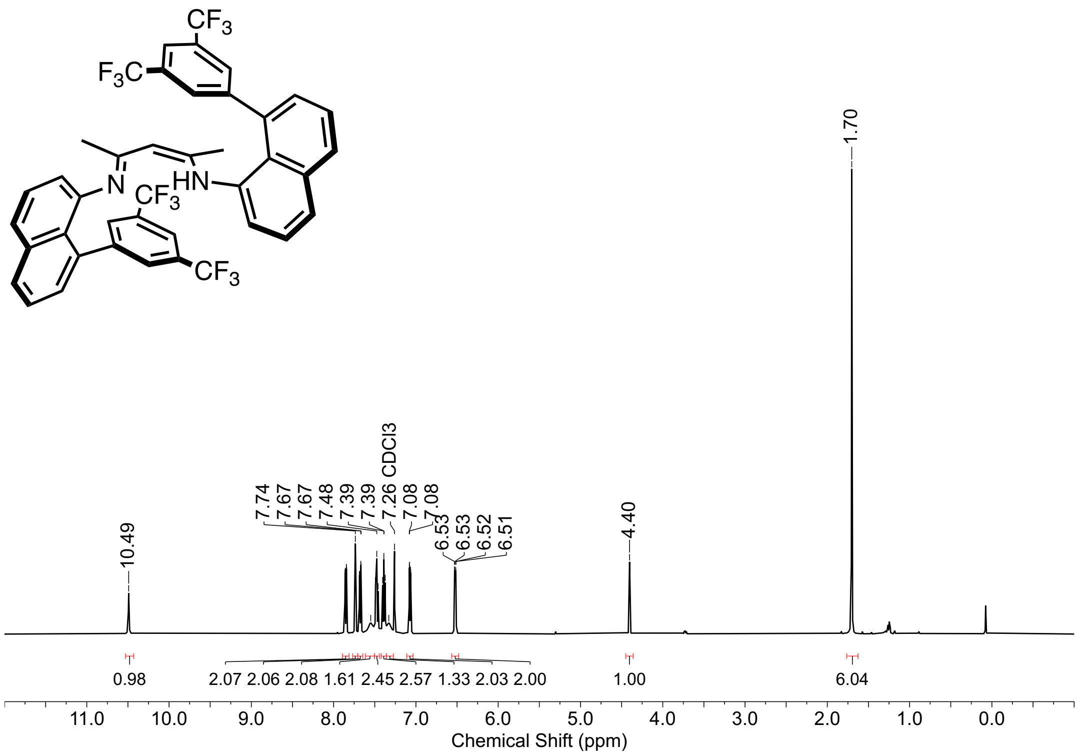

Figure S68: ^{1}H NMR (500 MHz, $CDCl_3$) spectrum of *rac*-$^{3,5\text{-}CF3}$BDI*H.

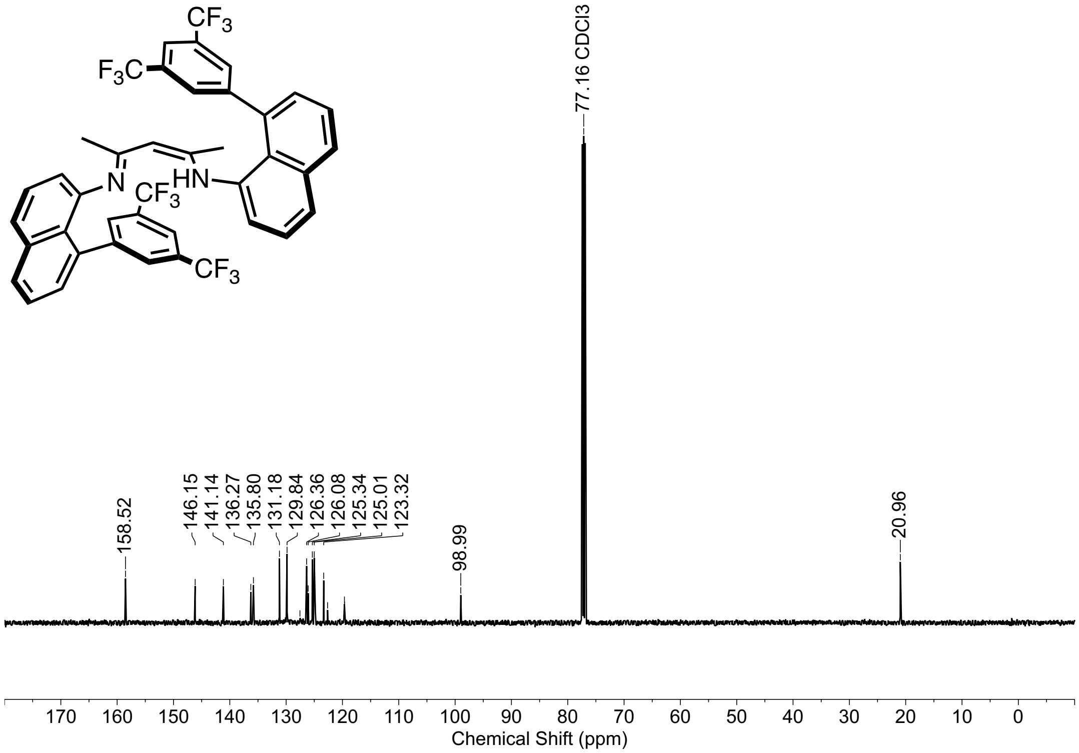

Figure S69: ^{13}C{^{1}H} NMR (126 MHz, $CDCl_3$) spectrum of *rac*-$^{3,5\text{-}CF3}$BDI*H.

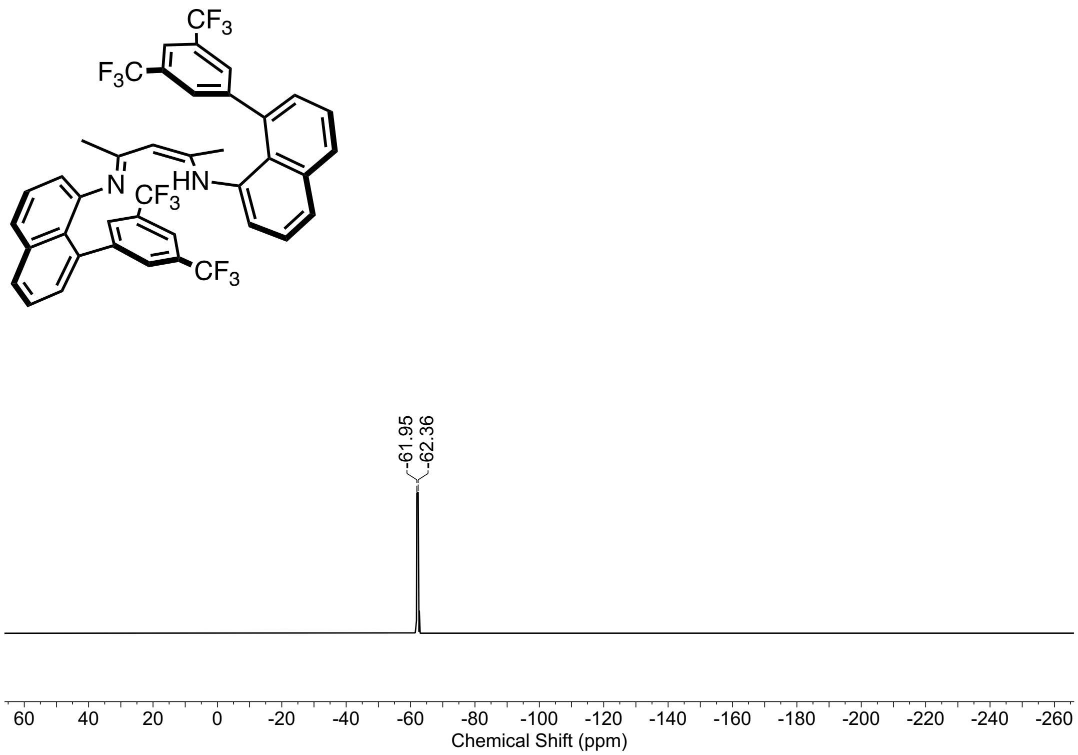

Figure S70: ^{19}F NMR (470 MHz, $CDCl_3$) spectrum of *rac*-$^{3,5\text{-}CF3}$BDI*H.

4.3 *rac*-(^{Ar}BDI*)Zn(O^{i}Pr) complexes

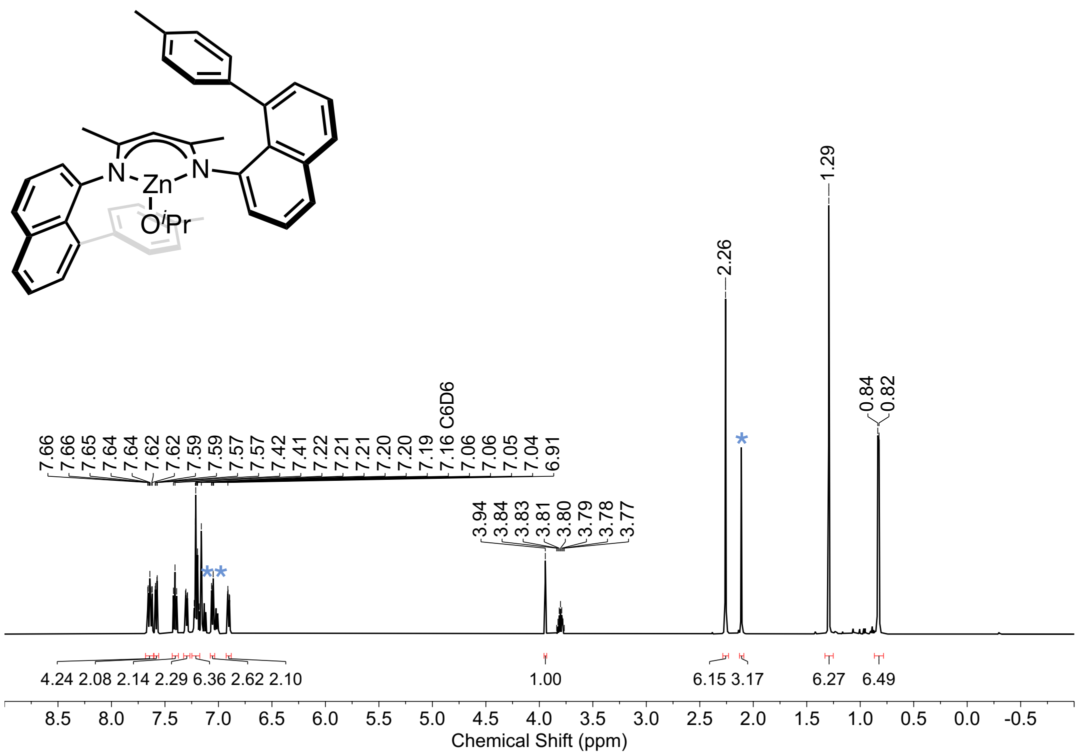

Figure S71: ^{1}H NMR (500 MHz, C_6D_6) spectrum of *rac*-($^{4\text{-Me}}$BDI*)ZnO^{i}Pr. *Residual PhMe.

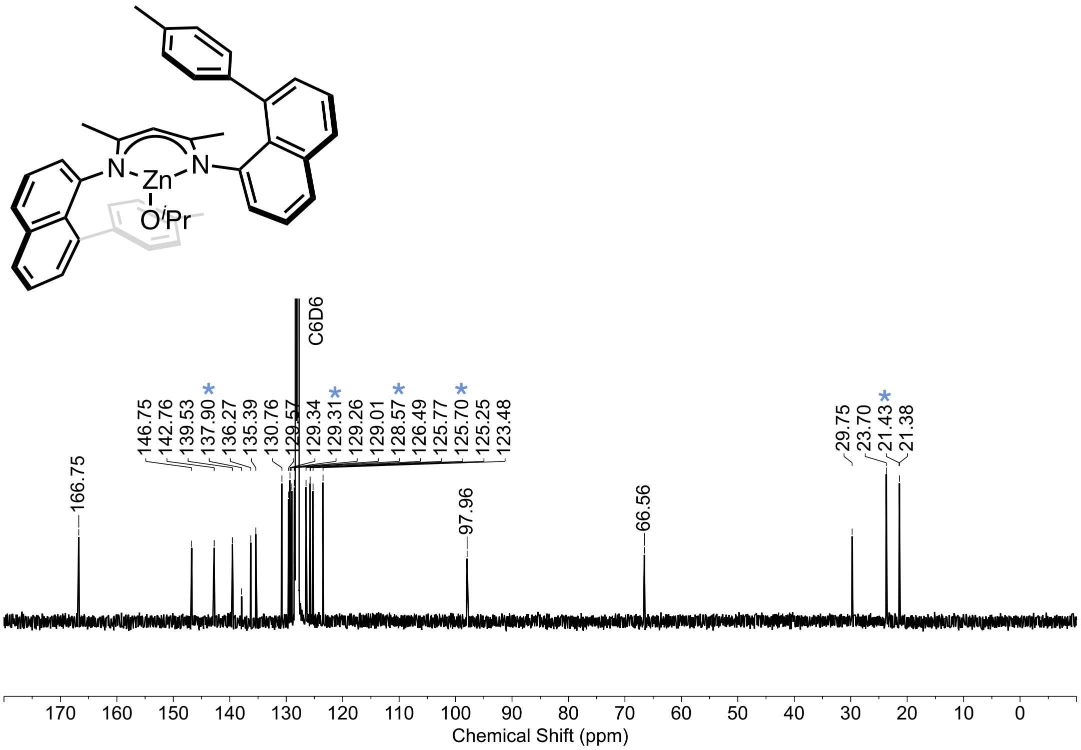

Figure S72: ^{13}C{^{1}H} NMR (126 MHz, C_6D_6) spectrum of *rac*-($^{4\text{-Me}}$BDI*)ZnO^{i}Pr. *Residual PhMe.

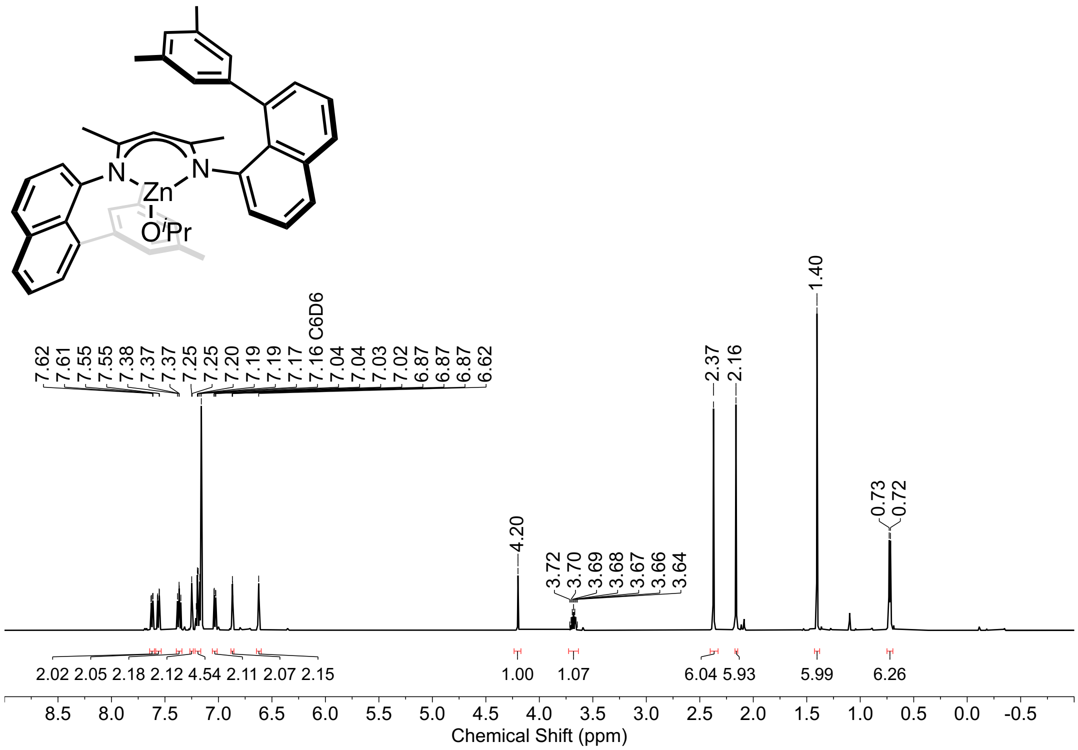

Figure S73: ^{1}H NMR (500 MHz, C_6D_6) spectrum of *rac*-($^{3,5\text{-Me}}$BDI*)ZnO^{i}Pr.

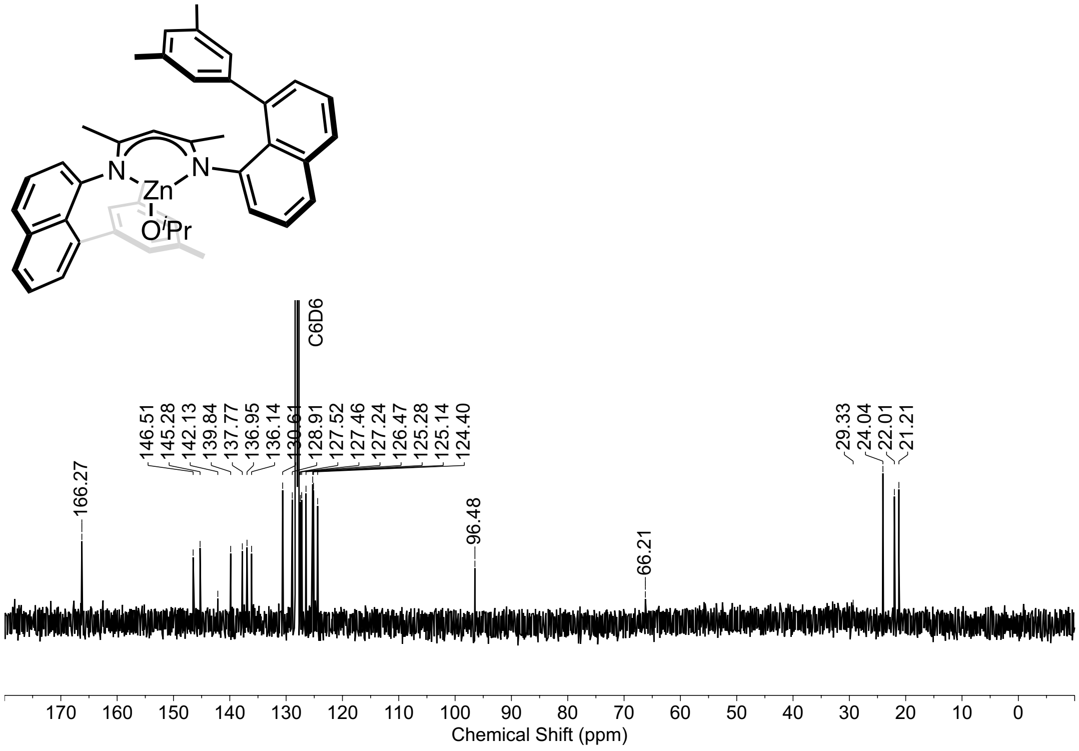

Figure S74: $^{13}C\{^1H\}$ NMR (126 MHz, C_6D_6) spectrum of *rac*-($^{3,5\text{-Me}}$BDI*)ZnO^{i}Pr.

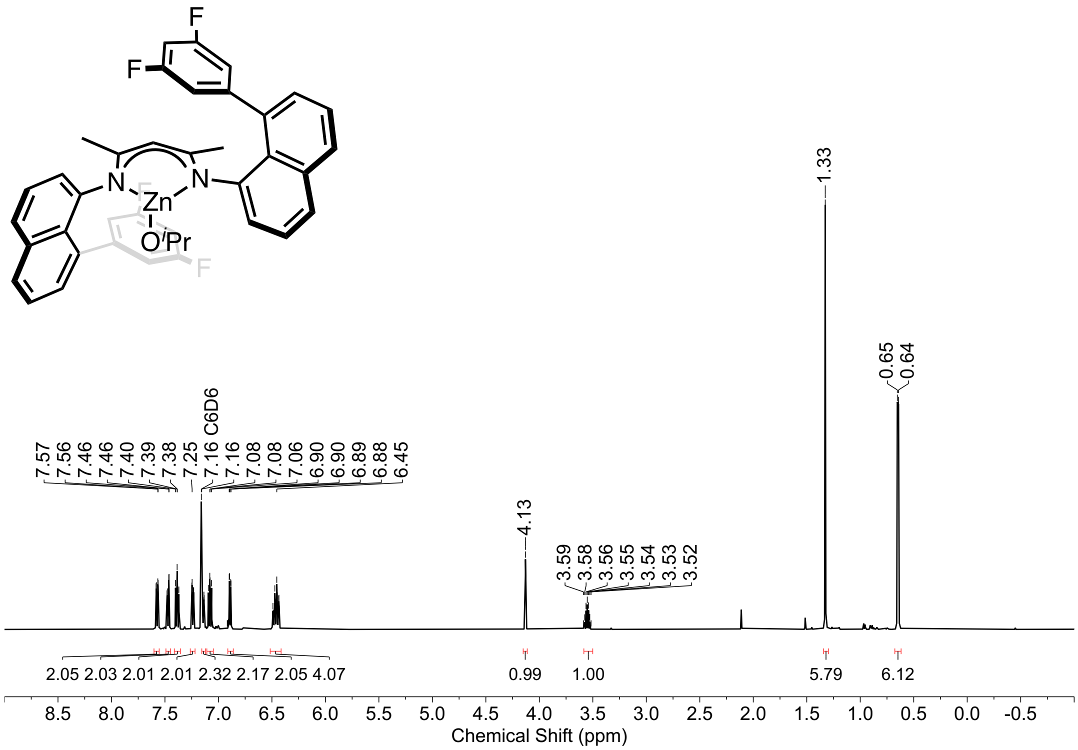

Figure S75: ^{1}H NMR (500 MHz, C_6D_6) spectrum of *rac*-($^{3,5\text{-}F}$BDI*)ZnO^{i}Pr.

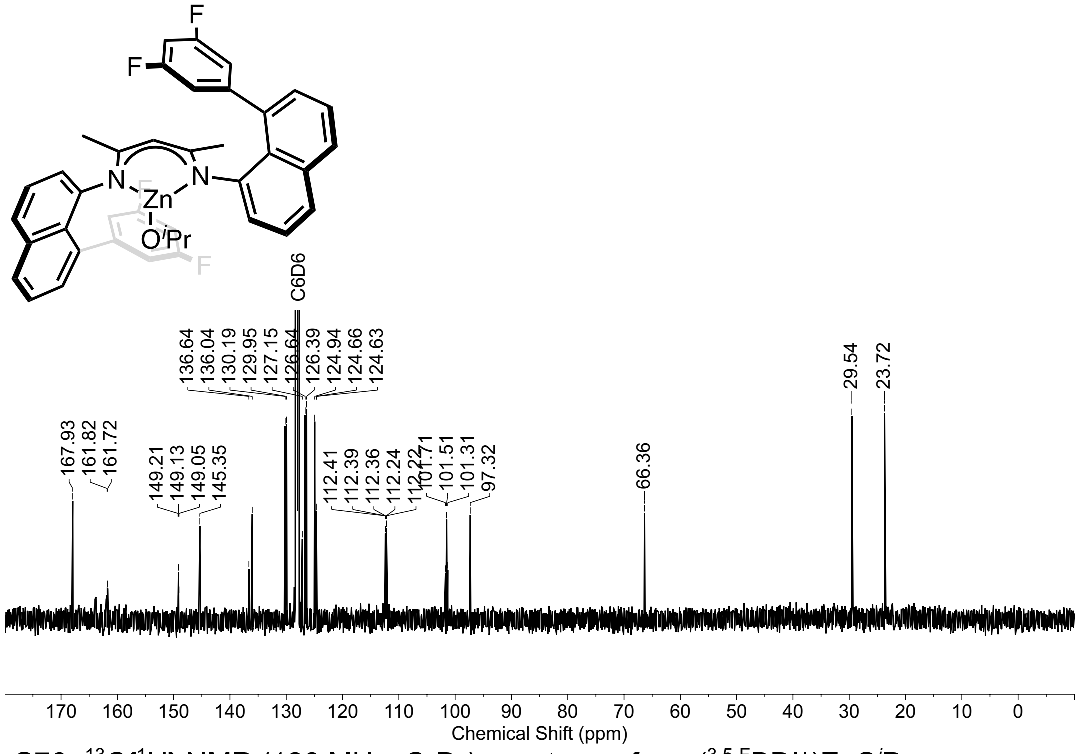

Figure S76: $^{13}C\{^{1}H\}$ NMR (126 MHz, C_6D_6) spectrum of *rac*-($^{3,5\text{-}F}$BDI*)ZnO^{i}Pr.

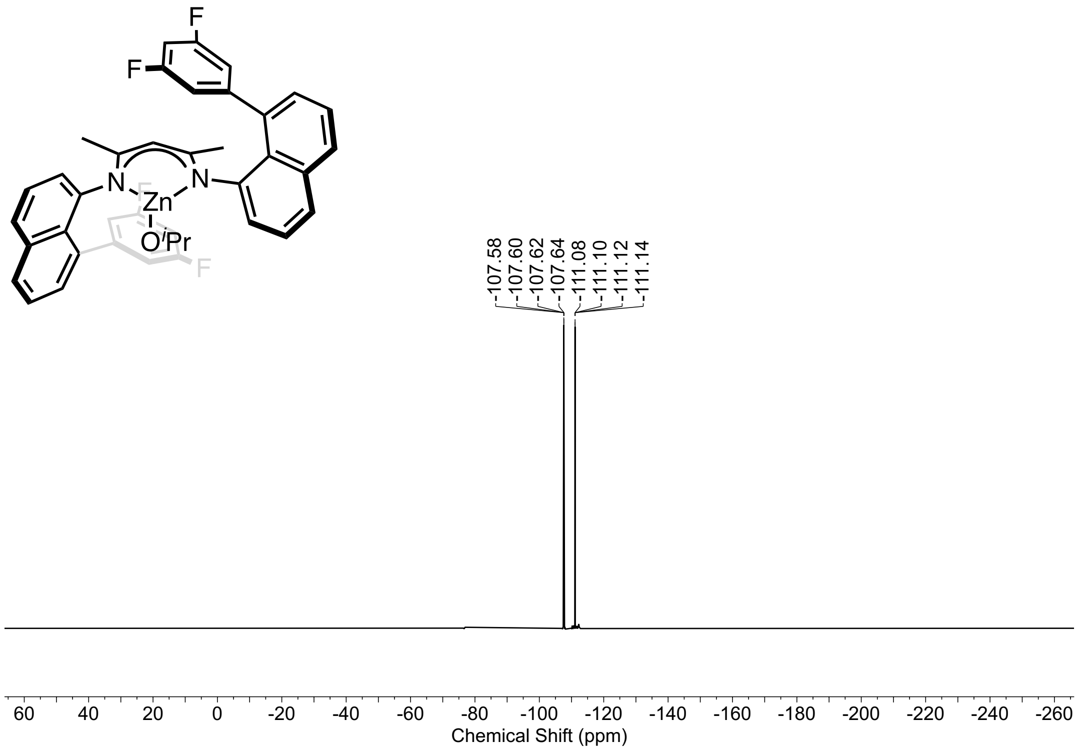

Figure S77: ^{19}F NMR (470 MHz, C_6D_6) spectrum of *rac*-($^{3,5\text{-}F}$BDI*)ZnO^{i}Pr.

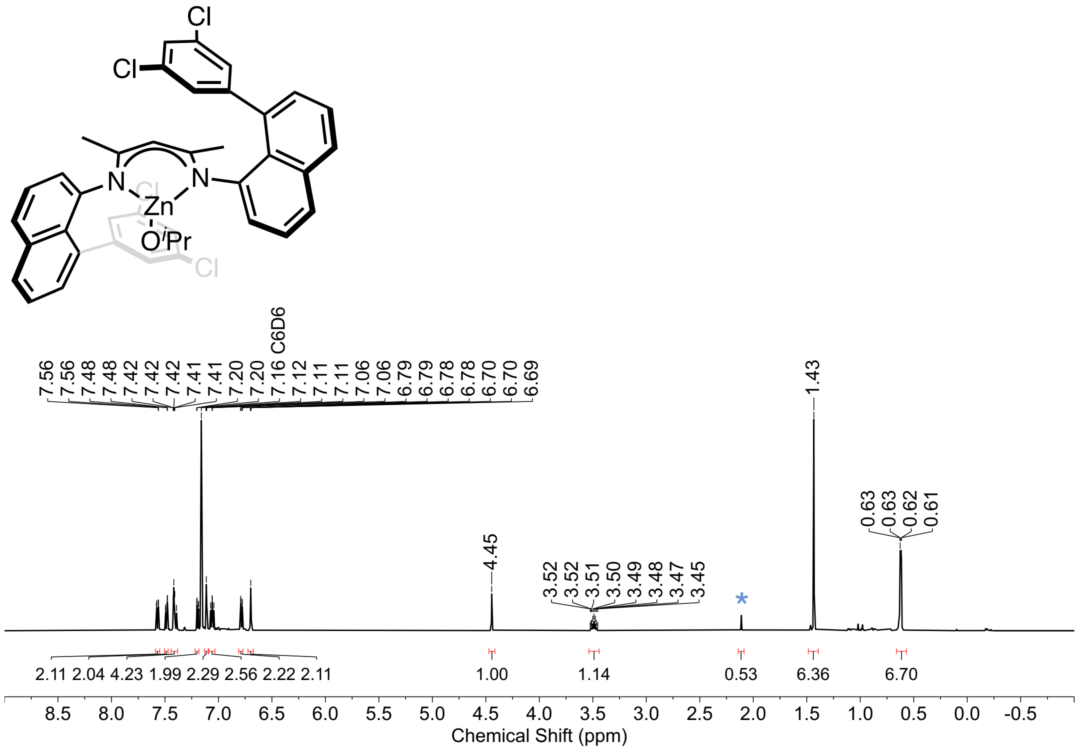

Figure S78: ^{1}H NMR (500 MHz, C_6D_6) spectrum of *rac*-($^{3,5\text{-Cl}}$BDI*)ZnO^{i}Pr. *Residual PhMe.

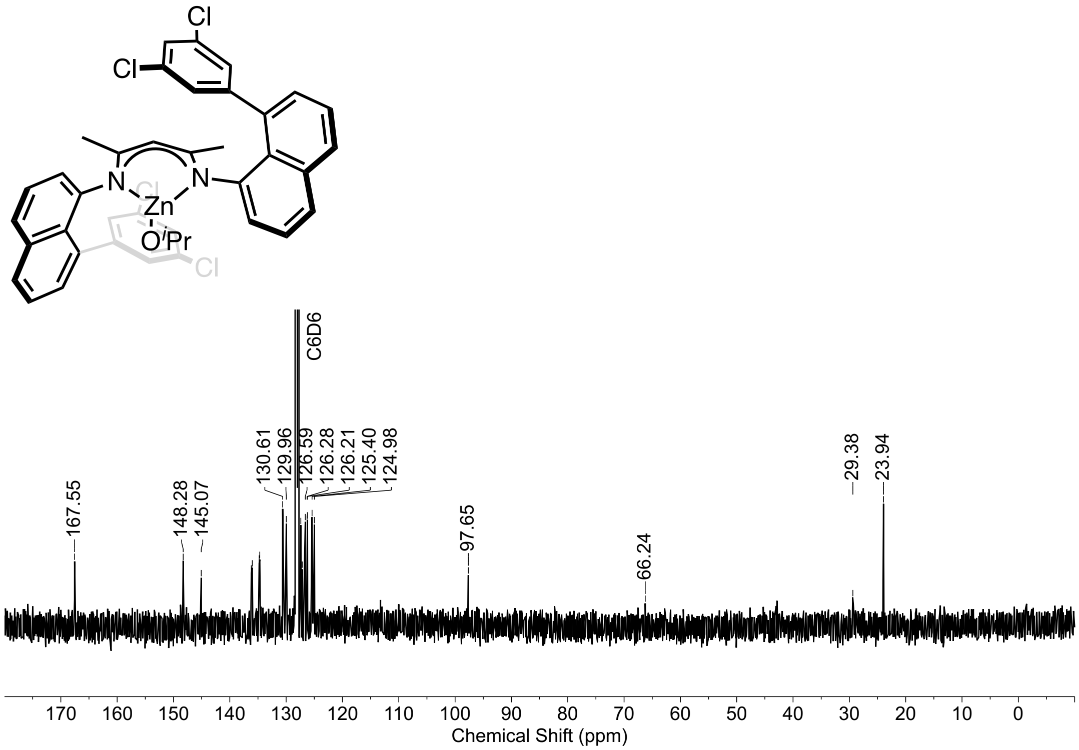

Figure S79: ^{13}C{^{1}H} NMR (126 MHz, C_6D_6) spectrum of *rac*-($^{3,5\text{-Cl}}$BDI*)ZnO^{i}Pr.

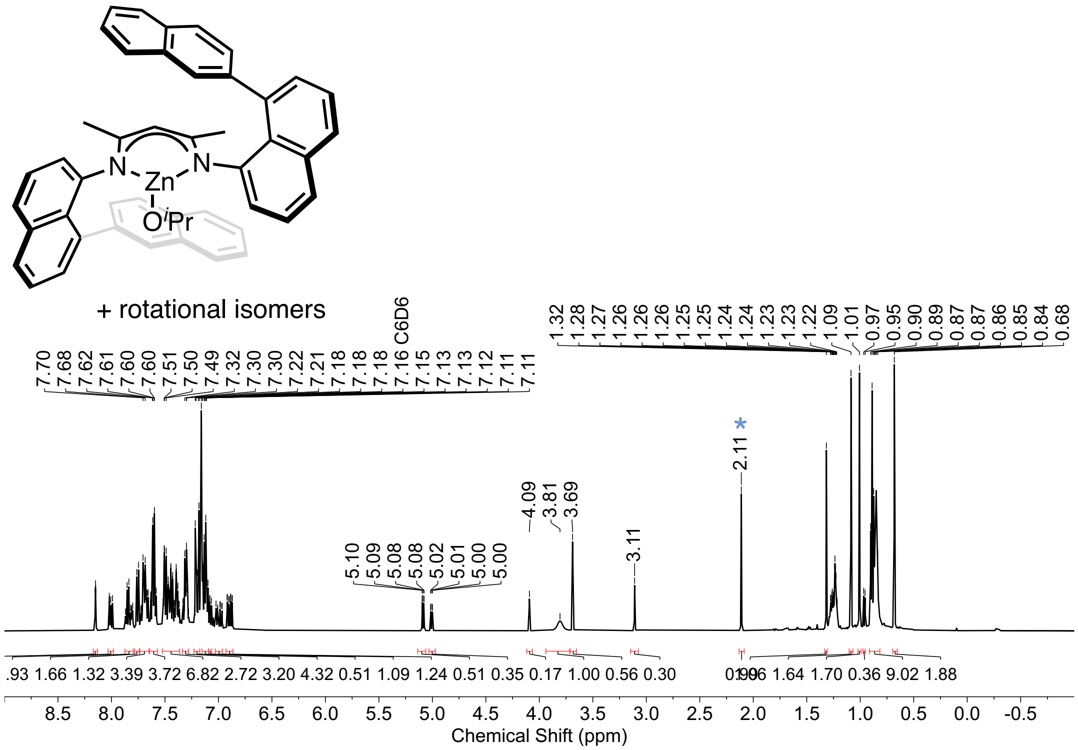

Figure S80: ^{1}H NMR (500 MHz, C_6D_6) spectrum of *rac*-($^{2\text{-naphthyl}}$BDI*)ZnO^{i}Pr. *Residual PhMe.

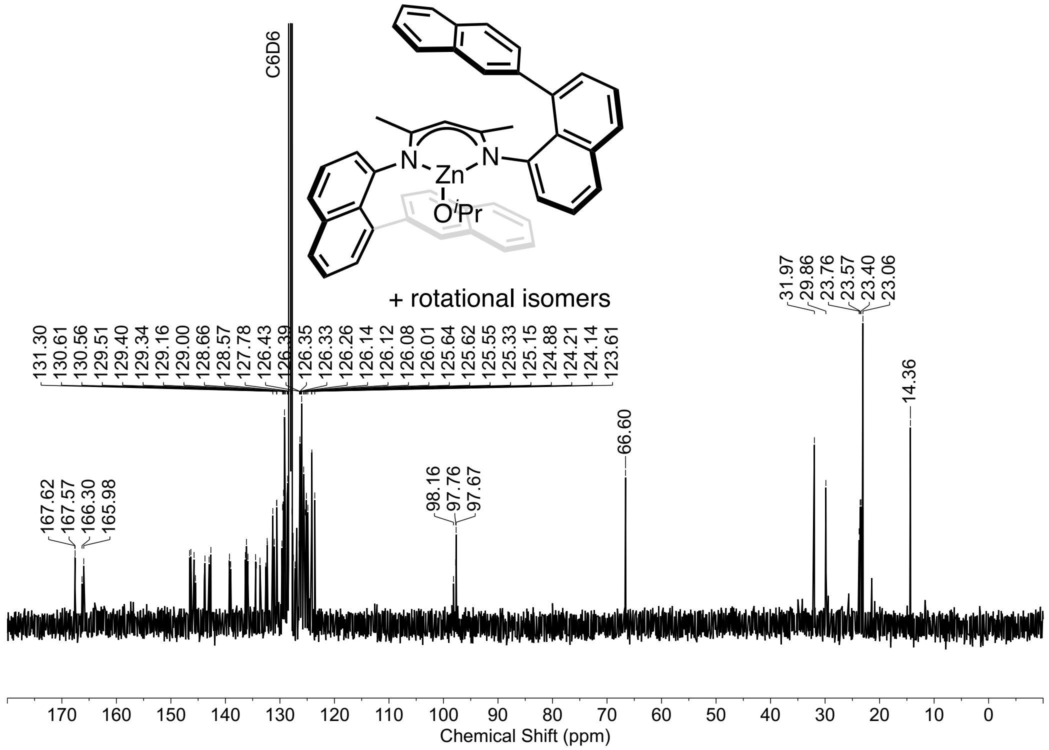

Figure S81: $^{13}C\{^1H\}$ NMR (126 MHz, C_6D_6) spectrum of *rac*-($^{2\text{-naphthyl}}$BDI*)ZnO^{i}Pr.

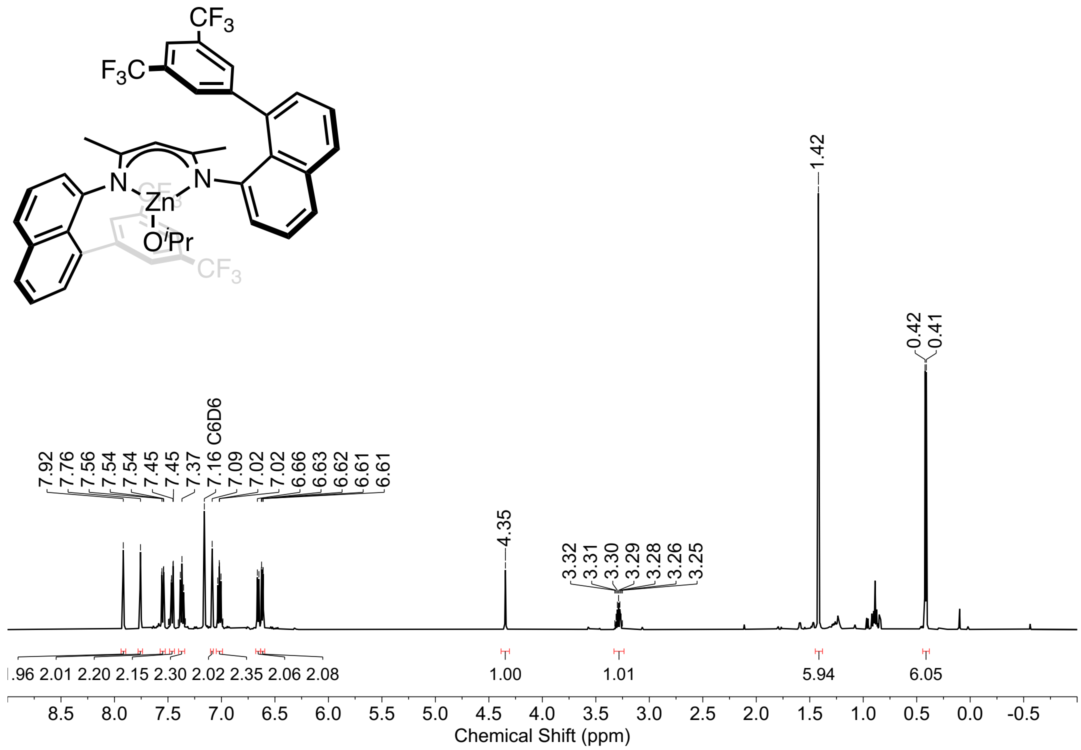

Figure S82: ^{1}H NMR (500 MHz, C_6D_6) spectrum of *rac*-($^{3,5\text{-}CF3}$BDI*)ZnO^{i}Pr.

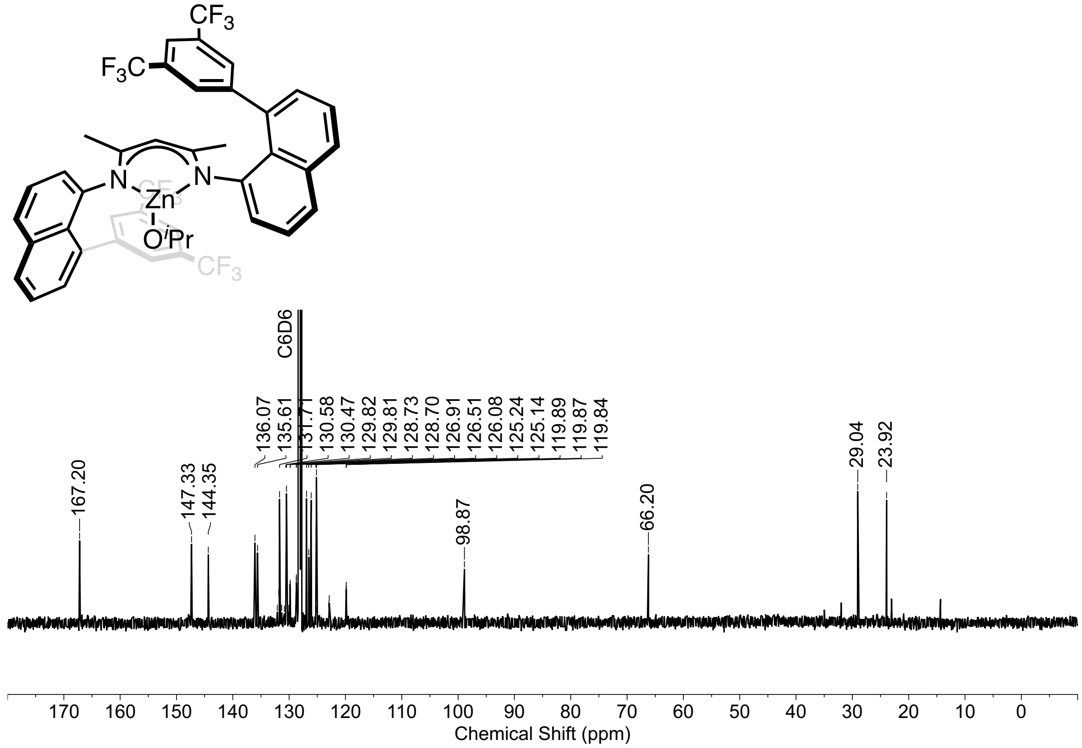

Figure S83: ^{13}C{^{1}H} NMR (126 MHz, C_6D_6) spectrum of *rac*-($^{3,5\text{-}CF3}$BDI*)ZnO^{i}Pr.

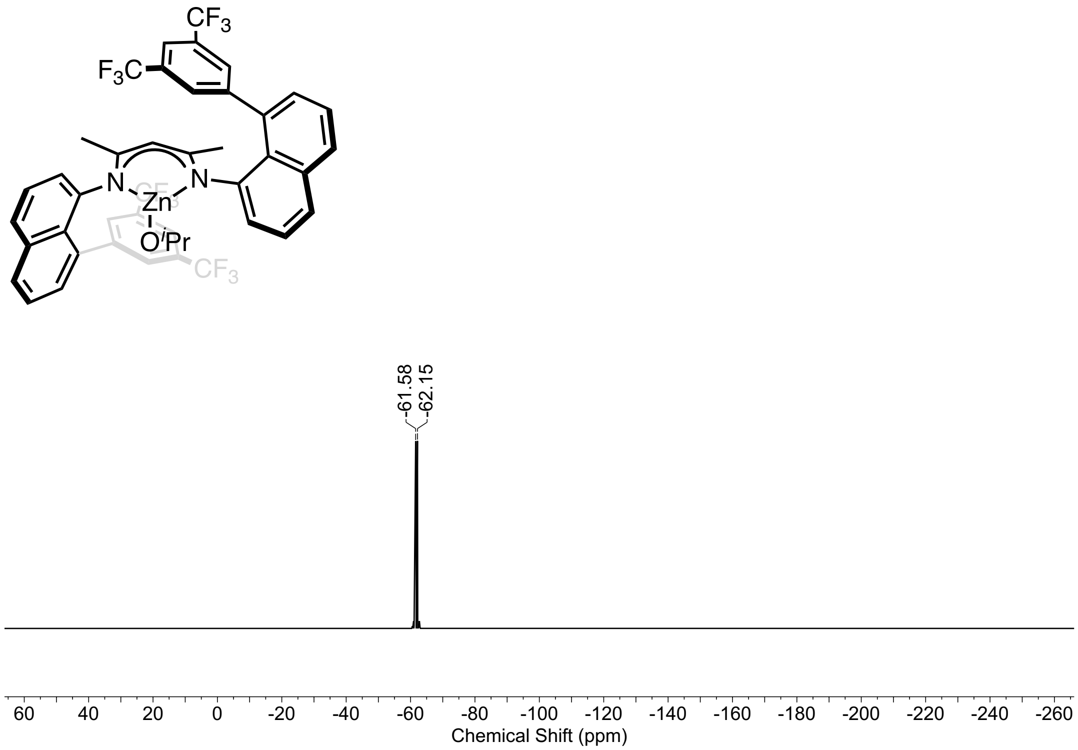

Figure S84: ^{19}F NMR (470 MHz, C_6D_6) spectrum of *rac*-($^{3,5\text{-}CF3}$BDI*)ZnO^{i}Pr.

4.4 [(^{iPr}BDI)ZnO^{i}Pr]$_2$ spectra

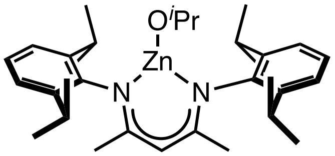

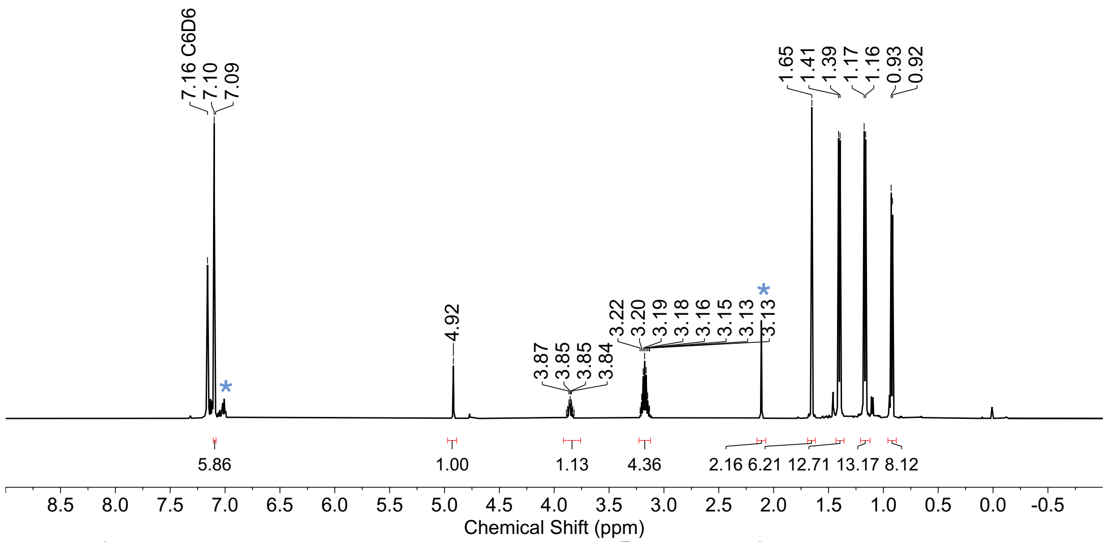

Figure S85: ^{1}H NMR (500 MHz, C_6D_6) spectrum of [(^{iPr}BDI)ZnO^{i}Pr]$_2$. *Residual PhMe.

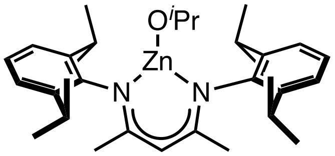

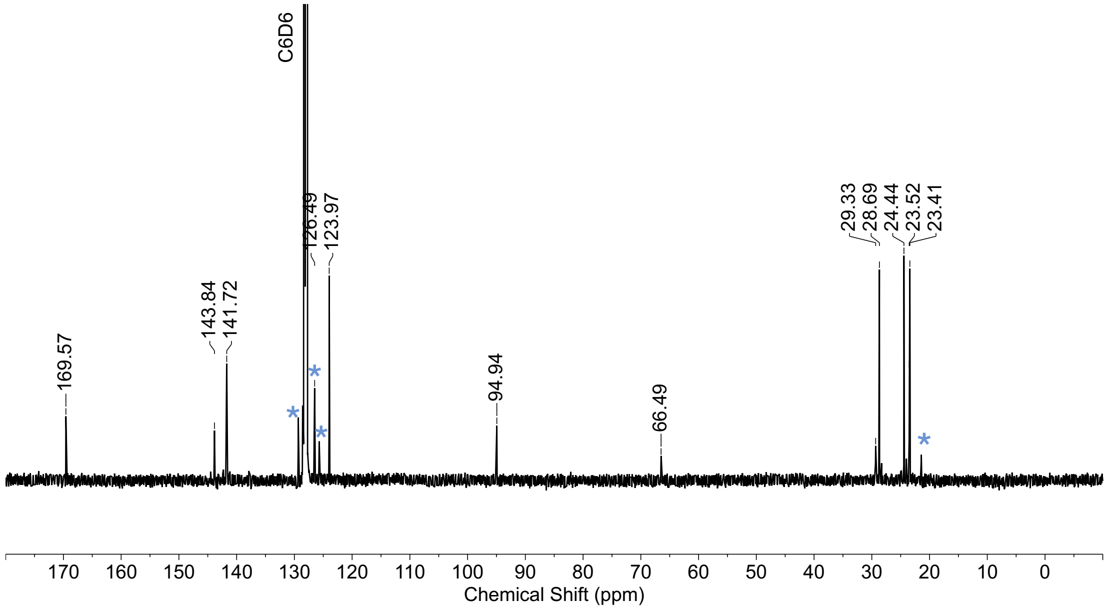

Figure S86: $^{13}C\{^1H\}$ NMR (126 MHz, C_6D_6) spectrum of [(^{iPr}BDI)ZnO^{i}Pr]$_2$. *Residual PhMe.

4.5 Selected *trans*-PHMB $^{13}C\{^1H\}$ NMR spectra

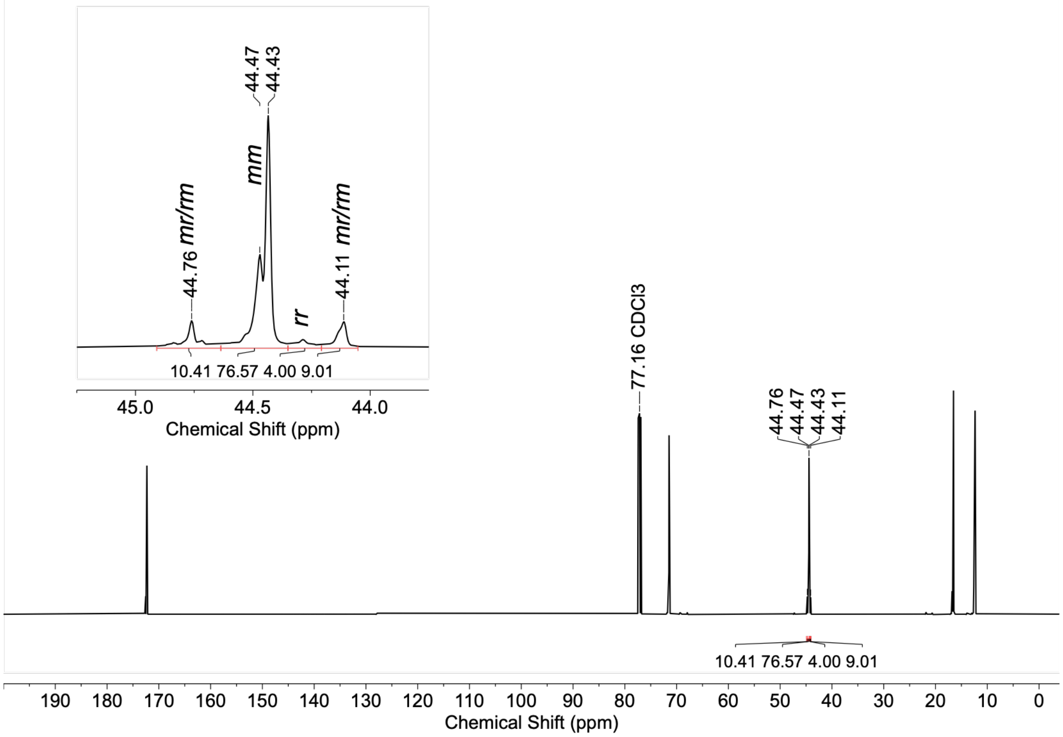

Figure S87: $^{13}C\{^1H\}$ NMR (126 MHz, $CDCl_3$) spectrum of isoenriched *trans*-PHMB (77 *mm*%; Table 1, entry 1).

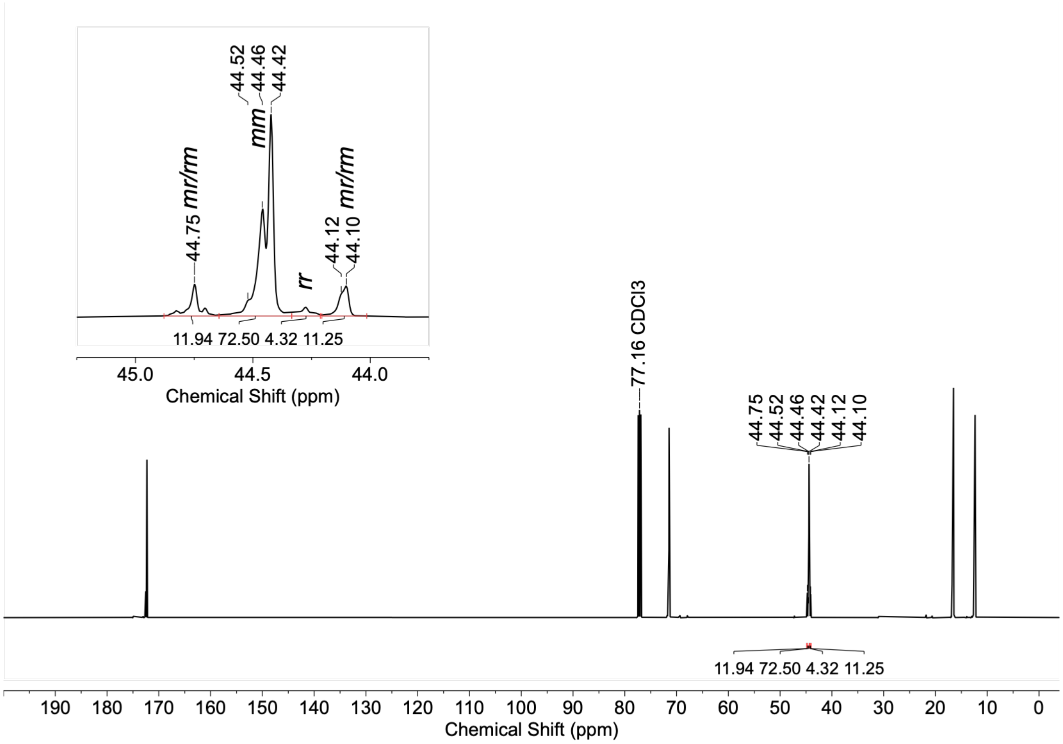

Figure S88: $^{13}C\{^1H\}$ NMR (126 MHz, $CDCl_3$) spectrum of isoenriched *trans*-PHMB (73 *mm*%; Table 1, entry 2).

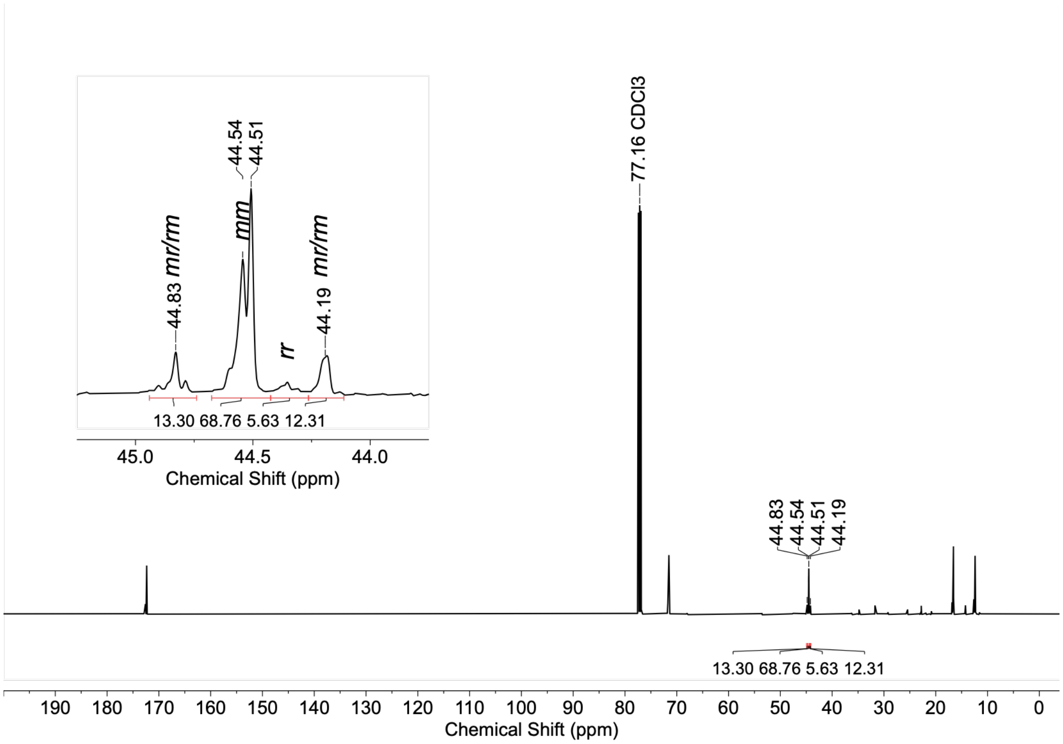

Figure S89: $^{13}C\{^1H\}$ NMR (126 MHz, $CDCl_3$) spectrum of isoenriched *trans*-PHMB (69 *mm*%; Table 1, entry 3).

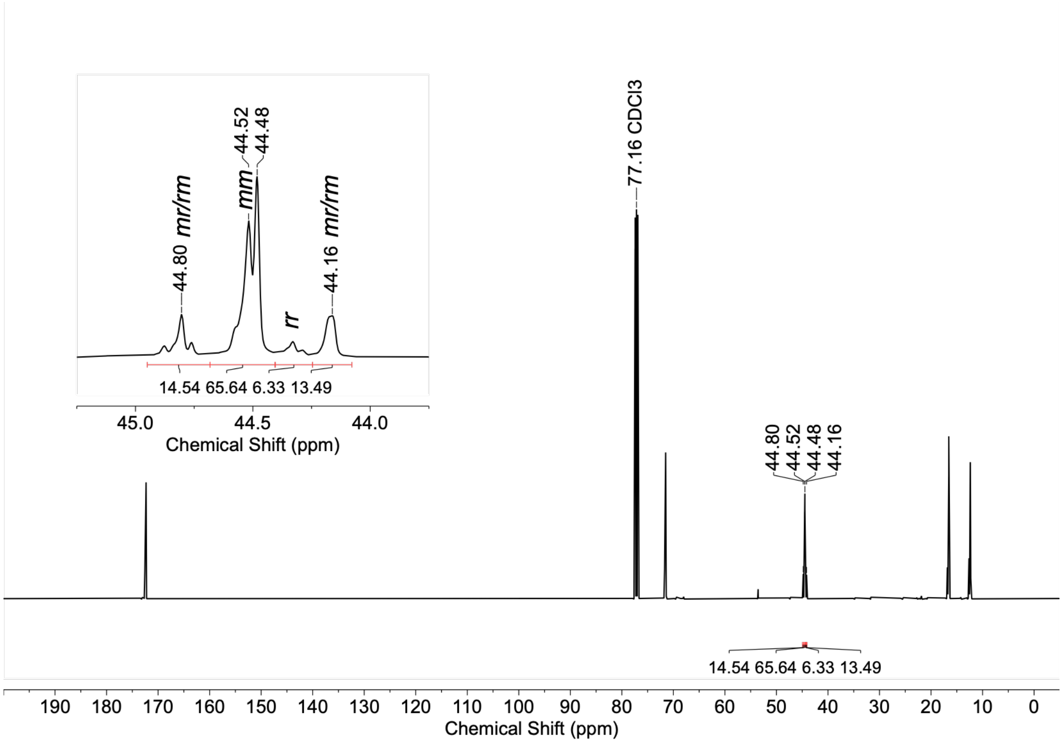

Figure S90: $^{13}C\{^1H\}$ NMR (126 MHz, $CDCl_3$) spectrum of isoenriched *trans*-PHMB (66 *mm*%; Table 1, entry 4).

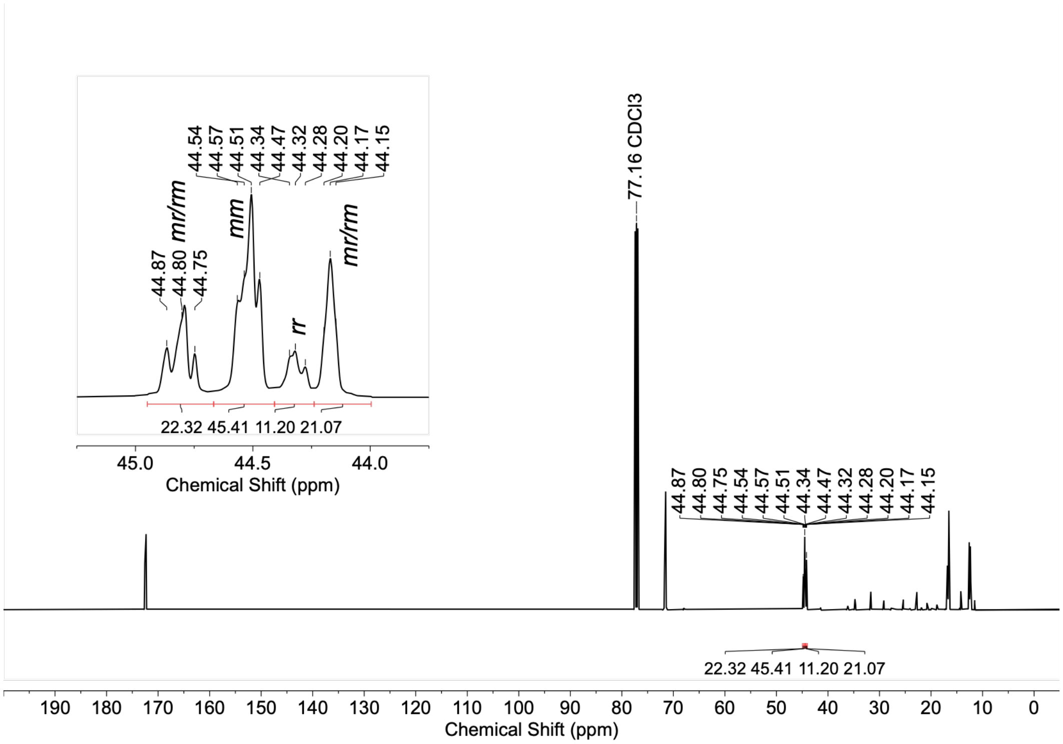

Figure S91: $^{13}C\{^1H\}$ NMR (126 MHz, $CDCl_3$) spectrum of isoenriched *trans*-PHMB (45 *mm*%; Table 1, entry 6).

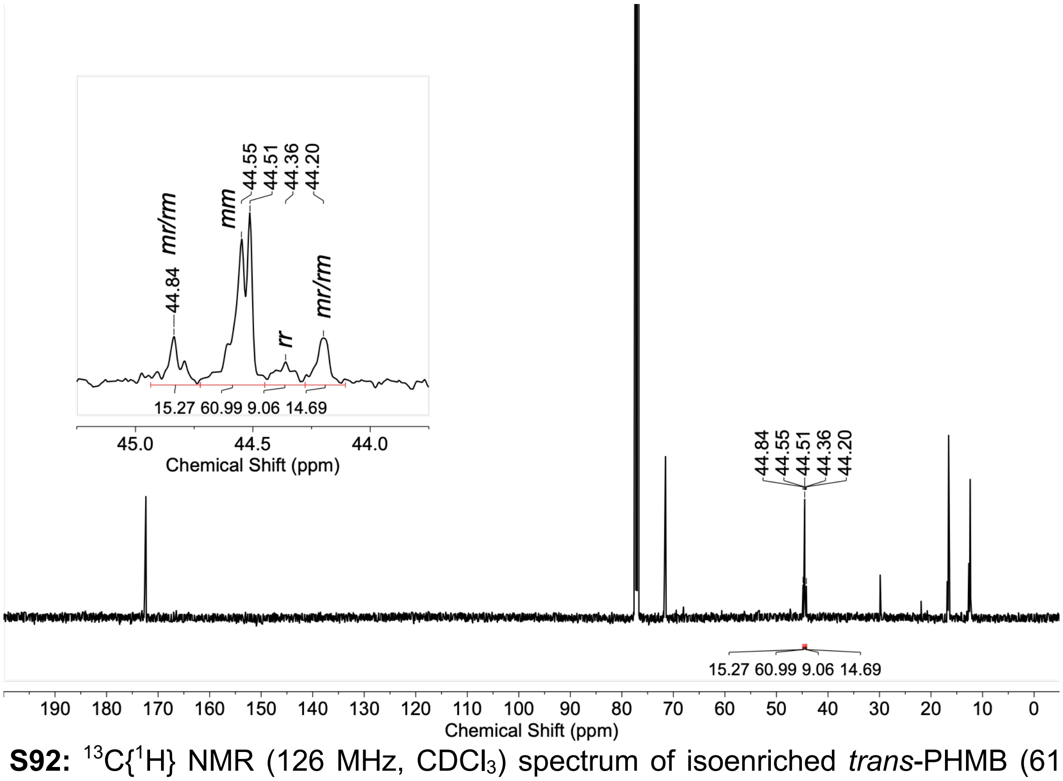

Figure S92: $^{13}C\{^1H\}$ NMR (126 MHz, $CDCl_3$) spectrum of isoenriched *trans*-PHMB (61 *mm*%; Table 1, entry 7).

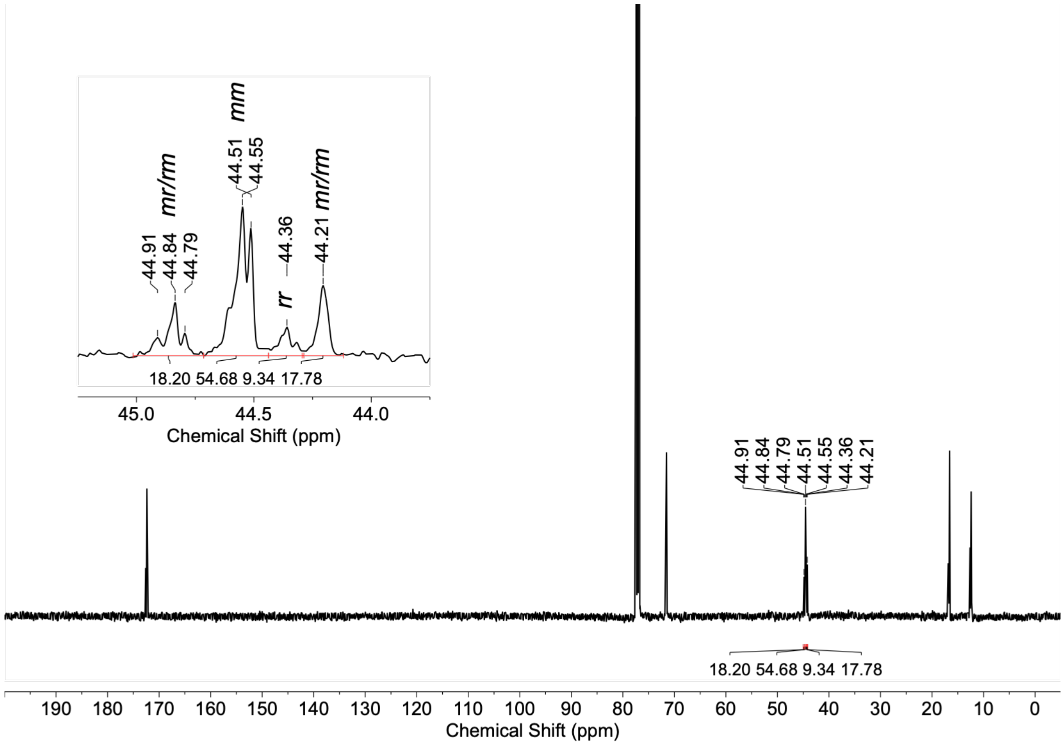

Figure S93: $^{13}C\{^{1}H\}$ NMR (126 MHz, $CDCl_3$) spectrum of isoenriched *trans*-PHMB (55 *mm*%; Table 1, entry 8).

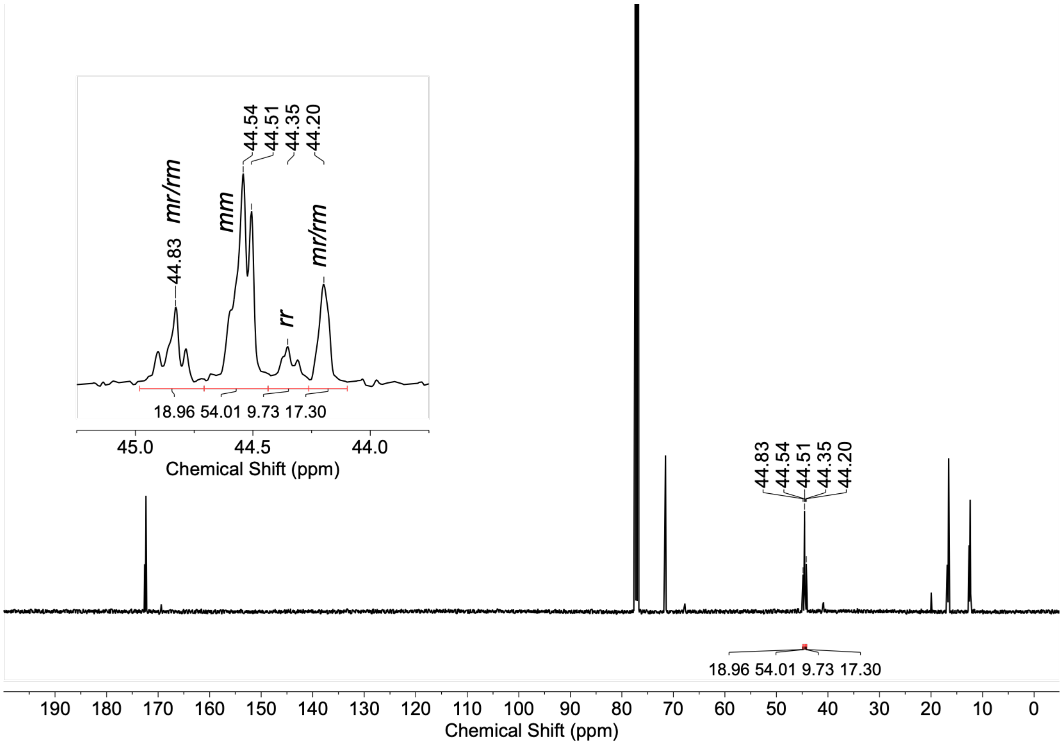

Figure S94: $^{13}C\{^{1}H\}$ NMR (126 MHz, $CDCl_3$) spectrum of isoenriched *trans*-PHMB (54 *mm*%; Table 1, entry 10).

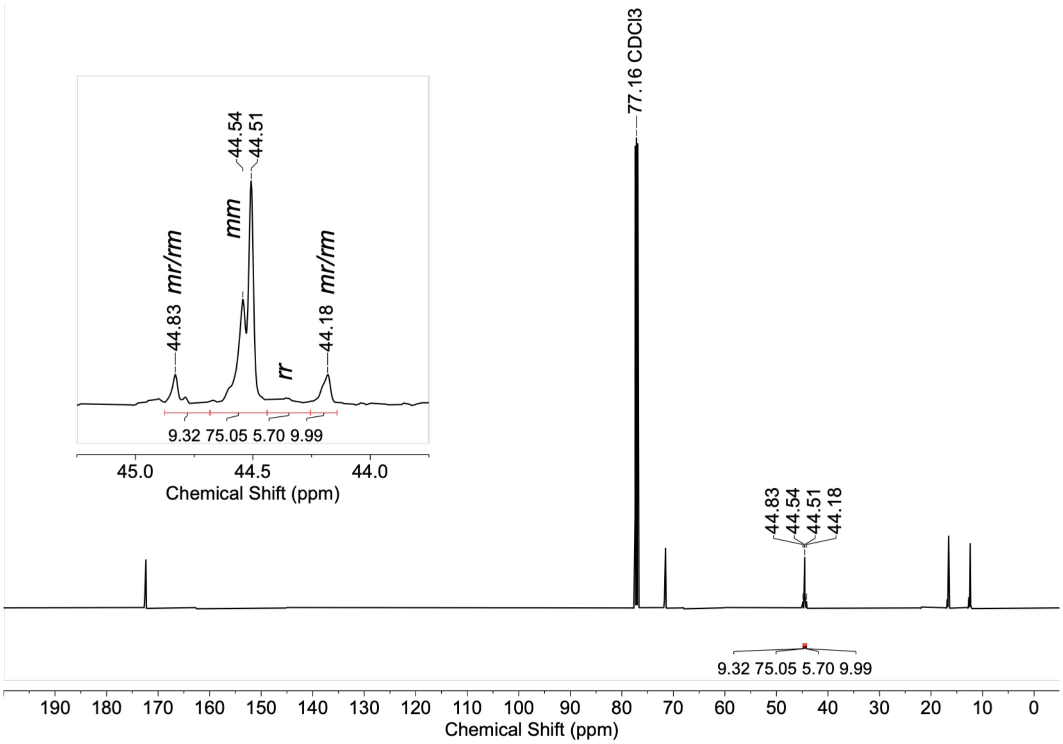

Figure S95: $^{13}C\{^{1}H\}$ NMR (126 MHz, $CDCl_3$) spectrum of isoenriched *trans*-PHMB (75 *mm*%; Table 1, entry 11).

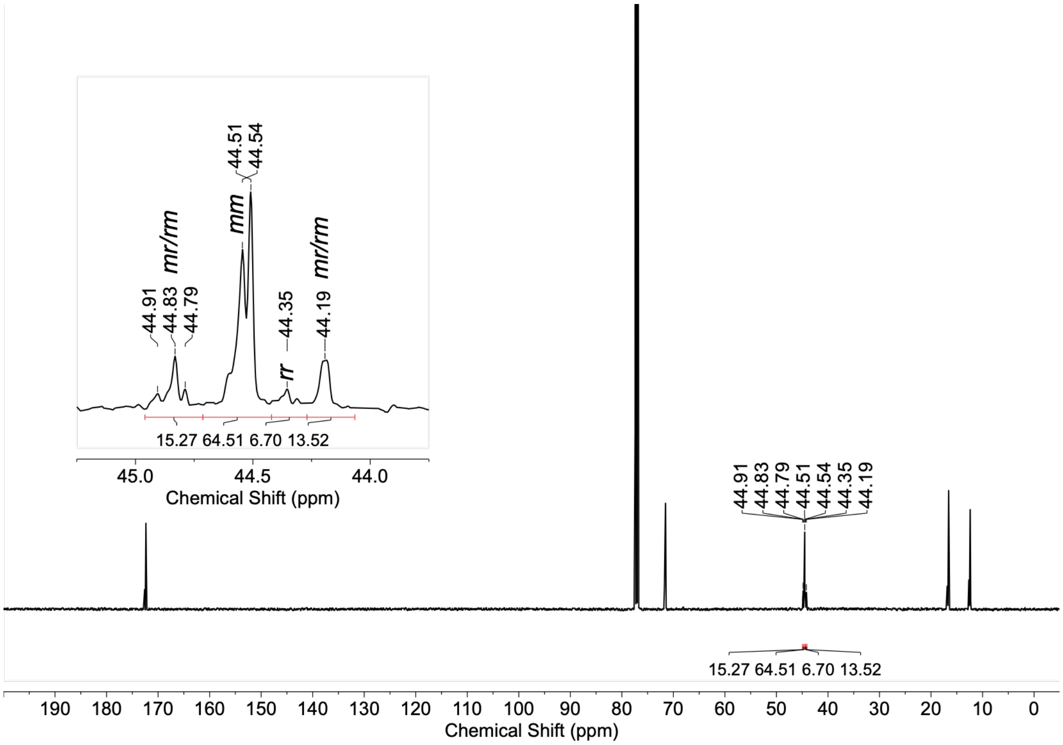

Figure S96: $^{13}C\{^{1}H\}$ NMR (126 MHz, $CDCl_3$) spectrum of isoenriched *trans*-PHMB (65 *mm*%; Table 1, entry 12).

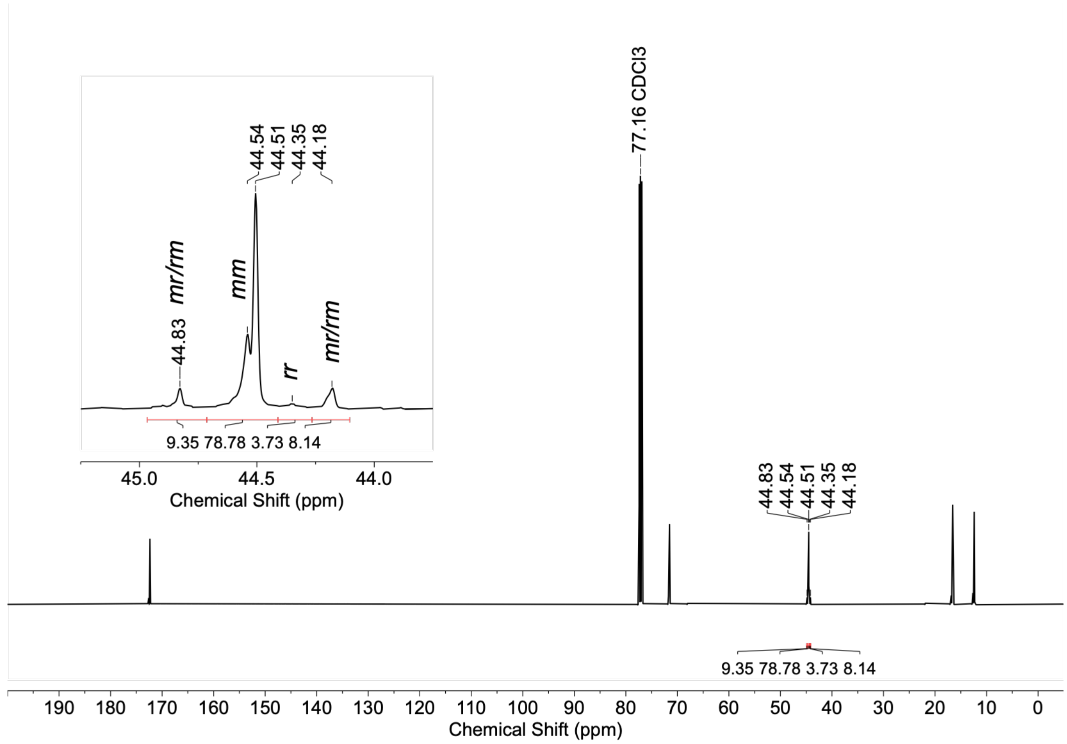

Figure S97: $^{13}C\{^1H\}$ NMR (126 MHz, $CDCl_3$) spectrum of isoenriched *trans*-PHMB (79 *mm*%; Table 1, entry 13).

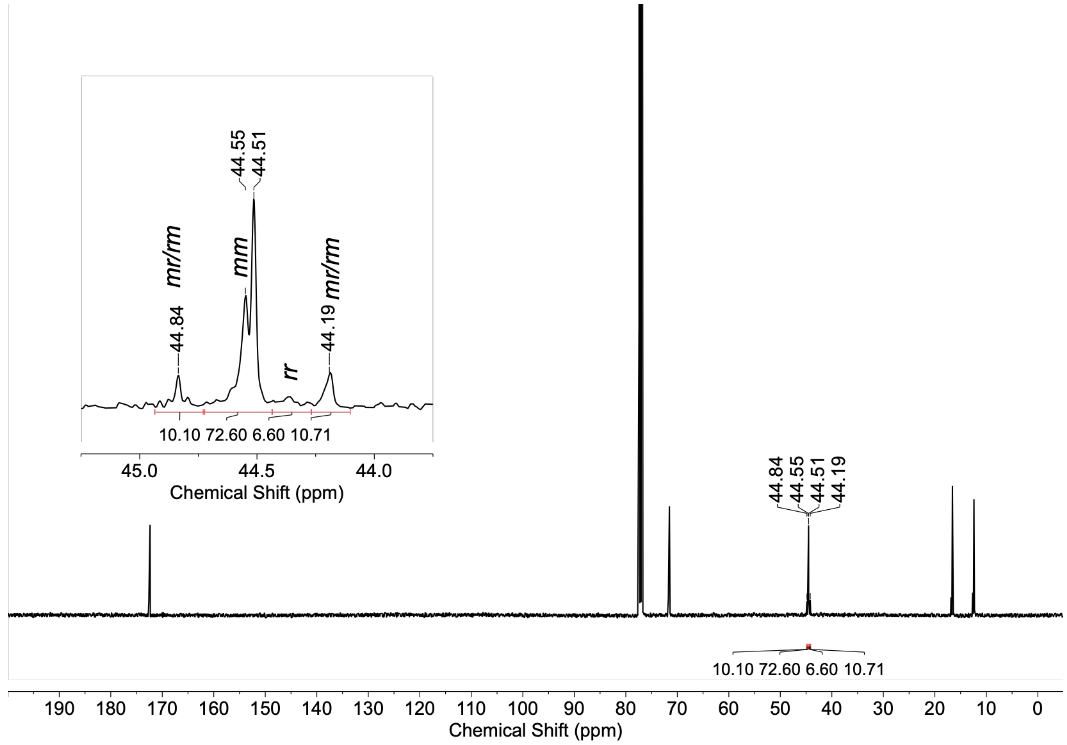

Figure S98: $^{13}C\{^1H\}$ NMR (126 MHz, $CDCl_3$) spectrum of isoenriched *trans*-PHMB (73 *mm*%; Table 1, entry 14).

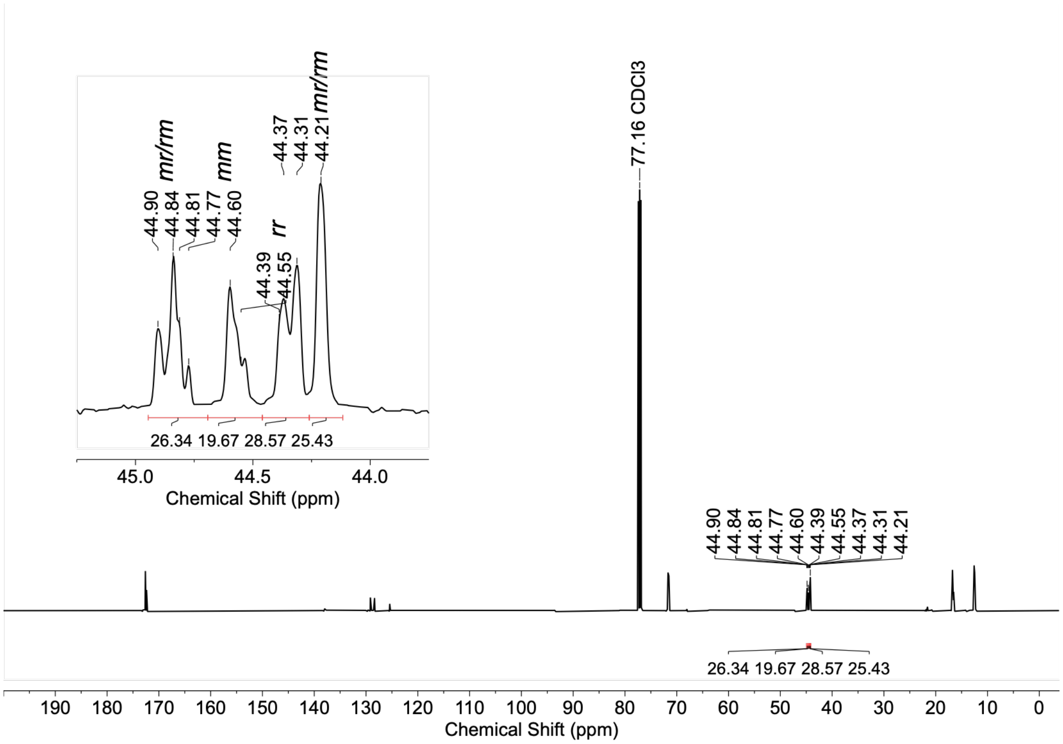

Figure S99: $^{13}C\{^1H\}$ NMR (126 MHz, $CDCl_3$) spectrum of atactic *trans*-PHMB made from *rac-trans*-DMPL with $[(^{iPr}BDI)ZnO^iPr]_2$ (Table S6, entry 1).

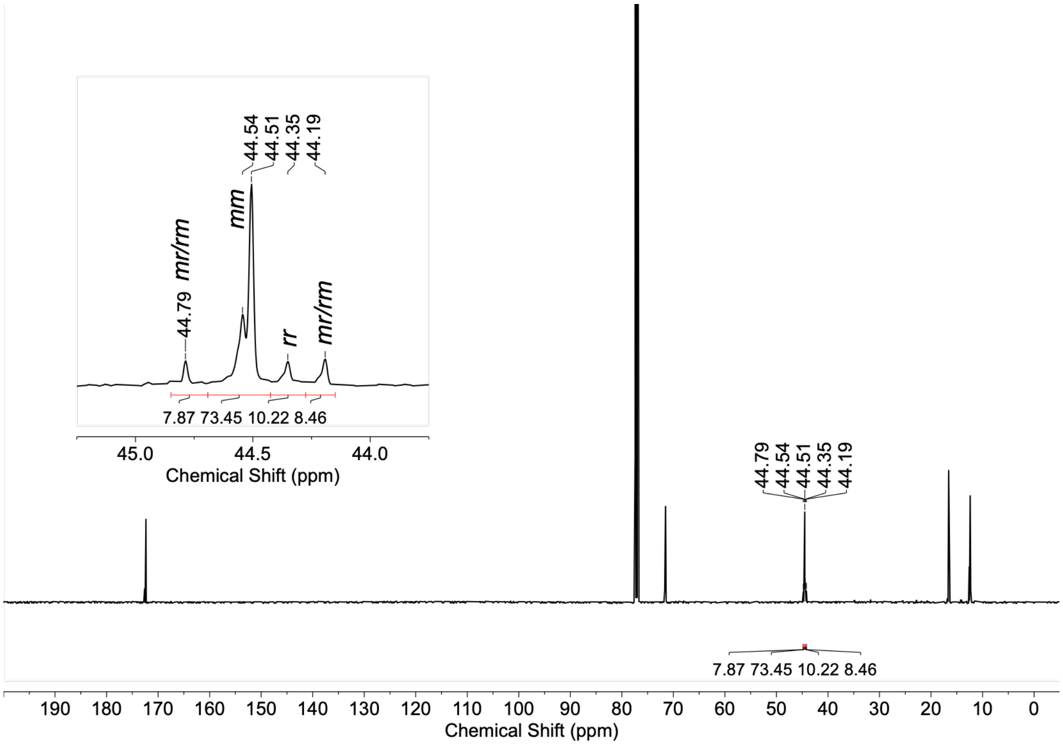

Figure S100: $^{13}C\{^1H\}$ NMR (126 MHz, $CDCl_3$) spectrum of isoenriched *trans*-PHMB made from 86 *%ee* (*R,R*)-*trans*-DMPL with $[(^{iPr}BDI)ZnO^iPr]_2$ (Table S6, entry 2).

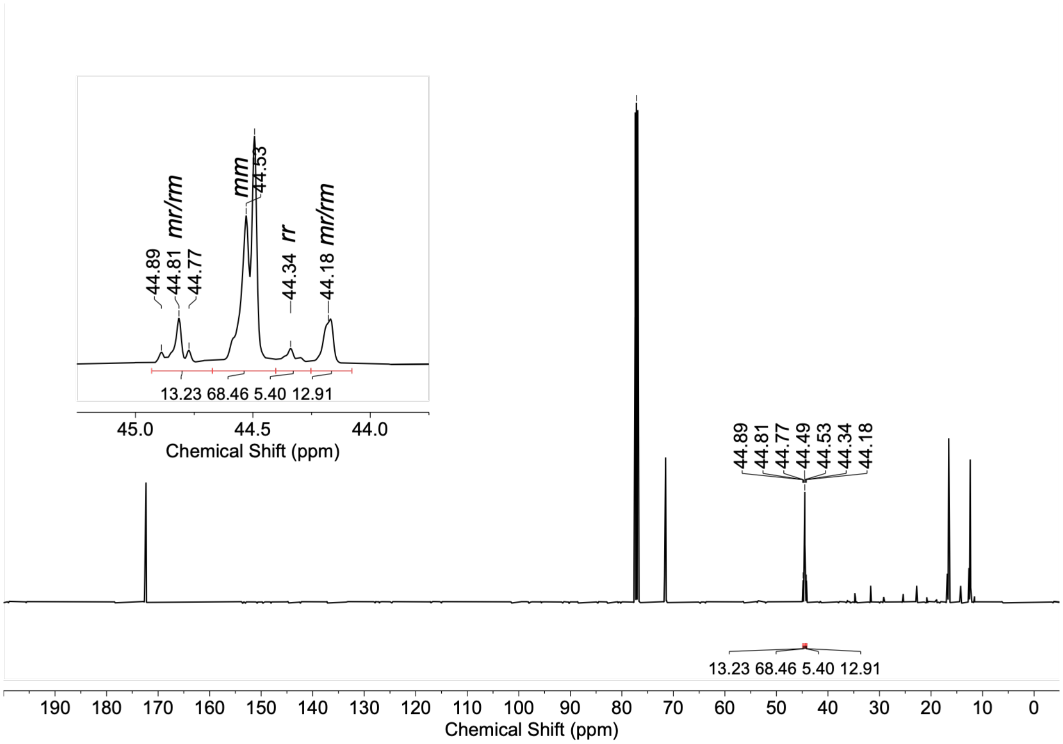

Figure S101: $^{13}C\{^{1}H\}$ NMR (126 MHz, $CDCl_3$) spectrum of high molecular weight, isoenriched *trans*-PHMB with 68 *mm*% used for tensile testing (Table S8, entry 1).

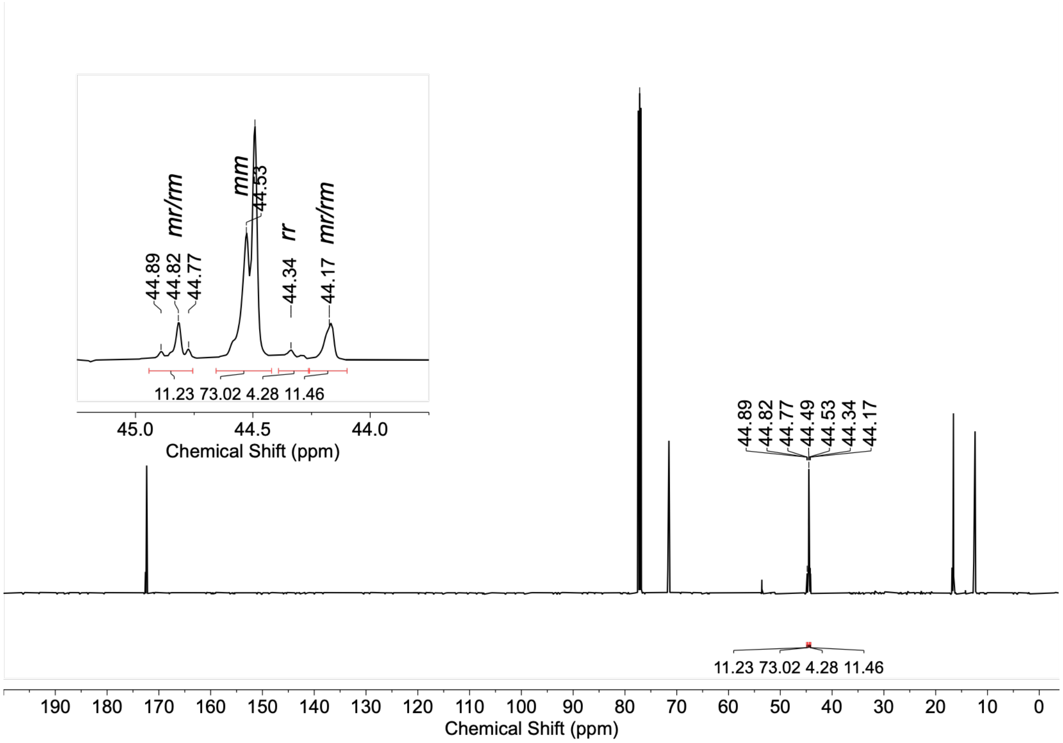

Figure S102: $^{13}C\{^{1}H\}$ NMR (126 MHz, $CDCl_3$) spectrum of high molecular weight, isoenriched *trans*-PHMB with 73 *mm*% used for tensile testing (Table S8, entry 2).

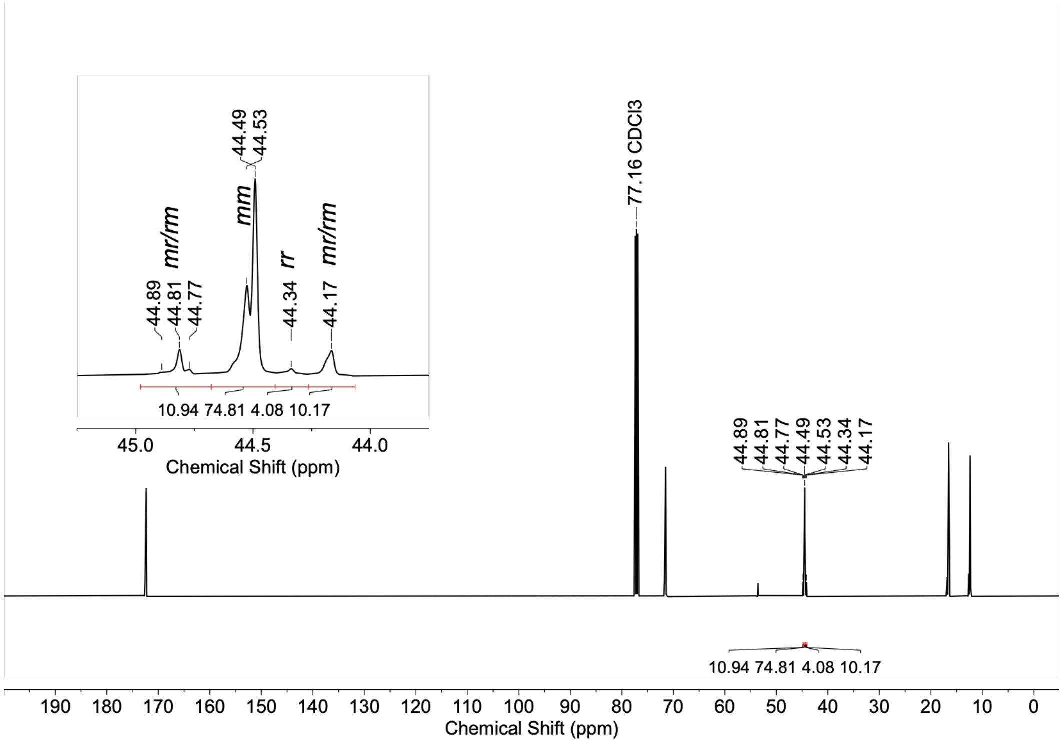

Figure S103: $^{13}C\{^1H\}$ NMR (126 MHz, $CDCl_3$) spectrum of high molecular weight, isoenriched *trans*-PHMB with 75 *mm*% used for tensile testing (Table S8, entry 3).

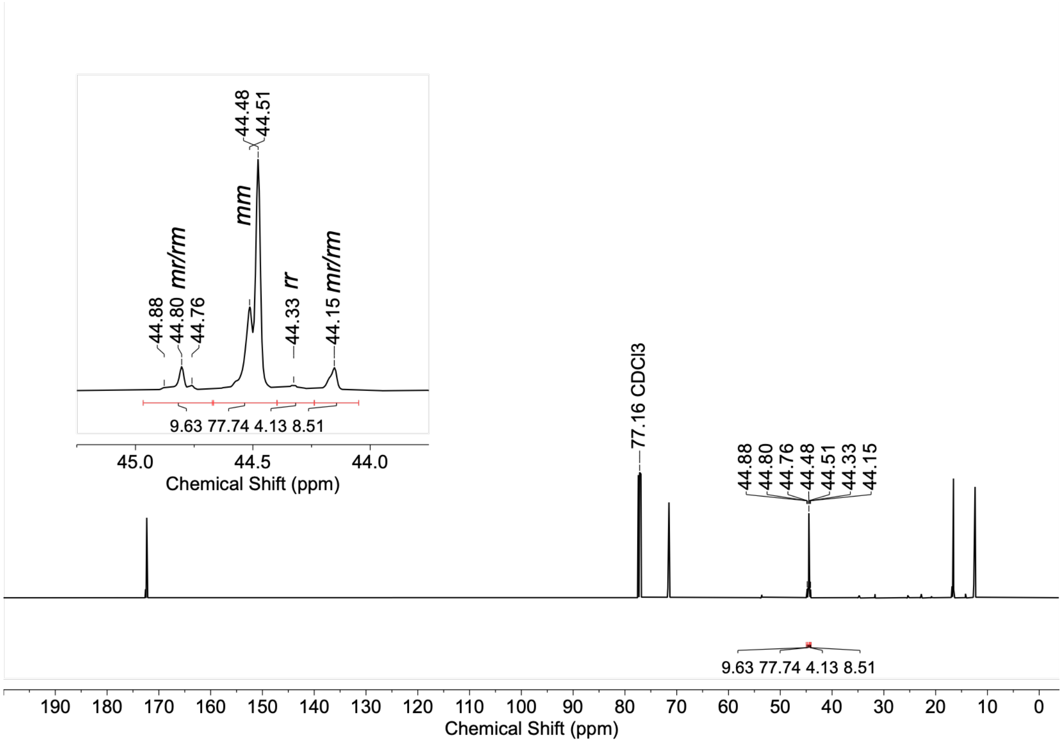

Figure S104: $^{13}C\{^1H\}$ NMR (126 MHz, $CDCl_3$) spectrum of high molecular weight, isoenriched *trans*-PHMB with 78 *mm*% used for tensile testing (Table S8, entry 4).

5 References

(1) Rowley, J. M.; Lobkovsky, E. B.; Coates, G. W. Catalytic Double Carbonylation of Epoxides to Succinic Anhydrides: Catalyst Discovery, Reaction Scope, and Mechanism. *J. Am. Chem. Soc.* **2007**, *129*, 4948–4960.

(2) Mulzer, M.; Lamb, J. R.; Nelson, Z.; Coates, G. W. Carbonylative Enantioselective *Meso*-Desymmetrization of *Cis*-Epoxides to *Trans*-β-Lactones: Effect of Salen-Ligand Electronic Variation on Enantioselectivity. *Chem. Commun.* **2014**, *50*, 9842–9845.

(3) Zhou, Z.; LaPointe, A. M.; Shaffer, T. D.; Coates, G. W. Nature-Inspired Methylated Polyhydroxybutyrates from C1 and C4 Feedstocks. *Nat. Chem.* **2023**, *15*, 856–861.

(4) Kramer, J. W.; Treitler, D. S.; Dunn, E. W.; Castro, P. M.; Roisnel, T.; Thomas, C. M.; Coates, G. W. Polymerization of Enantiopure Monomers Using Syndiospecific Catalysts: A New Approach To Sequence Control in Polymer Synthesis. *J. Am. Chem. Soc.* **2009**, *131*, 16042–16044.

(5) Yang, R.; Xu, G.; Lv, C.; Dong, B.; Zhou, L.; Wang, Q. $Zn(HMDS)_2$ as a Versatile Transesterification Catalyst for Polyesters Synthesis and Degradation toward a Circular Materials Economy Approach. *ACS Sustain. Chem. Eng.* **2020**, *8*, 18347–18353.

(6) Nadres, E. T.; Santos, G. I. F.; Shabashov, D.; Daugulis, O. Scope and Limitations of Auxiliary-Assisted, Palladium-Catalyzed Arylation and Alkylation of Sp^2 and Sp^3 C–H Bonds. *J. Org. Chem.* **2013**, *78*, 9689–9714.

(7) Zhang, D.; Nadres, E. T.; Brookhart, M.; Daugulis, O. Synthesis of Highly Branched Polyethylene Using "Sandwich" (8-*p*-Tolyl Naphthyl α-Diimine) Nickel(II) Catalysts. *Organometallics* **2013**, *32*, 5136–5143.

(8) Chen, Z.; Mesgar, M.; White, P. S.; Daugulis, O.; Brookhart, M. Synthesis of Branched Ultrahigh-Molecular-Weight Polyethylene Using Highly Active Neutral, Single-Component Ni(II) Catalysts. *ACS Catal.* **2015**, *5*, 631–636.

(9) Padilla-Vélez, O.; O'Connor, K. S.; LaPointe, A. M.; MacMillan, S. N.; Coates, G. W. Switchable Living Nickel(II) α-Diimine Catalyst for Ethylene Polymerisation. *Chem. Commun.* **2019**, *55*, 7607–7610.

(10) Klimovica, K.; Kirschbaum, K.; Daugulis, O. Synthesis and Properties of "Sandwich" Diimine-Coinage Metal Ethylene Complexes. *Organometallics* **2016**, *35*, 2938–2943.

(11) Mecking, S.; Riedmiller, K.; Scholliers, J. S.; Schnitte, M. Remote Perfluoroalkyl Substituents are Key to Living Aqueous Ethylene Polymerization *Angew. Chem., Int. Ed.* **2020**, *59*, 3258–3263.

(12) Feldman, J.; McLain, S. J.; Parthasarathy, A.; Marshall, W. J.; Calabrese, J. C.; Arthur, S. D. Electrophilic Metal Precursors and a β-Diimine Ligand for Nickel(II)- and Palladium(II)-Catalyzed Ethylene Polymerization. *Organometallics* **1997**, *16*, 1514–1516.

(13) Cheng, M.; Moore, D. R.; Reczek, J. J.; Chamberlain, B. M.; Lobkovsky, E. B.; Coates, G. W. Single-Site β-Diiminate Zinc Catalysts for the Alternating Copolymerization of CO_2 and Epoxides: Catalyst Synthesis and Unprecedented Polymerization Activity. *J. Am. Chem. Soc.* **2001**, *123*, 8738–8749.

(14) Furutate, S.; Kamoi, J.; Nomura, C. T.; Taguchi, S.; Abe, H.; Tsuge, T. Superior Thermal Stability and Fast Crystallization Behavior of a Novel, Biodegradable α-Methylated Bacterial Polyester. *NPG Asia Mater.* **2021**, *13*, 31.

(15) Tian, J.-J.; Li, R.; Quinn, E. C.; Nam, J.; Chokkapu, E. R.; Zhang, Z.; Zhou, L.; Gowda, R. R.; Chen, E. Y.-X. Stereodivergent Transformation of a Natural Polyester to Enantiopure PHAs. *Nature* **2025**, *643*, 967–974.

(16) Abe, H. Thermal Degradation of Environmentally Degradable Poly(Hydroxyalkanoic Acid)s. *Macromol. Biosci.* **2006**, *6*, 469–486.
